# Effectiveness of Renin-Angiotensin-Aldosterone System Blockade on Residual Kidney Function and Peritoneal Membrane Function in Peritoneal Dialysis Patients: A Network Meta-Analysis

**DOI:** 10.1038/s41598-019-55561-5

**Published:** 2019-12-20

**Authors:** Sirayut Phatthanasobhon, Surapon Nochaiwong, Kednapa Thavorn, Kajohnsak Noppakun, Setthapon Panyathong, Yuttitham Suteeka, Brian Hutton, Manish M. Sood, Greg A. Knoll, Chidchanok Ruengorn

**Affiliations:** 1 Doctor of Philosophy (Pharmacy) Program, Faculty of Pharmacy, Chiang Mai University, Chiang Mai, 50200 Thailand; 2 Department of Pharmaceutical Care, Faculty of Pharmacy, Chiang Mai University, Chiang Mai, 50200 Thailand; 3 Pharmacoepidemiology and Statistics Research Center (PESRC), Faculty of Pharmacy, Chiang Mai University, Chiang Mai, 50200 Thailand; 4 Ottawa Hospital Research Institute, Ottawa Hospital, Ottawa, Ontario K1H 8L6 Canada; 5 Institute of Clinical and Evaluative Sciences, ICES uOttawa, Ottawa, Ontario K1Y 4E9 Canada; 6 School of Epidemiology and Public Health, Faculty of Medicine, University of Ottawa, Ottawa, Ontario K1G 5Z3 Canada; 7 Division of Nephrology, Department of Internal Medicine, Faculty of Medicine, Chiang Mai University, Chiang Mai, 50200 Thailand; 8 Kidney Center, Nakornping Hospital, Chiang Mai, 50180 Thailand; 9 Division of Nephrology, Department of Medicine, University of Ottawa, Ottawa, Ontario Canada

**Keywords:** Nephrology, Peritoneal dialysis

## Abstract

We performed a network meta-analysis of randomised controlled trials (RCTs) and non-randomised studies in adult peritoneal dialysis patients to evaluate the effects of specific renin-angiotensin aldosterone systems (RAAS) blockade classes on residual kidney function and peritoneal membrane function. Key outcome parameters included the following: residual glomerular filtration rate (rGFR), urine volume, anuria, dialysate-to-plasma creatinine ratio (D/P Cr), and acceptability of treatment. Indirect treatment effects were compared using random-effects model. Pooled standardised mean differences (SMDs) and odd ratios (ORs) were estimated with 95% confidence intervals (CIs). We identified 10 RCTs (n = 484) and 10 non-randomised studies (n = 3,305). Regarding changes in rGFR, RAAS blockade with angiotensin-converting enzyme inhibitors (ACEIs) and angiotensin II receptor blockers (ARBs) were more efficacious than active control (SMD 0.55 [0.06–1.04] and 0.62 [0.19–1.04], respectively) with the protective effect on rGFR observed only after usage ≥12 months, and no differences among ACEIs and ARBs. Compared with active control, only ACEIs showed a significantly decreased risk of anuria (OR 0.62 [0.41–0.95]). No difference among treatments for urine volume and acceptability of treatment were observed, whereas evidence for D/P Cr is inconclusive. The small number of randomised studies and differences in outcome definitions used may limit the quality of the evidence.

## Introduction

Despite improvement in the treatments and techniques for peritoneal dialysis (PD) patients, long-term PD leads to the decline of residual kidney function (RKF) and peritoneal membrane function (PMF), as a result of membrane or ultrafiltration (UF) failure^1,2^. Existing epidemiological studies have illustrated that RKF deteriorations over time in PD patients compromising patient survival as well as overall health-related quality of life (HRQOL)^3–5^. The CANUSA (Canada-United States Peritoneal Dialysis) study, the landmark multicenter prospective cohort of incident PD patients, showed 12% and 36% reductions in the risk of death for each 5 L/week/1.73 m^2^ increment in estimated glomerular filtration rate (eGFR) and each 250 mL increase in urine volume, respectively^3^. Likewise, the risk of UF failure has increased 3–5% in the first year and 30–50% after three years of PD^6–8^. There is increasing evidence on the inter-relationship between the RKF and PMF^9^. Alterations in RKF and peritoneal characteristics over time are important determinants of patients’ technique survival and mortality^9,10^. Subsequently, treatment strategies for maintaining RKF in conjunction with PMF are crucial.

Blockade of the renin-angiotensin aldosterone systems (RAAS) with angiotensin-converting enzyme inhibitors (ACEIs), angiotensin II receptor blockers (ARBs), and mineralocorticoid receptor antagonists (MRAs) in PD patients are likely to preserve residual glomerular filtration rate (rGFR) along with residual urine volume until the PD patients reach anuria that may improve survival in these population^11–15^. Several studies revealed that blockade of RAAS positively effects the peritoneal membrane by reducing morphologic changes and preserving peritoneal membrane integrity^16–18^. Therefore, inhibitions of RAAS could potentially improve technique survival and allow patients to be sustained on PD programs for longer periods.

However, the role of RAAS blockade in PD patients has not been fully elucidated. Some studies have revealed the protective properties^11–15,19^, whereas others have not^20–26^. Previous systematic reviews have shown that ACEIs/ARBs substantially benefit in preserving rGFR in PD patients, while a lack of evidence exist regarding the relative efficacy of MRAs and direct renin inhibitors (DRIs)^27–29^. Moreover, existing pairwise meta-analyses have focused mainly on RKF rather than other clinically relevant and related outcomes such as PMF and adverse events^27–29^. Recently, treatment with an ACEIs or ARBs has been recommended by the International Society for Peritoneal Dialysis (ISPD)^30^ for PD patients with significant RKF, although the comparative effectiveness of specific RAAS blockade classes remains unknown.

To address this knowledge gap, we conducted a systematic review and network meta-analysis (NMA) of randomised controlled trials (RCTs) and non-randomised studies in PD patients to evaluate the effects of specific RAAS blockade classes on RKF and PMF as determined by five key parameters: rGFR, urine volume, incidence of anuria, dialysate-to-plasma creatinine ratio (D/P Cr), and acceptability of treatment.

## Results

### Search strategy and characteristics of included studies

**Figure 1:**
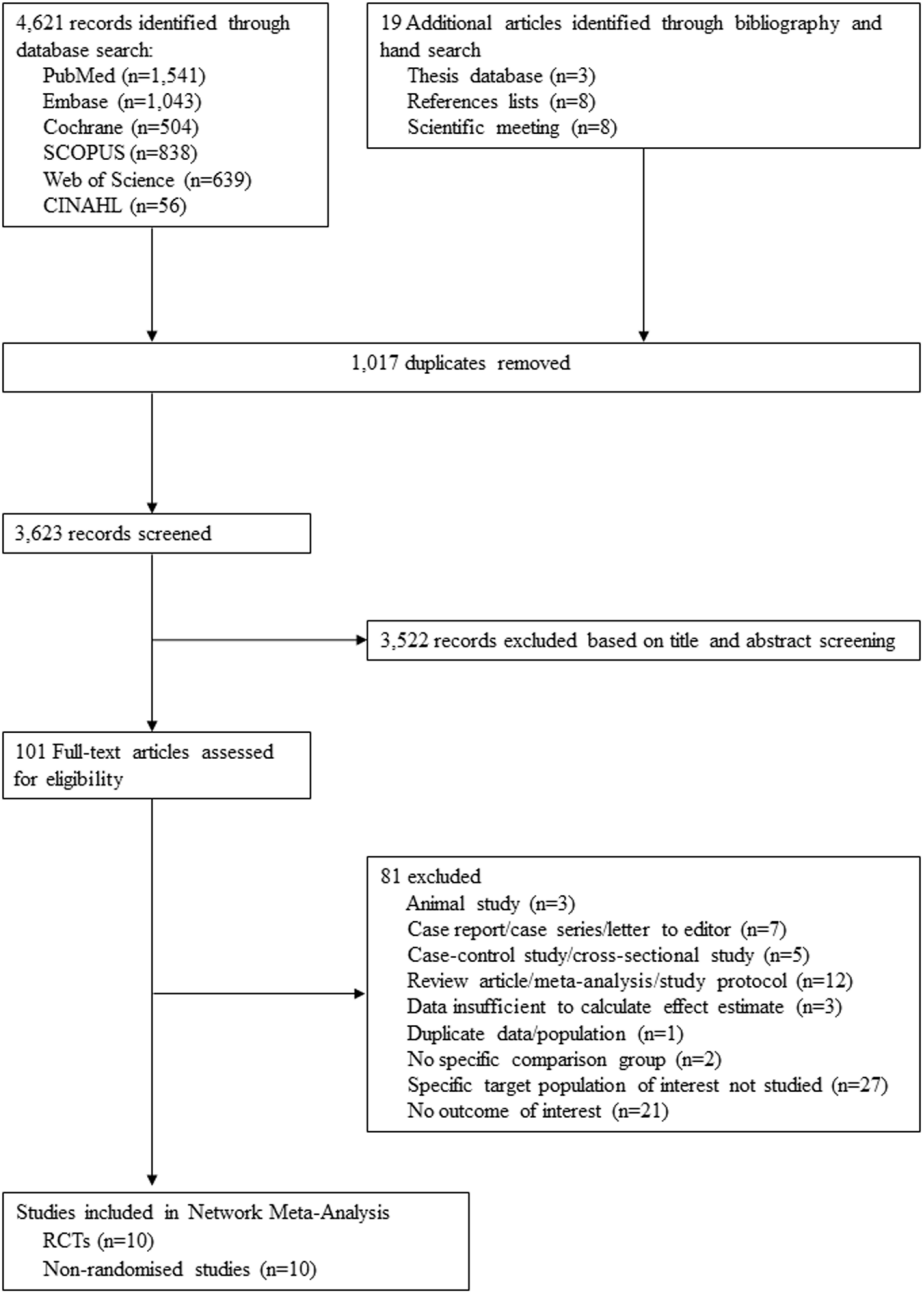
Selection of studies. Abbreviations: RCTs, randomised-controlled trials.

**Figure 2:**
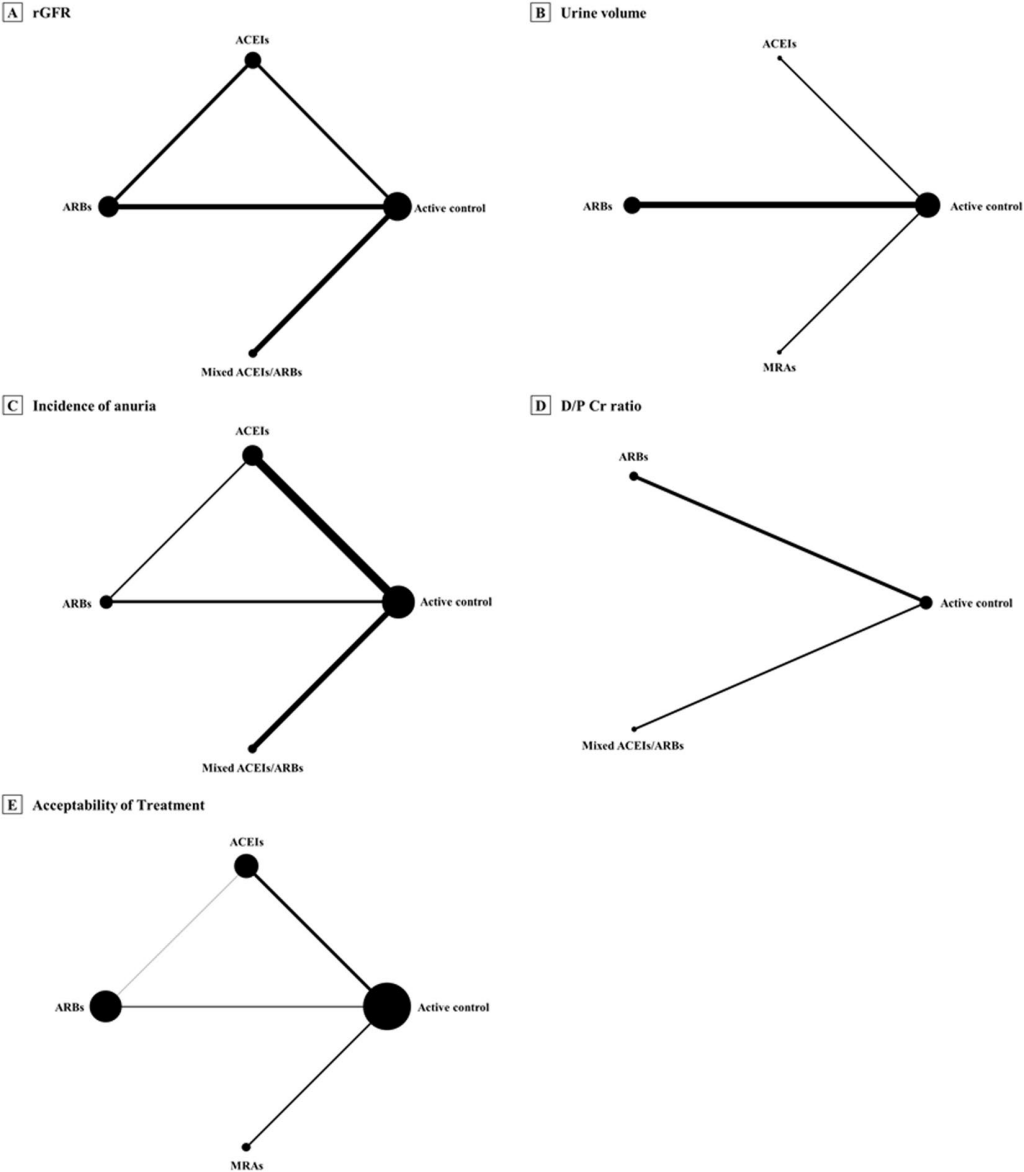
Network plot of eligible comparisons for primary outcomes. Notes: The circles (nodes) represent the available treatments and the lines (edges) represent the available comparisons. Size nodes and width of edges indicate weighting according to the numbers of studies involved for each treatment and comparison, respectively. Abbreviations: ACEIs, angiotensin-converting enzyme inhibitors; ARBs, angiotensin II receptor blockers; D/P Cr, dialysate-to-plasma creatinine; MRAs, mineralocorticoid receptor antagonists; rGFR, residual glomerular filtration rate.

The systematic search details are described in Fig. 1. After screening of titles/abstracts, 101 full-text articles of potentially relevant studies were acquired. After appraising these articles against study inclusion/exclusion criteria (Supplementary, Table S1), we included 10 RCTs^12–14,25,31–36^ and 10 non-randomised studies^11,20–24,26,37–39^ that compared RAAS blockade classes with active control (Table 1). Four RAAS blockade classes were compared with active control—ACEIs, ARBs MRAs, and mixed ACEIs/ARBs, however, no data for DRIs in any outcome of interest. Two studies provided direct comparisons of ACEIs and ARBs^31,36^. Network diagrams presenting the available evidence for primary and secondary outcomes are illustrated in Figs. 2 and S1. Detailed methods of measurement and definition of outcomes are described in Supplementary Table S2. A total of 3,789 PD participants were enrolled in the set of included studies; the majority of these patients received continuous ambulatory peritoneal dialysis (CAPD). The baseline mean age and rGFR ranged from 40.2–66.8 years and 0.6–8.4 mL/min/1.73 m^2^, respectively. The follow-up periods ranged from 7 days to 66.3 months, and 12 (60%) studies encompassed participants from Asia. The study- and participant-characteristics are illustrated in Table 1 and Supplementary, Table S3.

**Table 1:** Description of included studies: RCTs and non-randomised studies.

Author, Year	Design	Country Enrollment	Sample Size	Intervention	Control	Mean Age ± SD, Year	Female, N (%)	Mean rGFR ± SD, mL/min	Mean Urine Volume ± SD, mL/day	PD Modality	Follow-Up Period, Mean ± SD	Risk of Bias^a^
Favazza et al., 1992^32^	RCT: open label, crossover study	Italy	9	Enalapril (40 mg/day)	Nifedipine (60 mg/day), Clonidine (0.45 mg/day)	64.0 ± 5.4	3 (33.3)	3.9 ± 0.8	NR	CAPD	14 days	1/8
Moist et al., 2000^11^	Non-randomised studies: prospective cohort study	USA	1,032	ACEI user	Non-ACEI users	55.5 ± 14.6	490 (47.5)	7.5 ± 2.7^b^	NR	CAPD, APD	11.9 ± 1.7 months	7/9
Johnson et al., 2003^37^	Non-randomised studies: prospective cohort study	Australia	146	ACEI users	Non-ACEI users	54.8 ± 16.3	83 (56.8)	4.9 ± 2.3^b^	NR	CAPD, APD	20.5 ± 14.8 months	7/9
Li et al., 2003^12^	RCT: open-label, parallel study	Hong Kong	60	Ramipril (5 mg/day)	Active control^c^	58.6 ± 12.1	22 (36.7)	3.6 ± 2.0^b^	NR	CAPD	12 months	3/8
Phakdee-kitcharoen et al., 2004^d ^^31^	RCT: open label, crossover study	Thailand	21	Candesartan (8 mg/day)	Enalapril (10 mg/day)	44.8 ± 10.1	7 (33.3)	2.0 ± 2.4	NR	CAPD	1 months	1/8
Suzuki et al., 2004^13^	RCT: open-label, parallel study	Japan	34	Valsartan (40–80 mg/day)	Active control^c^	63.5 ± 3.5	16 (47.0)	4.3 ± 1.7^b^	1045.0 ± 220.6	CAPD	24 months	3/8
Rojas-Campos et al., 2005^20^	Non-randomised studies: quasi experimental (crossover) study	Mexico	20	Losartan (50–200 mg/day)	Prazosin (2–6 mg/day), verapamil (80–240 mg/day)	42.9 ± 16.6	4 (20.0)	NR	NR	CAPD	7 days	1/8
Wang et al., 2005^33^	RCT: open-label, parallel study	China	32	Valsartan (40–80 mg/day)	Active control^c^	42.0 ± 11.5	12 (35.3)	4.9 ± 2.2^b^	1085 ± 696.3	CAPD	28 ± 13 months	1/8
Furuya et al., 2006^21^	Non-randomised studies: quasi experimental (crossover) study	Japan	8	Candesartan (8 mg/day)	Active control^c^	66.8 ± 8.8	4 (50.0)	NR	1035 ± 383.5	CAPD, APD	3 months	1/8
Jearnsujitwimol et al., 2006^39^	Non-randomised studies: quasi experimental (crossover) study	Thailand	7	Candesartan (8–16 mg/day)	Active control^c^	62.0 ± 3.6	2 (28.6)	0.6 ± 0.4	16.9 ± 8.2	CAPD	12 weeks for treatment, 6 week for control	1/8
Zhong et al., 2007^34^	RCT: open-label, parallel study	China	44	Irbesartan (300 mg/day)	Active control^c^	44.0 ± 14.6	14 (31.8)	4.5 ± 2.7^b^	1255 ± 425.1	CAPD	12 months	1/8
Wontanatawatot et al., 2009^35^	RCT: open-label, parallel study	Thailand	46	Enalapril (40 mg/day)	Active control^c^	48.1 ± 12.0	25 (54.3)	NR	NR	CAPD	6 months	1/8
Jing et al., 2010^22^	Non-randomised studies: retrospective cohort study	China	66	ACEI/ARB users	Non-ACEI/ARB users	52.5 ± 12.2	24 (36.4)	4.6 ± 2.7	NR	CAPD	12 months	6/9
Kolesnyk et al., 2011^23^	Non-randomised studies: prospective cohort study	Netherland	452	ACEI/ARB users	Non-ACEI/ARB users	50.8 ± 10.6	154 (34.1)	4.9 ± 2.4^b^	NR	Not specified	3 years	8/9
Basturk et al., 2012^24^	Non-randomised studies: prospective cohort study	Turkey	43	ACEI users	Non-ACEI users	40.2 ± 18.7	19 (44.2)	NR	332 ± 476.3	CAPD	6 months	6/9
Reyes-Marín et al., 2012^36^	RCT: open-label, parallel study	Mexico	60	Enalapril (10 mg/day)	Losartan (50 mg/day)	45.8 ± 19.0	24 (40.0)	3.9 ± 1.8^b^	NR	APD	12 months	1/8
Ito et al., 2014^e ^^14^	RCT: open-label, parallel study	Japan	158	Spironolactone (25 mg/day)	Active control^c^	56.5 ± 13.4	45 (28.5)	NR	1009.2 ± 762.2	Not specified	24 months	3/8
Szeto et al., 2015^38^	Non-randomised studies: retrospective cohort study	Hong Kong	645	ACEI/ARB users	Non-ACEI/ARB users	57.2 ± 12.7	286 (44.3)	3.7 ± 2.3^b^	NR	CAPD	66.3 ± 34.7 months	7/9
Yongsiri et al., 2015^25^	RCT: double-blind, crossover study	Thailand	20	Spironolactone (25 mg/day)	Placebo^f^	52.4 ± 12.4	12 (60.0)	NR	895.0 ± 582.0	CAPD	1 months	3/8
Shen et al., 2017^26^	Non-randomised studies: retrospective cohort study	USA	886	ACEI/ARB users	Non-ACEI/ARB users	65.5 ± 13.6	390 (44.0)	8.4 ± 4.8^b^	991.6 ± 648.8	CAPD, CCPD	12.0 ± 10.8 months	8/9

^a^For RCTs, and quasi-experimental study, the risk of bias was assessed based on the Cochrane Collaboration’s tool and expressed as the number of low risk-risk judgments (ranging 0–8), while the Newcastle-Ottawa Scale (NOS) was applied for cohort study and summary scores ranging from 0–9 points.

^b^Adjusted for body surface area.

^c^Trial did not use a placebo.

^d^Data were based on nonanuric and anuric patients at baseline.

^e^All participants in both arm received ACEI or ARB treatment for at least 3 months.

^f^Antihypertensive agents were allowed except for ACEIs or ARBs treatment.

Abbreviations: ACEI, angiotensin-converting enzyme inhibitor; APD, automated peritoneal dialysis; ARB, angiotensin II receptor blocker; CAPD, continuous ambulatory peritoneal dialysis; CCPD, continuous cyclic peritoneal dialysis; rGFR, residual glomerular filtration rate; NR, not reported; PD, peritoneal dialysis; RCTs, randomised-controlled trials; SD, standard deviation; USA, the United States of America.

### Risk of bias of included studies

The summary results of the risk of bias assessment are shown in Table 1. Overall, the included studies had low methodological quality and high risk of bias (Supplementary Table S4). Among 10 RCTs and 3 quasi-experimental included studies, the risk of bias was high or unclear for sequence generation (61.5%); allocation concealment (69.2%); blinding of participants (92.3%), blinding of personnel (92.3%), and blinding of outcomes assessors (92.3%); completeness of outcome reporting (53.8%); and selective reporting of outcomes (92.3%). According to the Newcastle-Ottawa Scale (NOS), the summary scores of included 7 cohort studies ranged from 6–8 points, 5 cohort studies were considered to be at high-quality (≥7 points).

### Residual kidney function

**Figure 3:**
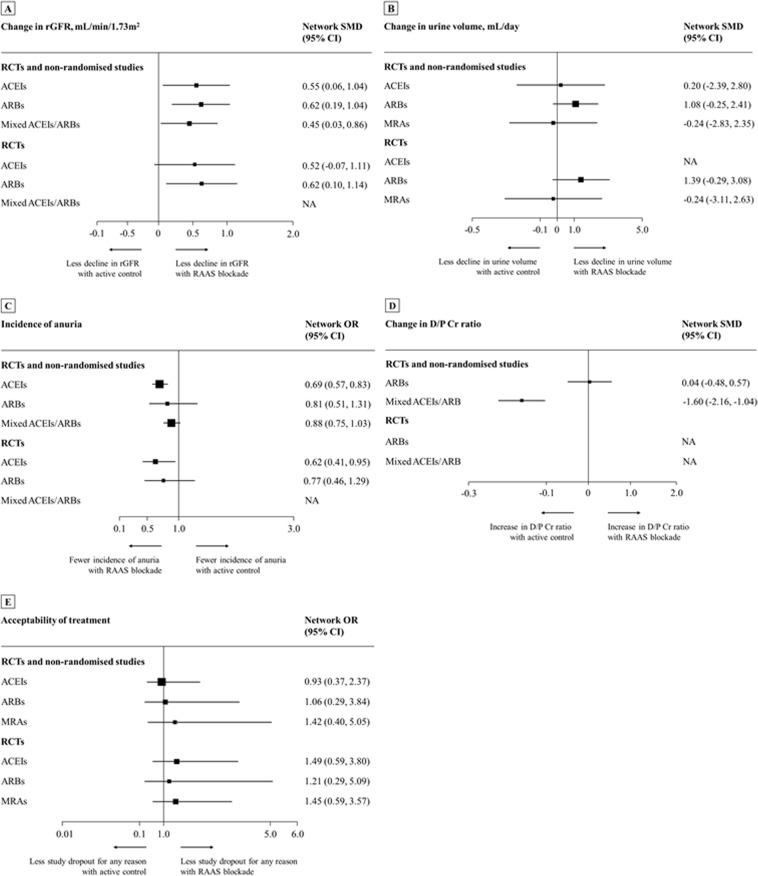
Network meta-analysis of RAAS blockade compared with active control for primary outcomes. Abbreviations: ACEIs, angiotensin-converting enzyme inhibitors; ARBs, angiotensin II receptor blockers; CI, confidence interval; D/P Cr, dialysate-to-plasma creatinine; MRAs, mineralocorticoid receptor antagonists; NA, not applicable; OR, odd ratio; RAAS, renin-angiotensin-aldosterone system blockade; RCTs, randomised-controlled trials; rGFR, residual glomerular filtration rate; SMD, standardised mean difference.

Findings from pairwise meta-analysis and NMA for RKF outcomes were consistent, except for the effect of ACEIs on change in rGFR (Table 2). The overall results are summarised in Fig. 3; and Supplementary, Tables S5 and S6. For pairwise comparison, heterogeneity was low to moderate (*I*^2^ < 75%), however, *I*^2^ values higher than 75% was identified for the comparisons of ARBs versus active control on change in urine volume (*I*^2^ = 87%). Rankogram and cumulative probability curves of RAAS blockade against active control are provided in Supplementary, Figs. S2 and S3, respectively.

**Table 2:** Summary of findings versus active control and the strength of evidence from pairwise meta-analysis and NMA.

Treatment Comparison^a^	Findings from RCTs					Findings from RCTs and non-randomised studies					Strength of Evidence
	No. of Studies Included^b^ (N)	Pairwise Meta-Analysis			Network Meta-Analysis^c^	No. of Studies Included^b^ (N)	Pairwise Meta-Analysis			Network Meta-Analysis^d^	
		Effect Estimate (95% CI)	*I*^2^ (*P*-Value)	*τ*^2^	Effect Estimate (95% CI)		Effect Estimate (95% CI)	*I*^2^ (*P*-Value)	*τ*^2^	Effect Estimate (95% CI)	
**Change in rGFR, mL/min/1.73 m**^**2**^											
ACEIs	2 (62)	0.17 (-0.80 to 1.15)	70% (0.069)	0.353	SMD 0.52 (−0.07 to 1.11)	2 (62)	0.17 (−0.80 to 1.15)	70% (0.069)	0.353	SMD 0.55 (0.06 to 1.04)	Low
ARBs	3 (104)	0.82 (0.17 to 1.47)	59% (0.086)	0.195	SMD 0.62 (0.10 to 1.14)	3 (104)	0.82 (0.17 to 1.47)	59% (0.086)	0.195	SMD 0.62 (0.19 to 1.04)	Low
Mixed ACEIs/ARBs	NA	NA	NA	NA	NA	2 (711)	0.41 (0.25 to 0.57)	0% (0.620)	<0.001	SMD 0.45 (0.03 to 0.86)	Insufficient
**Change in Urine Volume, mL/day**											
ACEIs	NA	NA	NA	NA	NA	1 (43)	SMD 0.20 (−0.45 to 0.86)	NA	NA	SMD 0.20 (−2.39 to 2.80)	Insufficient
ARBs	3 (112)	SMD 1.38 (−0.07 to 2.82)	91% (<0.001)	1.466	SMD 1.39 (−0.29 to 3.08)	4 (120)	SMD 1.07 (−0.07 to 2.21)	87% (<0.001)	1.164	SMD 1.08 (−0.25 to 2.41)	Low
MRAs	1 (20)	SMD −0.24 (−0.86 to 0.39)	NA	NA	SMD −0.24 (−3.11 to 2.63)	1 (20)	SMD −0.24 (−0.86 to 0.39)	NA	NA	SMD −0.24 (−2.83 to 2.35)	Insufficient
**Incidence of Anuria**											
ACEIs	1 (60)	OR 0.58 (0.36 to 0.94)	NA	NA	OR 0.62 (0.41 to 0.95)	4 (1,265)	OR 0.69 (0.57 to 0.83)	0.0% (0.436)	<0.001	OR 0.69 (0.57 to 0.83)	Low
ARBs	2 (76)	OR 0.89 (0.45 to 1.73)	0.0% (0.903)	<0.001	OR 0.77 (0.46 to 1.29)	2 (76)	OR 0.89 (0.45 to 1.73)	0.0% (0.724)	<0.001	OR 0.81 (0.51 to 1.31)	Low
Mixed ACEIs/ARBs	NA	NA	NA	NA	NA	2 (1,338)	OR 0.88 (0.75 to 1.03)	0.0% (0.409)	<0.001	OR 0.88 (0.75 to 1.03)	Insufficient
**Change in D/P Cr Ratio**											
ARBs	NA	NA	NA	NA	NA	2 (28)	SMD 0.04 (−0.48 to 0.57)	0.0% (0.957)	<0.001	SMD 0.04 (−0.48 to 0.57)	Insufficient
Mixed ACEIs/ARBs	NA	NA	NA	NA	NA	1 (66)	SMD −1.60 (−2.16 to −1.04)	NA	NA	SMD −1.60 (−2.16 to −1.04)	Insufficient
**Acceptability of Treatment**											
ACEIs	3 (131)	OR 1.57 (0.52 to 4.71)	26.4% (0.257)	0.266	OR 1.49 (0.59 to 3.80)	4 (185)	OR 0.93 (0.26 to 3.26)	59.0% (0.062)	0.897	OR 0.93 (0.37 to 2.37)	Low
ARBs	3 (116)	OR 1.12 (0.23 to 5.43)	0.0% (0.831)	<0.001	OR 1.21 (0.29 to 5.09)	6 (151)	OR 1.08 (0.29 to 3.99)	0.0% (0.996)	<0.001	OR 1.06 (0.29 to 3.84)	Low
MRAs	2 (178)	OR 1.46 (0.75 to 2.84)	0.0% (0.850)	<0.001	OR 1.45 (0.59 to 3.57)	2 (178)	OR 1.46 (0.75 to 2.84)	0.0% (0.850)	<0.001	OR 1.42 (0.40 to 5.05)	Low

^a^Summary of treatment effects compared with active control.

^b^Number of studies with direct comparison.

^c^The *τ*^2^ values in the network analyses from RCTs were: change in rGFR, 0.153 (moderate heterogeneity); change in urine volume, 2.043 (high heterogeneity); incidence of anuria, < 0.001 (low heterogeneity); change in D/P Cr ratio (NA); acceptability of treatment, 0.104 (moderate heterogeneity).

^d^The *τ*^2^ values in the network analyses from RCTs and non-randomised studies were: change in rGFR, 0.061 (moderate heterogeneity); change in urine volume, 1.647 (high heterogeneity); incidence of anuria, < 0.001 (low heterogeneity); change in D/P Cr ratio (NA); acceptability of treatment, 0.340 (high heterogeneity).

Abbreviations: ACEIs, angiotensin-converting enzyme inhibitors; ARBs, angiotensin II receptor blockers; CI, confidence interval; D/P Cr, dialysate-to-plasma creatinine; MRAs, mineralocorticoid receptor antagonists; NA, not applicable; NMA, network meta-analysis; OR, odds ratio; rGFR, residual glomerular filtration rate; RCTs, randomised-controlled trials; SMD, standardised mean difference.

**Figure 4:**
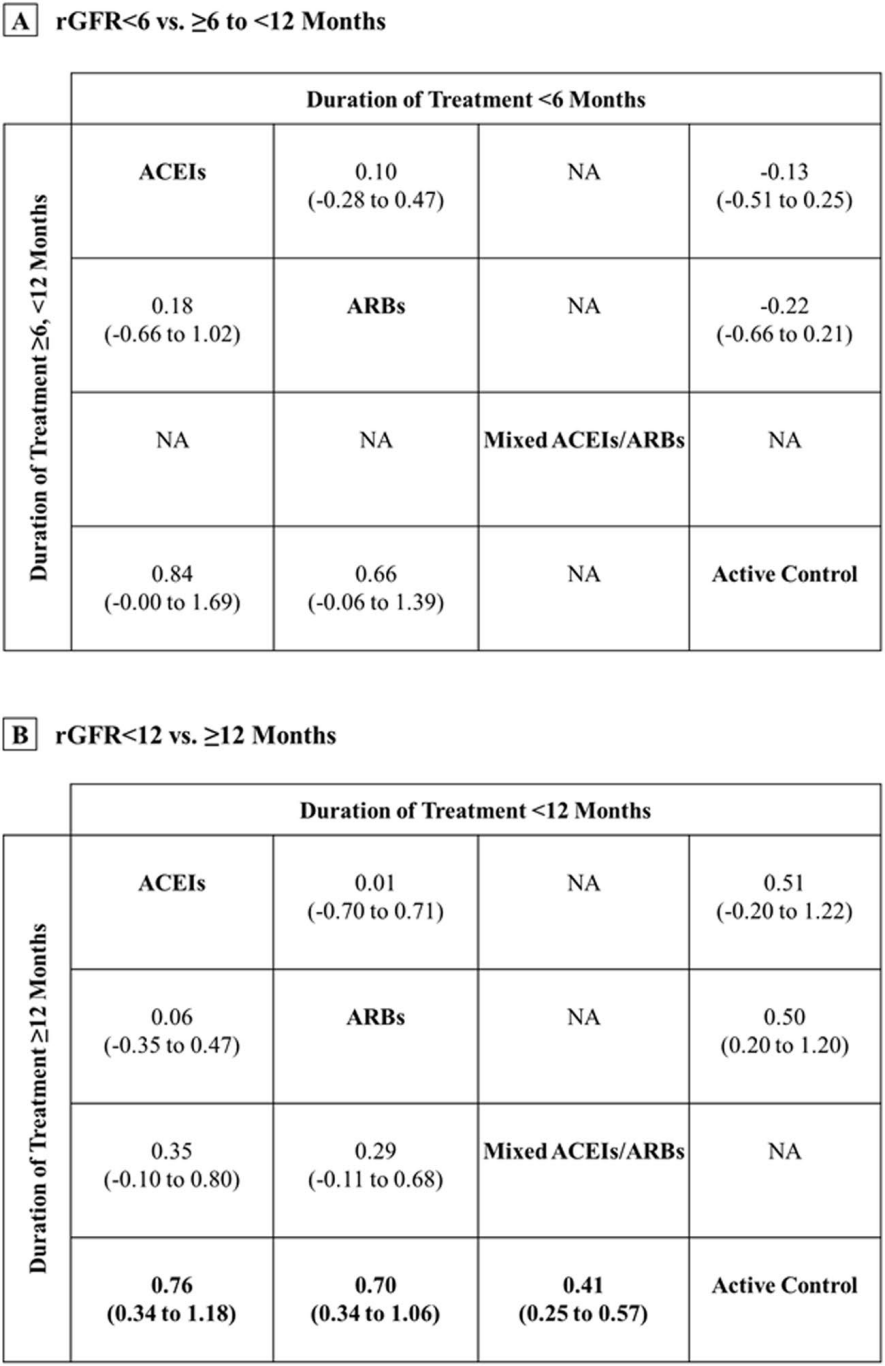
Mean change in rGFR by duration of treatment: evidence from NMA (RCTs and non-randomised studies). Note: Bold values indicate statistical significance. For study duration <6 or <12 months, SMDs >0 indicate that the treatment specified in the row is more efficacious than that in the column. For study duration ≥6 to <12 or ≥12 months, SMDs >0 indicate that the treatment specified in the column is more efficacious than that in the row column. To obtain SMDs for comparisons in the opposite direction, positive values should be converted into negative values, and vice versa. Abbreviations: ACEIs, angiotensin-converting enzyme inhibitors; ARBs, angiotensin II receptor blockers; CIs, confidence intervals; NMA, network meta-analysis; RCTs, randomised-controlled trials; rGFR, residual glomerular filtration rate; SMDs, standardised mean differences.

For change in rGFR, ACEIs or ARBs was substantially more efficacious than active control with medium treatment effects (standardised mean difference [SMD] range 0.45 to 0.55; Table 2 and Fig. 3). Interestingly, the NMA resulted that the protective effect of RAAS blockade on preservation of rGFR was found if duration of treatment was ≥12 months (SMD range 0.41 to 0.76; Fig. 4). No significant difference among treatment comparisons for change in urine volume was observed. Moreover, only ACEIs was associated with a significantly decreased risk of anuria compared with active controls. The odd ratios (ORs) from NMA of RCTs alone and RCTs/non-randomised studies together were 0.62 (95% confidence interval [CI], 0.41 to 0.95) and 0.69 (95% CI, 0.57 to 0.83), respectively (Table 2 and Fig. 3).

Owing to limited data, a dose-response relationship and several preplanned subgroup analyses could not be performed. However, our subgroup analyses showed no effect of RAAS blockade on change in rGFR from baseline among study size ≤ 50 participants or non-Asian countries (Supplementary, Tables S7 and S8). Meanwhile, there was no effect of study size or location on the main findings of change in urine volume and incidence of anuria.

### Peritoneal membrane function and ultrafiltration volume

Overall results of PMF and UF volume did not differ among pairwise meta-analyses (Supplementary, Tables S5 and S6) and NMAs (Supplementary, Tables S9 and S10). Nonetheless, effect estimates were mainly drawn from non-randomised studies, and very few data were available. Thus, it was impossible to establish dose- and duration-response effects or subgroup analyses. Compared with active controls, mixed ACEIs/ARBs were associated with a statistically significant decrease in D/P Cr ratio (SMD −1.60; 95% CI [−2.16 to −1.04]; Table 2 and Fig. 3), whereas increase the 4-hour UF by the peritoneal equilibration test (SMD 1.36; 95% CI [0.02 to 2.70]; Supplementary, Table S9).

### Acceptability of treatment and safety outcomes

Both pairwise meta-analysis and NMA of primary analysis (Table 2 and Fig. 3; Supplementary, Tables S5 and S6) and subgroup analyses (Supplementary, Tables S7 and S8) did not differ between treatment comparisons. Likewise, no associations with the occurrence of adverse events were found (Supplementary, Tables S9 and S10). Nevertheless, only one study^14^ revealed that MRAs with spironolactone increased the risk of gynaecomastia (OR 6.40 [1.37–29.92], Supplementary, Table S10), while no study reported safety outcome on angioedema/oedema or HRQOL.

### Sensitivity analyses

Post-hoc analysis by adding conference abstracts could not be performed due to lack of additional relevant studies. The results for incidence of anuria, daily UF volume, and acceptability of treatment were robust and did not change substantially in sensitivity analyses (Supplementary, Tables S11–S13).

For change in rGFR, there was no protective effect of treatment comparisons after the analysis was restricted to studies from non-mainland China, as well as when studies with mixed ACEIs/ARBs were removed, except for ARBs which was not different when excluding mixed treatment effects (Supplementary, Table S14). Notably, the positive association between ARBs and change in urine volume was found when restricted analysis by excluding studies from mainland China was performed (SMD 3.19 [2.51–3.87], Supplementary, Table S12). Exclusion of crossover studies did not result in substantial differences for change in D/P Cr ratio and 4-hour UF volume, while removal of these studies resulted in ACEIs being associated with a significantly increased risk of dry cough (Supplementary, Table S13).

### Assessment of heterogeneity, inconsistency, transitivity, and publication bias

For NMAs, most outcomes were associated with low to moderate statistically heterogeneity, except for change in urine volume, acceptability of treatment, and dry cough which were associated with high heterogeneity. Network meta-regression analyses could not be performed due to limited data availability. However, univariate meta-regression of pairwise analyses revealed that the heterogeneity of studies was not accounted by any of the study- or baseline participant-level characteristics for change in rGFR, urine volume, incidence of anuria or acceptability of treatment (Supplementary, Table S15).

There was no evidence of inconsistency between direct and indirect evidence based upon findings from the loop-specific and node-splitting methods (Supplementary, Tables S16 and S17), however, inconsistency for change in D/P Cr ratio and 4-hour UF volume were identified by the design-by-treatment interaction approach (Supplementary, Table S18). Analysis of comparison-adjusted funnel plots indicated no evidence of asymmetry, except for change in urine volume (Supplementary, Fig. S4).

### Strength of the body of evidence

**Figure 5:**
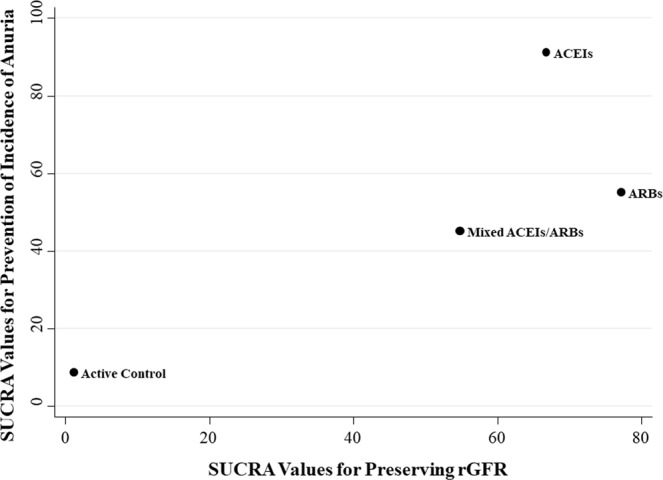
Two-dimension rank plot of effect estimates according to efficacy on preservation of rGFR and incidence of anuria. Abbreviations: ACEIs, angiotensin-converting enzyme inhibitors; ARBs, angiotensin II receptor blockers; rGFR, residual glomerular filtration rate; SUCRA, surface under the cumulative ranking curve.

We graded the strength of evidence of ACEIs or ARBs for the preservation of RKF (rGFR and anuria) as low, whereas several outcomes were graded as insufficient due to study limitations, inconsistency, and imprecision of effect estimates (Table 2). We therefore ranked RAAS blockade with regard to rGFR and incidence of anuria as two dimensions (Fig. 5). The comparative effects of RAAS blockade according to the efficacy on preserve RKF and acceptability of treatment are provided in Supplementary, Fig. S5. However, effects of RAAS blockade on PMF could not be established due to limited evidence. Details of evidence synthesis by the Grading of Recommended Assessment, Development and Evaluation (GRADE) system are described in Supplementary, Table S19.

## Discussion

Our NMA sheds unified hierarchies of evidence for all RAAS inhibitors, only ACEIs and ARBs treatments were efficacious than active control and ranked as the highest level of efficacy for the prevention of anuria and preservation of rGFR, respectively. Effects of RAAS blockade on PMF were inconclusive likely based on few studies examining PMF and its constituents as an outcome. No specific RAAS blockade classes were superior to other treatments with regard to adverse outcomes. However, it should be noted that our findings are limited by the strength of evidence according to the GRADE system revealed low or insufficient quality evidence.

Compared with existing reviews, our study has important methodological differences. This review incorporated of both RCTs and non-randomised studies to address some limitations of RCT, such as small sample size and short follow-up period, allow assessment of treatment comparisons simultaneously, and generalisable evidence^40^. A review by Akbari *et al*.^29^, demonstrated a protective effect of treatment with ACEIs/ARBs on preservation of rGFR at 12 months (mean difference [MD] 0.91 [0.14–1.68] mL/min/1.73 m^2^, and in line Zhang *et al*.^28^, (MD 1.11 [0.38–1.83] mL/min/1.73 m^2^ for treatment with ARBs). Given our findings, the superiority of ACEIs or ARBs is similar to the pattern seen in pairwise meta-analyses. Long-term use (≥12 months) of ACEIs and ARBs shown substantial benefit for change in rGFR (SMD 0.76 [0.34–1.18] and 0.70 [0.34–1.06] mL/min/1.73 m2, respectively), and prove more clinically significant benefit compared with treatment <12 months.

From the methodological viewpoint and based on the natural disease progression, the length of follow-up time is important to provide adequate statistical power. Besides a relatively small number of patients, it can be postulated that the short duration of treatment <12 months was ultimately underpowered to observe the intended effects of the RAAS blockade. Additionally, although the mechanisms of action of the RAAS blockade effects have not been clearly described in PD patients, delayed therapeutic effects of the use of RAAS blockade has been apparently illustrated in the certain population such as diabetic nephropathy and those with dietary protein restriction—presumably because of the hemodynamic effect of the inhibition of the RAAS activation^41–43^. In this circumstance, it may further explain that no significant association of the RAAS blockade on the rGFR was observed when a duration of treatment was <12 months. However, adequate powered controlled trials are warranted to confirm our findings in terms of duration-response effects.

Indeed, the urine volume is variable among the dialysis population, ranging from oliguria to normal or even above normal levels. These results are related to the circumstance that the urine output is influenced not only by the GFR, but also by the difference between the GFR and the rate of tubular transport mechanisms. In the PD population, urine volume accounted for only one half of the variance in GFR (r^2^ = 0.55, *P* < 0.001)^37^. Thereby, we hypothesised that the effect of RAAS blockade on the preserve of urine volume does not always in line with the rGFR. However, it is unclear whether or not the use of RAAS blockade among PD patients may have an additional effect on a slower rate of decline of urine volume. Owing to the statistical power was limited and the imprecision of this measure, including the errors and reliability of urine collection. Moreover, the pooled SMDs in our analyses were calculated using the median, range and/or interquartile range, which could be limited by the sample size of the included studies. Thus, caution must be considered in an interpretation of the findings.

Long-term PD treatment can lead to peritoneum injury by hypertonic PD solutions. Theoretically, RAAS blockade may have important role in “peritoneal protection”, as RAAS may reduce membrane fibrosis and neoangiogenesis, and decreased peritoneal protein loss that occurs over time^16–18,24^. However, we found that the data for these outcomes were substantially inconsistent, which suggest less reliability of the findings. We conclude that the effects of RAAS blockade on peritoneal membrane integrity remain unclear and warrant further clarification. Both pairwise and NMA had similar adverse outcomes across all other treatments, however, we underscore that the results were surrounded by uncertainty of effects estimated.

To our knowledge, this systematic review and NMA consisted of unpublished data, and more comprehensive than previous reviews as we did not impose any language restrictions. Our study was conducted by retrieving evidence from both RCTs and non-randomised studies, which reflect real-world practices.

However, several potential limitations of our study are worth noting. Firstly, even sophisticated adjusted cohort studies may be subjects to unmeasured confounders^40^. Moreover, the limited interconnections in the evidence networks studies should be stated that an indirect estimate is susceptible to confounding. Thus, findings should be interpreted with caution since it does not always agree with respect to direct evidences.

Secondly, heterogeneity of outcomes definition is an important concern because of measurement and definition of endpoint were not standardised and defined poorly across included studies. Given the low rate of reporting, measurement of urine volume as well as the incidence of anuria may have been under-ascertained if patients stopped collecting their urine when anuria is near or reached, particularly in the non-randomised studies. We therefore suggest that future trials with high methodological quality are needed.

Thirdly, the interpretation of our subgroup analyses was limited by small sample size and number of studies. Moreover, details of clinically relevant characteristic such as peritoneal equilibration test status, UF, and history of peritonitis were not fully reported across included studies. As such, several preplanned subgroup analyses cannot be performed to explore treatment effects in different subpopulations.

Fourthly, based on crossover design, carry-over effect may contribute to effect estimates of included studies which could have biased results towards a neutral effect on some outcomes, and against specific treatment comparisons. No further significant association was observed between treatment comparisons and change in D/P Cr ratio and 4-hour UF volume, whereas risk of dry cough substantially increases among ACEI users (OR 17.83 [1.22–261.44], Supplementary, Table S13) after removing the crossover design.

Fifthly, the majority of included studies were conducted in Asian countries and restricted largely to CAPD patients. This might affect our findings by limiting the generalisability to other ethnic/racial groups and individuals PD who treated with other PD modalities.

Lastly, because of primary data for the effects of MRAs and DRIs on RKF and PMF were scant, our review was unable to make any meaning conclusions regarding the comparative effectiveness and safety of these drugs among PD patients.

Our findings provide the evidence to support the use of ACEIs or ARBs among PD patients, which in line with the ISPD guidelines for assessment and management of RKF as a cardiovascular risk factor^30^. Besides cardiovascular benefits, we therefore advise the long-term use (≥12 months) of ACEIs or ARBs as an essential treatment compared with other antihypertensive, and this may improve mortality in these population as well. Thus, it is reasonable to suggest that clinician should prescribe ACEIs or ARBs in adult PD patients with significant RKF.

Currently, no recent controlled trial provides direct comparison between MRAs and ACEIs or ARBs treatment among PD patients, thus future trials with high methodological quality are needed. To understand the complex relationship between peritoneum and the kidney, clarifying the effects of each other is required. Besides systemic RAAS blockade, additional experimental study is required to determine effective strategies to decrease local peritoneal RAAS activation. In addition, dose- and duration response relationship of RAAS blockade for these outcomes also needs further investigation.

In summary, our analysis reveals that ACEIs or ARBs treatment is the most effective strategies for preserving RKF, and has similar clinical adverse outcomes across all other treatments. However, little evidence is available for effects of RAAS blockade on PMF in adults PD patients.

## Methods

This study was performed in accordance with guidelines for comparative effectiveness reviews produced by the Agency for Healthcare Research and Quality^44^, and has been reported in line with guidance from the Preferred Reporting Items for Systematic Reviews and Meta-Analyses (PRISMA) extension statement for NMA (Supplementary, Appendix-I)^45^. The pre-specified protocol was registered in PROSPERO (CRD42018083525). Details of the methodology used for this review are described in Supplementary, eMethods.

### Data sources and search strategy

In brief, we searched electronic databases, including PubMed, Embase, Scopus, Web of Science, CINAHL, the Cochrane Library, and grey literature from inception to May 31, 2019, without language restrictions (search strategies are described in Supplementary, Appendix-II). Two independent reviewers screened eligible titles/abstracts and relevant full-text studies. Any disagreement was resolved through a team discussion.

### Study selection and outcomes

We included both RCTs (parallel and cross-over trials) and non-randomised studies (quasi-experimental and retrospective/prospective cohort studies with a control group) that: (i) included adult participants aged ≥18 years; (ii) addressed the effects of the RAAS blockade with ACEIs, ARBs, DRIs, and MRAs and reported at least one of the outcomes of interest; and (iii) compared RAAS blockade against placebo/active control with follow-up of one week onward. We excluded studies that: (i) were N-of-one, case-control, cross-sectional, case series/case reports, and phase I or II study design; (ii) used a dual ACEIs and ARBs treatment; (iii) compared intraperitoneal administered of intervention/control groups; and (iv) included participants that received both PD and haemodialysis treatments.

The primary outcomes of interest were key parameters on RKF and PMF, including (i) rGFR, on the basis of 24-hour collection and calculated using the valid equation proposed (mL/min or mL/min/1.73 m^2^); (ii) urine volume, using a 24-hour timed urine collection or self-reported (mL/day); (iii) the incidence of anuria (defined according to each study, commonly <200 mL/day); (iv) ratio of D/P Cr by the peritoneal equilibration test; and (v) acceptability of treatment (defined to be study dropout for any reason).

The secondary outcomes of interest were: (i) 4-hour UF volume by the peritoneal equilibration test (mL/4-hour); (ii) daily UF volume (mL/day); (iii) adverse events, including hyperkalaemia (as defined by study authors, commonly >5.5 mmol/L), dry cough, hypotension, dizziness, angioedema/oedema, gynaecomastia, and peritonitis; (iv) HRQOL; and (v) healthcare expenditure (e.g. costs, hospitalisation).

### Data extraction and risk of bias assessment

Two reviewers independently extracted the following data using a standardised extraction form: study- and participant-characteristics, interventions (specific RAAS blockade classes [for studies that cannot be specified the class of RAAS blockade, we reported separately as mixed treatment]), control groups, dose of drug, concomitant medications, and predefined outcomes. Data extraction was cross-checked and any discrepancies were resolved through discussion with a third reviewer. For studies with missing or incomplete data, the study authors were contacted by email for clarification. Two reviewers independently performed a critical appraisal of the risk of bias using the Cochrane risk of bias assessment tool for RCTs/quasi-experimental^46^ and the NOS for cohort studies^47^.

### Data synthesis and analysis

Only full-text articles were considered in the primary analysis. We employed a two-step approach to the performance of both pairwise meta-analysis and NMA. For the first step, only RCTs data were used. Subsequently, data from both RCTs and non-randomised studies were incorporated. For any continuous outcome parameters, the summary results from individual studies were captured as the mean difference in the changes with the formula: ∆value_change_ = value_endpoint_ – value_baseline_, in which SD^2^_change_ = [SD^2^_baseline_ + SD^2^_endpoint_ – (2 × ρ × SD_baseline_ × SD_endpoint_)], where ρ stands for the correlation coefficient. We anticipated ρ value of 0.75 between the baseline and endpoint values and equal variances during the RAAS blockade and control groups. Additionally, an estimated value of 0.56 was considered in a sensitivity analysis as described by Szeto *et al*.^38^. For meaningful interpretation of treatment effects, a mean difference of 0.2, 0.5, and 0.8 are considered to be small, medium, and large, respectively^48^.

A random-effects NMA was performed to compare indirect treatment effects for each outcome using a frequentist model. As a measurement varied across studies, the findings from NMAs were expressed in terms of SMDs for continuous outcomes and ORs for dichotomous outcomes, along with 95% CIs. The surface under the cumulative ranking curve, a relative ranking probability, was also estimated for each RAAS blockade class, and rankograms were also prepared^49^.

We evaluated characteristics of populations and study designs across all included studies to evaluate the assumption^49,50^. The network heterogeneity was assumed as the *τ*^2^ statistics, a between-study variance^51–53^. Assessment for consistency of direct and indirect effects was performed using three different approaches: the loop-specific approach, the node-splitting approach, and the design-by-treatment interaction model^49,50,54^. Comparison-adjusted funnel plots were used to investigate for reporting bias and potential effects of small studies^55^.

Preplanned subgroup analyses and meta-regressions were carried out to address potential sources of heterogeneity, including study sample size (≤50 vs. >50 participants), geographical region (Asian vs. non-Asian countries), age (<65 vs 65 years), sex (as reflected by % female), baseline rGFR, urine volume, D/P Cr, blood pressure, history of diabetes, and PD modality.

A series of sensitivity analyses were performed to assess the robustness of primary findings. These included: (i) assuming the correlation coefficient of 0.56 for estimating the SD_change_^38^; (ii) excluding studies from mainland China to avoid a systematic bias related to the trustworthiness as described elsewhere^56,57^; (iii) excluding crossover studies to avoid carry-over effects^58,59^; (iv) excluding studies which classified as mixed treatment intervention; and (v) post-hoc analysis by adding unpublished conference abstracts.

Statistical significance for all tests was 2-tailed, with *P* < 0.05. All analyses were performed using STATA version 14.0 (StataCorp LP).

### Grading the strength of evidence

To assess the strength of evidence for each outcome, the GRADE approach was performed and classified into insufficient-, low-, moderate-, or high-quality evidence^60,61^. The confidence in estimates could be downgraded or upgraded according to the risk of bias, imprecision, inconsistency, and indirectness. Any disputes were resolved by a third reviewer.

## Supplementary information

Supplementary Information

## Data Availability

All data generated and analysed in this review were drawn from the existing article and reported along with the Supplementary Materials.
