# Supplementary material for: Effectiveness of Renin-Angiotensin-Aldosterone System Blockade on Residual Kidney Function and Peritoneal Membrane Function in Peritoneal Dialysis Patients: A Network Meta-Analysis
Source: Sci Rep. 2019 Dec 20;9:19582. doi: 10.1038/s41598-019-55561-5 (PMC6925258; doi:10.1038/s41598-019-55561-5)
Supplement: Supplementary file 1 — Supplementary Information [file 41598_2019_55561_MOESM1_ESM.pdf]

Supplementary Information

Effectiveness of Renin-Angiotensin-Aldosterone System Blockade on Residual Kidney Function and Peritoneal Membrane Function in Peritoneal Dialysis Patients: A Network Meta-Analysis

Authors:

Sirayut Phatthanasobhon, Surapon Nochaiwong*, Kednapa Thavorn, Kajohnsak Noppakun, Setthapon Panyathong, Yuttitham Suteeka, Brian Hutton, Manish M. Sood, Greg A. Knoll, Chidchanok Ruengorn*

***Correspondence:**

1) Surapon Nochaiwong, PharmD

Pharmacoepidemiology and Statistics Research Center (PESRC)

Department of Pharmaceutical Care, Faculty of Pharmacy, Chiang Mai University, Chiang Mai 50200, Thailand, Phone: 66899973365, Fax: 6653222741, Email: surapon.nochaiwong@gmail.com

or

2) Chidchanok Ruengorn, PhD,

[3]Pharmacoepidemiology and Statistics Research Center (PESRC)

Department of Pharmaceutical Care, Faculty of Pharmacy, Chiang Mai University, Chiang Mai 50200, Thailand, Phone: 66810222932, Fax: 6653222741, Email: chidchanok.r@elearning.cmu.ac.th

Supplementary Online Content

**eMethods**	Details of Materials and Methods	S4
**Table S1**	The PICOTS Format: Study Inclusion/Exclusion Criteria	S9
**Table S2**	Measurement and Definition of Clinical Endpoint of Included Studies in the Systematic Review	S10
**Table S3**	Characteristics of Included Studies: RCTs and Non-Randomised Studies	S12
**Table S4**	Risk of Bias Assessment of Included Studies in the Systematic Review	S15
**Table S5**	Summary Results from Network Meta-Analysis of Primary Outcomes: Finding from RCTs vs RCTs/Non-Randomised Studies	S17
**Table S6**	Pairwise Analysis for Primary Outcomes: Finding from RCTs and Non-Randomised Studies	S22
**Table S7**	Subgroup Analysis of Primary Outcomes: Pairwise Analysis (Finding from RCTs and Non-Randomised Studies)	S24
**Table S8**	Subgroup Analysis of Primary Outcomes: Network Meta-Analysis (Finding from RCTs and Non-Randomised Studies)	S29
**Table S9**	Summary Results from Network Meta-Analysis of Secondary Outcomes: Finding from RCTs vs. RCTs/Non-Randomised Studies	S39
**Table S10**	Pairwise Analysis for Secondary Outcomes: Finding from RCTs and Non-Randomised Studies	S47
**Table S11**	Sensitivity Analysis: Assuming the Correlation Coefficient of 0.56 for Estimating the SD_{Change} for Continuous Outcomes (Finding from RCTs and Non-Randomised Studies)	S50
**Table S12**	Sensitivity Analysis: Excluding Studies from Mainland China (Finding from RCTs and Non-Randomised Studies)	S55
**Table S13**	Sensitivity Analysis: Excluding Crossover Studies (Finding from RCTs and Non-Randomised Studies)	S68
**Table S14**	Sensitivity Analysis: Excluding Studies with Mixed Treatment Intervention (Finding from RCTs and Non-Randomised Studies)	S81

Supplementary Online Content (Continued)

**Table S15**	Meta-Regression for Pairwise Analysis	S84
**Table S16**	Evaluation of Inconsistency: Loop-Specific Approach	S88
**Table S17**	Evaluation of Inconsistency: Node-Splitting Model	S89
**Table S18**	Evaluation of Inconsistency: Design-by-Treatment Interaction Model	S90
**Table S19**	Evaluation of the Strength of the Body of Evidence: Finding from RCTs and Non-Randomised Studies	S91
**Figure S1**	Network Plot for Secondary Outcomes	S98
**Figure S2**	Rankogram for Primary Outcomes	S102
**Figure S3**	SUCRA and Cumulative Probabilities Plots for Primary Outcomes	S105
**Figure S4**	Comparison-Adjusted Funnel Plot for Primary Outcomes	S108
**Figure S5**	Two-Dimension Rank Plot of Effect Estimates	S111
**Appendix-I**	PRISMA NMA Checklist of Items to Include When Reporting Systematic Review Involving a Network Meta-analysis	S112
**Appendix-II**	Systematic Review Search Strategy	S116
**eReferences**		S122

eMethods Details of Materials and Methods

Data Sources and Search Strategy

The search strategy was developed and implemented in collaboration with an experienced medical information specialist. We searched electronic databases, including PubMed, Embase, Scopus, Web of Science, CINAHL, and the Cochrane Library, from inception to May 31, 2019, without language restrictions. A combination of generic names and subject headings/Medical Subject Headings terms including pharmacological class or individual renin-angiotensin aldosterone systems (RAAS) blockade and outcomes were used as keywords (Appendix-II).

Grey literature from Google Scholar and ongoing clinical trial register were browsed for identification of additional suitable articles through May 31, 2019 as follows:

(i) Australia and New Zealand's (ANZCTR) (http://www.anzctr.org.au)

(ii) Brazilian Clinical Trials Registry (ReBec) (http://www.ensaiosclinicos.gov.br)

(iii) Chinese Clinical Trial Registry (ChiCTR) (http://www.chictr.org.cn)

(iv) Clinical Research Information Service (CRiS), Republic of Korea (http://cris.cdc.go.kr)

(v) Clinical Trials Registry - India (CTRI) (http://ctri.nic.in)

(vi) Cuban Public Registry of Clinical Trials(RPCEC) (http://registroclinico.sld.cu)

(vii) EU Clinical Trials Register (EU-CTR) (https://www.clinicaltrialsregister.eu/)

(viii) German Clinical Trials Register (DRKS) (http://www.drks.de)

(ix) Iranian Registry of Clinical Trials (IRCT) (http://www.irct.ir/)

(x) Japan Primary Registries Network (https://rctportal.niph.go.jp/)

(xi) The Netherlands Trial Register (http://www.trialregister.nl)

(xii) Pan African Clinical Trial Registry (PACTR) (http://www.pactr.org/)

(xiii) Peruvian Registry of Clinical Trials (http://www.ins.gob.pe/ensayosclinicos)

(xiv) Philippine Health Research Registry (http://registry.healthresearch.ph/)

(xv) Sri Lanka Clinical Trials Registry (SLCTR) (http://www.slctr.lk/)

(xvi) South African National Clinical Trials Register (http://www.sanctr.gov.za)

(xvii) Swiss FOPH Human Research Projects (https://www.kofam.ch/en/swiss-clinical-trials-portal.html)

(xviii) Tanzania Clinical Trial Registry (http://www.tzctr.or.tz)

(xix) Thai Clinical Trials Registry (http://www.clinicaltrials.in.th)

(xx) The United Kingdoms' ISRCTN registry (http://www.isrctn.com)

(xxi) The US National Institutes of Health Ongoing Trials Registry (http://clinicaltrials.gov)

(xxii) The World Health Organization International Clinical Trials Registry Platform (ICTRP) (https://www.who.int/ictrp)

The abstracts of conference proceedings from the major international nephrology congresses were also searched through May 31, 2019 for any additional relevant studies as follows:

(i) American Society of Nephrology (ASN)

(ii) European Renal Association-European Dialysis and Transplant Association (ERA-EDTA)

(iii) International Society of Nephrology (ISN)

Reference lists of the retrieved studies, relevant guidelines as well as prior systematic reviews were manually sought for other eligible studies.

Study Selection

Eligible titles/abstracts and relevant full-text studies were screened independently by two reviewers (S. Phatthanasobhon and S.N.). Potential eligible studies in non-English language were translated before full-text appraisal. Any disagreement was resolved through a team discussion and/or consultation with the third reviewer (C.R.). The PICOTS Format: study inclusion/exclusion criteria are provided in Supplementary data, Table S1; that included: (i) adult participants aged 18 years or older those on incident and prevalent PD; (ii) the effects of the RAAS blockade with angiotensin-converting enzyme inhibitors (ACEIs), angiotensin II receptor blockers (ARBs), direct renin inhibitors (DRIs), and mineralocorticoid receptor antagonists (MRAs) for any indication addressed at least one of the outcomes of interest; (iii) comparison of intervention group (RAAS blockade) against placebo, active control, or usual care with follow-up of one week onward.

Excluded studies were: (i) N-of-one, case-control, cross-sectional, case series/case reports, and phase I or II study design; (ii) using a dual ACEIs and ARB treatment; (iii) compared intraperitoneal administered of intervention and control groups; and (iv) participants received both peritoneal (PD) and haemodialysis treatments. Overlapping participants, were assembled together as one single study according to the follow-up period, and/or the most relevant information.

Data Extraction and Risk of Bias Assessment

Two reviewers (S. Phatthanasobhon and S.N.) independently extracted data using a standardised approach and predesigned extraction form as following: study characteristics (e.g. author names, trial design, number of participants, and follow-up duration), participants' baseline characteristics (e.g. age, sex, history of diabetes, mean blood pressure, PD modality, residual glomerular filtration rate [rGFR, urine volume, and peritoneal membrane function), intervention (specific RAAS blockade classes [for studies that cannot be specified the class of RAAS blockade, we reported separately as mixed treatment intervention]), specific control groups, dose of drug, and concomitant medications), and predefined outcomes of interest. If a different measurement index (e.g. estimated GFR, renal creatinine clearance, and different type of peritoneal equilibration test [PET] solution) was applied, we extracted data according to one defined by individual authors. The program GetData Graph Digitizer (http://getdata-graph-digitizer.com) was used to extract numerical values from published figures, if the quantitative data were not obtained. Data extraction was cross-checked and reached consensus for any discrepancies through discussion with a third reviewer (C.R.). For studies with missing or incomplete data, the study authors were contacted by email for clarification.

Reviewers, (S. Phatthanasobhon and S.N.), independently critically appraised the risk of bias according to the study design. RCTs and quasi-experimental studies were assessed by the Cochrane Collaboration assessment tool[1]and summarized as the number of low-risk judgments. Meanwhile, the Newcastle-Ottawa Scale (NOS)[2] was applied to assess the risk of bias of cohort studies, in which the higher scores indicate the quality of study (summary score ranging from 0-9).

Grading the Strength of Evidence

To interpret the findings, the Grading of Recommended Assessment, Development and Evaluation (GRADE) approach was performed independently (S. Phatthanasobhon and S.N.) to assess the strength of evidence (SOE) for each outcome.[3] The GRADE approach was applied to rating the SOE of findings. It was classified into insufficient-, low-, moderate-, or high-quality evidence according to the Agency for Healthcare Research and Quality (AHRQ) recommendation.[4] The confidence in estimates could be rated downgrade or upgrade according to the risk of bias, imprecision, inconsistency, and indirectness. If the direct and indirect estimates were coherent, then the higher of their SOE was assigned to summary of findings. Constantly, for incoherence, the summary effect estimates were downgraded. Any disputes were resolved by a third reviewer (C.R).

Data Synthesis and Analysis

We performed two steps approach of pairwise and network meta-analysis. For the first step, only RCTs data were used. In the second step, data from both RCTs and non-randomised studies were integrated to deliver more timely and comprehensive. We divided the duration of treatment into <6 vs. ≥6 to <12 and <12 vs. ≥12 months to establish the evidence of duration-response effects. If possible, individual RAAS blockade use and dosage were considered to explore the relationship of dose-response effects.

For any continuous outcome parameters (rGFR, urine volume, UF volume, and D/P Cr, and health-related quality of life [HRQOL]), if the mean values as well as the standard deviation (SD) values were not provided, we estimated the sample mean and SD from the sample size, median, range and/or interquartile range.[5] The summary results were expressed as the mean difference in the change with the formula: $\Delta value_{change} = value_{endpoint} - value_{baseline}$, in which $SD^2_{change} = [SD^2_{baseline} + SD^2_{endpoint} - (2 \times \rho \times SD_{baseline} \times SD_{endpoint})]$, where ρ stands for the correlation coefficient. We anticipated ρ value of 0.75 between the baseline and endpoint values and equal variances during the RAAS blockade and control groups. However, an estimated value of 0.56 was considered in a sensitivity analysis as described by Szeto et al.[6] For meaningful interpretation of treatment effects, a mean difference of 0.2, 0.5, and 0.8 are considered to be small, medium, and large, respectively.[7] For binary outcomes (acceptability and drug-related adverse events), studies with zero events were handled by a 0.5 cell correction.[8]

On pairwise meta-analysis, we calculated the standardised mean differences (SMDs) for continuous outcomes and odd ratios (ORs) for dichotomous outcomes, along with 95% confidence intervals (CIs) by using DerSimonian-Laird random-effects models.[9] Heterogeneity was assessed using the Q test, with $P< 0.10$. The degree of inconsistency was investigated by the I^2 and tau-squared (τ^2) statistics, in which the heterogeneity was estimated as low (I^2 of 25%, τ^2 of 0.01), moderate (I^2 of 50%, τ^2 of 0.06), and high (I^2 of 75%, τ^2 of 0.16).[10, 11]

Table S1. The PICOTS Format: Study Inclusion/Exclusion Criteria

**Category**	**Criteria for Inclusion**	**Criteria for Exclusion**
Populations	○ Adult participants aged 18 years or older those on incident and prevalent PD	○ Studies recruiting participants who received both PD and HD treatment<br>○ In vitro or animal studies
Interventions	○ RAAS blockade treatment including ACEIs, ARBs, DRIs, and MRAs for any indications which addressed at least one of the outcome of interest	○ Studies that using a combination of ACEIs and ARBs treatment<br>○ Studies that using IP administered of treatment group
Comparators	○ Placebo, active control, or usual care	○ Studies that compared IP administered of control group<br>○ Studies without control groups
Outcomes	Primary outcomes<br>○ RKF including rGFR, urine volume, and incidence of anuria<br>○ Peritoneal membrane function according 4-hour D/P Cr ratio by PET<br>○ Acceptability of treatment (all-cause discontinuation)<br>Secondary outcomes<br>○ 4-hour ultrafiltration and daily ultrafiltration volume<br>○ Drug-related adverse event (e.g. hyperkalaemia, dry cough, hypotension, dizziness, angioedema/oedema, and gynaecomastia)<br>○ Incidence of peritonitis<br>○ Health-related quality of life<br>○ Healthcare expenditure (e.g. costs, hospitalisation)	○ Studies not providing data for calculate the efficacy of primary outcomes<br>○ Studies with follow-up period less than one week
Timing	○ An extensive search strategy from the inception of bibliographic databases forward to assure all published literature was identified	○ No limit timing of start date<br>○ Studies will not be limited by language.
Setting	○ Both RCTs (parallel and crossover trials) and non-randomised studies (quasi-experimental and cohort studies)	○ N-of-one, case-control, cross-sectional, case series/case reports, and phase I or II study design

Abbreviations: ACEIs, angiotensin-converting enzyme inhibitors; ARB, angiotensin II receptor blockers; D/P Cr, dialysate to plasma creatinine ratio; DRI, direct renin inhibitors; HD, haemodialysis; IP, intraperitoneal; MRA, mineralocorticoid receptor antagonist; PD, peritoneal dialysis; PET, peritoneal equilibration test; RAAS, renin-angiotensin aldosterone systems; RCT, randomised controlled trial; rGFR, residual glomerular filtration rate.

Table S2. Measurement and Definition of Clinical Endpoint of Included Studies in the Systematic Review

Author, Year	Outcome Reported	Outcome Measurement		
		rGFR	Peritoneal membrane function	Definition of Anuria
Favazza et al, 1992	RKF (rGFR), Peritoneal membrane function (4-h UF)	24-h urine collection	4-h PET, using 1.36% PD solution	NA
Moist et al, 2000	RKF (anuria)	Questionnaires (collected and reported as a dichotomous variable, less than or greater than 200 mL/day)	NA	Urine volume <200 mL/day
Johnson et al, 2003	RKF (anuria)	24-h urine collection	NA	Complete anuria, had not passed any urine volume for at least 48 hr
Li et al, 2003	RKF (rGFR, anuria), adverse events	24-h urine collection	NA	Total absence of urine volume
Phakdeekitcharoen et al, 2004	RKF (rGFR), adverse events	24-h urine collection	NA	NA
Suzuki et al, 2004	RKF (rGFR, urine), adverse events	24-hour urine collection	NA	NA
Rojas-Campos et al, 2005	Peritoneal membrane function (D/P Cr ratio)	NA	4-h PET, using 2.5% PD solution	NA
Wang et al, 2005	RKF (rGFR, urine)	24-h urine collection	NA	Total absence of urine volume
Furuya et al, 2006	RKF (urine), Peritoneal membrane function (D/P Cr ratio, daily UF)	NR	NR	NA
Jearnsujitwimol et al, 2006	Peritoneal membrane function (4-h UF), adverse events	NA	4-h PET, using 1.5% PD solution	NA

Abbreviations: D/P Cr, dialysate-to-plasma creatinine; NA, not applicable; NR, not reported; PD, peritoneal dialysis; PET, peritoneal equilibration test; rGFR, residual glomerular filtration rate; RKF, residual kidney function; UF, ultrafiltration volume.

Table S2. Measurement and Definition of Clinical Endpoint of Included Studies in the Systematic Review (Continued)

**Author, Year**	**Outcome Reported**	**Outcome Measurement**		
		**rGFR**	**Peritoneal membrane function**	**Definition of Anuria**
Zhong et al, 2007	RKF (rGFR, urine, anuria)	24-h urine collection	NA	Urine <100 mL/day
Wontanatawatot et al, 2009	Peritoneal membrane function (daily UF), adverse events	NA	4-h PET, using 4.25 PD solution	NA
Jing et al, 2010	RKF (rGFR), peritoneal membrane function (4-h UF, D/P Cr ratio)	NR	4-h PET, using 4.25% PD solution	NA
Kolesnyk et al, 2011	RKF (anuria)	NA	NA	Urine <200 mL/day or had GFR of zero (2 consecutive time points)
Basturk et al, 2012	RKF (urine, anuria), peritoneal membrane function (daily UF, 4-h UF), adverse events	24-h urine collection	4-hour PET	NR
Reyes-Marín et al, 2012	RKF (rGFR, anuria), adverse events	24-h urine collection	NA	NA
Ito et al, 2014	adverse events	NA	NR	NA
Szeto et al, 2015	RKF (rGFR)	24-hour urine collection	NA	NA
Yongsiri et al, 2015	RKF (urine), adverse events	24-hour urine collection	NA	NA
Shen et al, 2017	RKF (anuria)	NA	NA	Urine volune <200 mL/day

Abbreviations: D/P Cr, dialysate-to-plasma creatinine; NA, not applicable; NR, not reported; PD, peritoneal dialysis; PET, peritoneal equilibration test; rGFR, residual glomerular filtration rate; RKF, residual kidney function; UF, ultrafiltration volume.

Table S3. Characteristics of Included Studies: RCTs and Non-Randomised Studies

Author, Year	Center	Diabetes	BMI, kg/m^2	Total Kt/V	SBP, mmHg	DBP, mmHg	MAP, mmHg
Favazza et al, 1992	Single center	NR	NR	NR	NR	NR	123.8 ± 4.4
Moist et al, 2000	Multicenter	506 (49.0)	26.2 (5.7)	NR	NR	NR	102.2 ± 12.4
Johnson et al, 2003	Single center	50 (34.2)	NR	NR	138.8 ± 22.0	81.0 ± 14.0	NR
Li et al, 2003	Single center	28 (46.7)	23.4 (3.8)	2.1 ± 0.6	151.2 ± 15.6	83.6 ± 10.9	106.1 ± 10.4
Phakdeekitcharoen et al, 2004[a]	Single center	NR	NR	2.0 ± 0.5	144.5 ± 13.2	85.6 ± 10.7	NR
Suzuki et al, 2004	Single center	11 (32.4)	24.6 (0.8)	NR	165.5 ± 2.5	76.0 ± 3.4	NR
Rojas-Campos et al, 2005	Single center	8 (40.0)	NR	1.8 ± 0.4	141.0 ± 17.0	88.0 ± 11.0	82.0 ± 16.0
Wang et al, 2005	Single center	NR	NR	2.1 ± 0.6	158.5 ± 39.8	102.5 ± 27.7	NR
Furuya et al, 2006	Single center	0 (0.0)	22.5 (2.2)	2.0 ± 0.4	NR	NR	117.0 ± 10.9
Jearnsujitwimol et al, 2006	Single center	3 (42.8)	NR	NR	NR	NR	107.9 ± 6.5
Zhong et al, 2007	Single center	12 (27.3)	NR	2.0 ± 0.2	134.0 ± 14.4	83.0 ± 11.6	NA
Wontanatawatot et al, 2009	Single center	23 (50.0)	NR	2.1 ± 0.6	146.7 ± 20.1	77.1 ± 8.3	100.3 ± 9.6
Jing et al, 2010	Single center	33 (50.0)	NR	NR	NR	NR	NR
Kolesnyk et al, 2011	Multicenter	90 (19.9)	NR	NR	NR	NR	102.0 ± 12.4
Basturk et al, 2012	Single center	7 (16.3)	NR	2.2 ± 0.4	117.8 ± 19.6	70.3 ± 12.1	NR
Reyes-Marín et al, 2012	Single center	26 (43.3)	25 (2.8)	2.0 ± 0.2	132.5 ± 7.9	75.0 ± 7.9	NR
Ito et al, 2014	Single center	62 (39.2)	NR	NR	136.2 ± 16.0	77.5 ± 11.0	NR
Szeto et al, 2015	Single center	277 (42.9)	23.3 (3.4)	2.2 ± 0.6	142.5 ± 17.5	76.7 ± 11.6	NR
Yongsiri et al, 2015	Single center	13 (65)	25.2 (4.2)	2.2 ± 0.8	124.0 ± 19	67.0 ± 7.0	NR
Shen et al, 2017	Multicenter	522 (58.9)	28.6 (6.2)	NR	NR	NR	NR

Data are expressed as the frequency (percentage) of participants or mean ± standard deviation, unless otherwise indicated.

[a]Data were based nonanuric and anuric patients at baseline

Abbreviations: BMI, body mass index; DBP, diastolic blood pressure; MAP, mean arterial pressure; NR, not reported; RCTs, randomised-controlled trials; SBP, systolic blood pressure.

Table S3. Characteristics of Included Studies: RCTs and Non-Randomised Studies (Continued)

Author, Year	CCI	Hg, g/dL	Serum Albumin, g/dL	PET Status			
				Low	Low Average	High Average	High
Favazza et al, 1992	NR	NR	NR	NR	NR	NR	NR
Moist et al, 2000	NR	NR	3.5 ± 0.5	NR	NR	NR	NR
Johnson et al, 2003	NR	NR	NR	4 (2.7)	47 (32.2)	77 (52.7)	18 (12.3)
Li et al, 2003	NR	NR	3.3 ± 0.6	NR	NR	NR	NR
Phakdeekitcharoen et al, 2004[a]	NR	NR	NR	1 (4.8)	11 (52.3)	8 (38.1)	1 (4.8)
Suzuki et al, 2004	NR	8.4 ± 1.2	3.6 ± 0.6	NR	NR	NR	NR
Rojas-Campos et al, 2005	NR	NR	NR	7 (35.0)	5 (25)	6 (30)	2 (10)
Wang et al, 2005	NR	NR	NR	NR	NR	NR	NR
Furuya et al, 2006	NR	9.8 ± 1.0	3.5 ± 0.3	NR	NR	NR	NR
Jearnsujitwimol et al, 2006	NR	NR	3.7 ± 0.2	0 (0.0)	7 (100.0)	0 (0.0)	0 (0.0)
Zhong et al, 2007	NR	8.8 ± 1.4	NR	NR	NR	NR	NR
Wontanatawatot et al, 2009	NR	NR	3.5 ± 0.4	NR	NR	NR	NR
Jing et al, 2010	NR	NR	NR	NR	NR	NR	NR
Kolesnyk et al, 2011	NR	NR	NR	NR	NR	NR	NR
Basturk et al, 2012	NR	NR	3.8 ± 0.3	NR	NR	NR	NR
Reyes-Marín et al, 2012	NR	10.8 ± 1.5	3.9 ± 0.4	NR	NR	NR	NR
Ito et al, 2014	NR	10.4 ± 1.3	3.4 ± 0.5	NR	NR	NR	NR
Szeto et al, 2015	5.6 ± 2.6	9.3 ± 1.5	3.2 ± 0.4	NR	NR	NR	NR
Yongsiri et al, 2015	7.5 ± 2.5	NR	3.7 ± 0.3	3 (15.0)	7 (35.0)	9 (45.0)	1 (5.0)
Shen et al, 2017	NR	10.8 ± 1.4	3.8 ± 0.5	NR	NR	NR	NR

Data are expressed as the frequency (percentage) of participants or mean ± standard deviation, unless otherwise indicated.

[a]Data were based nonanuric and anuric patients at baseline

Abbreviations: CCI, Charlson comorbidity index; Hg, hemoglobin; NR, not reported; PET, peritoneal equilibration test; RCTs, randomised-controlled trials.

Table S3. Characteristics of Included Studies: RCTs and Non-Randomised Studies (Continued)

Author, Year	D/P Cr	D/D0 Glucose	UF, mL/day	PD Solution	Duration of PD, Months	Concomitant Medications		
						CCBs	Beta-Blockers	NSAIDs
Favazza et al, 1992	NR	0.4 ± 0.1	NR	Glucose-based	11 (2-32)[b]	NR	NR	NR
Moist et al, 2000	NR	NR	NR	NR	New PD patients	602 (58.3)	NR	20 (1.9)
Johnson et al, 2003	0.7 ± 0.1	NR	NR	NR	New PD patients	91 (62.3)	NR	NR
Li et al, 2003	0.6 ± 0.1	NR	NR	NR	10.5 ± 9.2	25 (41.7)	25 (41.7)	NR
Phakdeekitcharoen et al, 2004[a]	NR	NR	NR	NR	33.0 ± 27.0	NR	NR	NR
Suzuki et al, 2004	NR	NR	NR	Glucose-based	NR	29 (90.6)	NR	NR
Rojas-Campos et al, 2005	0.6 ± 0.4	0.38 ± 0.3	NR	Glucose-based	15.4 ± 13	NR	NR	NR
Wang et al, 2005	NR	NR	NR	NR	Received CAPD within 3 months	NR	NR	NR
Furuya et al, 2006	0.7 ± 0.2	NR	269.9 ± 200.5	Glucose-based	21.5 (3-46)[b]	NR	NR	NR
Jearnsujitwimol et al, 2006	NR	0.4 ± 0.4	NR	Glucose-based	42.6 ± 11.3	NR	NR	NR
Zhong et al, 2007	NR	NR	NR	Glucose-based	Received CAPD ≥1 month	NR	NR	NR
Wontanatawatot et al, 2009	NR	NR	NR	Glucose-based	7.4 ± 1.6	36 (78.3)	21 (45.6)	NR
Jing et al, 2010	0.7 ± 0.1	0.4 ± 0.1	775 ± 219.9	Glucose-based	NR	NR	NR	NR
Kolesnyk et al, 2011	NR	NR	NR	NR	New PD patients	188 (41.6)	174 (38.5)	NR
Basturk et al, 2012	0.6 ± 0.1	NR	1320 ± 481	Glucose-based	19.8 ± 15.6	NR	NR	NR
Reyes-Marín et al, 2012	NR	NR	1050 ± 255	NR	19.8 ± 6.6	NR	22 (36.7)	NR
Ito et al, 2014	0.7 ± 0.1	NR	NR	Neutral-pH or icodextrin	10.2 ± 14.9	111 (70.2)	38 (24.0)	NR
Szeto et al, 2015	0.6 ± 0.1	NR	NR	NR	New PD patients	362 (56.1)	NR	NR
Yongsiri et al, 2015	NR	NR	NR	NR	23.6 ± 11.0	NR	NR	NR
Shen et al, 2017	NR	NR	NR	NR	New PD patients	478 (54)	550 (62.1)	NR

Data are expressed as the frequency (percentage) of participants or mean ± standard deviation, unless otherwise indicated.
[a]Data were based nonanuric and anuric patients at baseline; [b]Median (range)
Abbreviations: CCBs, calcium channel blockers; D/D0, 4- to 0-hour dialysate glucose ratio; D/P Cr, dialysate-to-plasma creatinine ratio; NR, not reported; NSAIDs, non-steroidal anti-inflammatory drugs; PD, peritoneal dialysis; RCTs, randomised-controlled trials; UF, ultrafiltration.

Table S4. Risk of Bias Assessment of Included Studies in the Systematic Review by the Cochrane Collaboration's Tool: RCTs and Quasi Experimental Study

**Author, Year**	**Sequence Generation**	**Allocation Concealment**	**Blinding**			**Incomplete Outcome Data**	**Selective Outcome Reporting**	**Other Source of Bias**	**No. of Low-Risk**
			**Participants**	**Personnel**	**Outcome Assessors**				
Favazza et al, 1992	Unclear	Unclear	High	High	High	Low	Unclear	High	1/8
Li et al, 2003	Low	Low	High	High	High	Low	Unclear	Unclear	3/8
Phakdeekitcharoen et al, 2004	Unclear	Low	High	High	High	High	Unclear	Unclear	1/8
Suzuki et al, 2004	Low	Low	High	High	High	Unclear	Unclear	Low	3/8
Rojas-Campos et al, 2005	Unclear	Unclear	High	High	High	Low	Unclear	High	1/8
Wang et al, 2005	Low	Unclear	High	High	High	High	Unclear	Unclear	1/8
Furuya et al, 2006	High	High	High	High	High	Low	Unclear	High	1/8
Jearnsujitwimol et al, 2006	High	High	High	High	High	Low	Unclear	High	1/8
Zhong et al, 2007	Low	High	High	High	High	High	Unclear	Unclear	1/8
Wontanatawatot et al, 2009	Low	Unclear	High	High	High	High	Unclear	Unclear	1/8
Reyes-Marín et al, 2012	Unclear	Low	Unclear	Unclear	Unclear	Unclear	Unclear	Unclear	1/8
Ito et al, 2014	Unclear	Unclear	High	High	Low	High	Low	Low	3/8
Yongsiri et al, 2015	Unclear	Unclear	Low	Low	Unclear	Low	Unclear	High	3/8

Abbreviations: RCTs, randomised-controlled trials.

Table S4. Risk of Bias Assessment of Included Studies in the Systematic Review by the NOS: Cohort Study (Continued)

**Author, Year**	**Selection**				**Comparability**		**Outcomes**			**Total NOS**
	**Representativeness**	**Non-Exposed Selection**	**Exposure: Ascertainment**	**Outcomes Not Present at Entry**	**Controls for: Age, baseline rGFR**	**Control for: Additional Factors[a]**	**Assessment**	**Follow-Up Long Enough**	**Adequacy of Follow-Up**	
Moist et al, 2000	*	*	*	*	…	…	*	*	*	7/9
Johnson et al, 2003	*	*	*	*	…	…	*	*	*	7/9
Jing et al, 2010	*	*	*	*	…	…	*	*	…	6/9
Kolesnyk et al, 2011	*	*	*	*	*	*	*	*	…	8/9
Basturk et al, 2012	*	*	*	*	…	…	*	*	…	6/9
Szeto et al, 2015	*	*	*	*	…	…	*	*	*	7/9
Shen et al, 2017	*	*	*	*	*	*	*	*	*	8/9

[a]Study controls for 2 of Additional factors: sex, race, diabetes, blood pressure, cardiovascular disease, congestive heart failure, and concomitant medication (calcium channel blockers, diuretics).

Abbreviations: NOS, the Newcastle-Ottawa Scale; rGFR, residual glomerular filtration rate.

Table S5. Summary Results from Network Meta-Analysis of Primary Outcomes: Finding from RCTs vs RCTs/Non-Randomised Studies

(A) Change in Residual Glomerular Filtration Rate (mL/min/1.73 m^2)

Effect Estimate: SMDs (95% CIs)			
**ACEIs**	-0.10 (-0.68 to 0.49)	NA	0.52 (-0.07 to 1.11)
-0.07 (-0.55 to 0.42)	**ARBs**	NA	**0.62 (0.10 to 1.14)**
0.10 (-0.55 to 0.75)	**1.18 (0.65 to 2.15)**	**Mixed ACEIs/ARBs**	NA
**0.55 (0.06 to 1.04)**	**0.62 (0.19 to 1.04)**	**0.45 (0.03 to 0.86)**	**Active Control**

Note: Network meta-analysis results for outcome from RCTs only (upper triangle: green) and RCTs/non-randomised studies (lower triangle: blue) For RCT only, SMDs >0 or ORs <1 indicate that the treatment specified in the row is more efficacious than that in the column. For RCTs/non-randomised studies results, SMDs >0 or ORs <1 indicate that the treatment specified in the column is more efficacious than that in the row column. Bold underlined results indicate statistical significance. To obtain SMDs for comparisons in the opposite direction, positive values should be converted into negative values, and vice versa. Similarly, reciprocals should be taken to obtain ORs for comparisons in the opposite direction.

Abbreviations: ACEIs, angiotensin-converting enzyme inhibitors; ARBs, angiotensin II receptor blockers; CIs, confidence intervals; NA, not applicable; ORs, odds ratios; RCTs, randomised-controlled trials; SMDs, standardised mean differences.

Table S5. Summary Results from Network Meta-Analysis of Primary Outcomes: Finding from RCTs vs RCTs/Non-Randomised Studies (Continued)
(B) Urine Volume (mL/day)

**Effect Estimate: SMDs (95% CIs)**			
**ACEIs**	NA	NA	NA
-0.88 (-3.79 to 2.04)	**ARBs**	1.63 (-1.70 to 4.96)	1.39 (-0.29 to 3.08)
0.44 (-3.23 to 4.11)	1.32 (-1.59 to 4.23)	**MRAs**	-0.24 (-3.11 to 2.63)
0.20 (-2.39 to 2.80)	1.08 (-0.25 to 2.41)	-0.24 (-2.83 to 2.35)	**Active Control**

Note: Network meta-analysis results for outcome from RCTs only (upper triangle: green) and RCTs/non-randomised studies (lower triangle: blue) For RCT only, SMDs >0 or ORs <1 indicate that the treatment specified in the row is more efficacious than that in the column. For RCTs/non-randomised studies results, SMDs >0 or ORs <1 indicate that the treatment specified in the column is more efficacious than that in the row column. Bold underlined results indicate statistical significance. To obtain SMDs for comparisons in the opposite direction, positive values should be converted into negative values, and vice versa. Similarly, reciprocals should be taken to obtain ORs for comparisons in the opposite direction.

Abbreviations: ACEIs, angiotensin-converting enzyme inhibitors; ARBs, angiotensin II receptor blockers; CIs, confidence intervals; MRAs, mineralocorticoid receptor antagonists; NA, not applicable; ORs, odds ratios; RCTs, randomised-controlled trials; SMDs, standardised mean differences.

Table S5. Summary Results from Network Meta-Analysis of Primary Outcomes: Finding from RCTs vs RCTs/Non-Randomised Studies (Continued)

(C) Incidence of Anuria

**Effect Estimate: ORs (95% CIs)**			
**ACEIs**	0.81 (0.48 to 1.34)	NA	**0.62 (0.41 to 0.95)**
0.85 (0.53 to 1.36)	**ARBs**	NA	0.77 (0.46 to 1.29)
0.78 (0.61 to 1.00)	0.92 (0.56 to 1.52)	**Mixed ACEIs/ARBs**	NA
**0.69 (0.57 to 0.83)**	0.81 (0.51 to 1.31)	0.88 (0.75 to 1.03)	**Active Control**

Note: Network meta-analysis results for outcome from RCTs only (upper triangle: green) and RCTs/non-randomised studies (lower triangle: blue) For RCT only, SMDs >0 or ORs <1 indicate that the treatment specified in the row is more efficacious than that in the column. For RCTs/non-randomised studies results, SMDs >0 or ORs <1 indicate that the treatment specified in the column is more efficacious than that in the row column. Bold underlined results indicate statistical significance. To obtain SMDs for comparisons in the opposite direction, positive values should be converted into negative values, and vice versa. Similarly, reciprocals should be taken to obtain ORs for comparisons in the opposite direction.

Abbreviations: ACEIs, angiotensin-converting enzyme inhibitors; ARBs, angiotensin II receptor blockers; CIs, confidence intervals; NA, not applicable; ORs, odds ratios; RCTs, randomised-controlled trials; SMDs, standardised mean differences.

Table S5. Summary Results from Network Meta-Analysis of Primary Outcomes: Finding from RCTs vs RCTs/Non-Randomised Studies (Continued)

(D) Dialysate-to-Plasma Creatinine Ratio

**Effect Estimate: SMDs (95% CIs)**		
**ARBs**	NA	NA
**1.64 (0.87 to 2.41)**	**Mixed ACEIs/ARBs**	NA
0.04 (-0.48 to 0.57)	**-1.60 (-2.16 to -1.04)**	**Active Control**

Note: Network meta-analysis results for outcome from RCTs only (upper triangle: green) and RCTs/non-randomised studies (lower triangle: blue) For RCT only, SMDs >0 or ORs <1 indicate that the treatment specified in the row is more efficacious than that in the column. For RCTs/non-randomised studies results, SMDs >0 or ORs <1 indicate that the treatment specified in the column is more efficacious than that in the row column. Bold underlined results indicate statistical significance. To obtain SMDs for comparisons in the opposite direction, positive values should be converted into negative values, and vice versa. Similarly, reciprocals should be taken to obtain ORs for comparisons in the opposite direction.

Abbreviations: ACEIs, angiotensin-converting enzyme inhibitors; ARBs, angiotensin II receptor blockers; CIs, confidence intervals; NA, not applicable; ORs, odds ratios; RCTs, randomised-controlled trials; SMDs, standardised mean differences.

Table S5. Summary Results from Network Meta-Analysis of Primary Outcomes: Finding from RCTs vs RCTs/Non-Randomised Studies (Continued)
(E) Acceptability of Treatment

Effect Estimate: ORs (95% CIs)			
**ACEIs**	1.23 (0.25 to 5.97)	1.03 (0.28 to 3.80)	1.49 (0.59 to 3.80)
0.88 (0.20 to 3.86)	**ARBs**	0.83 (0.15 to 4.58)	1.21 (0.29 to 5.09)
0.65 (0.14 to 3.16)	0.92 (0.56 to 1.52)	**MRAs**	1.45 (0.59 to 3.57)
0.93 (0.37 to 2.37)	1.06 (0.29 to 3.84)	1.42 (0.40 to 5.05)	**Active Control**

Note: Network meta-analysis results for outcome from RCTs only (upper triangle: green) and RCTs/non-randomised studies (lower triangle: blue) For RCT only, SMDs >0 or ORs <1 indicate that the treatment specified in the row is more efficacious than that in the column. For RCTs/non-randomised studies results, SMDs >0 or ORs <1 indicate that the treatment specified in the column is more efficacious than that in the row column. Bold underlined results indicate statistical significance. To obtain SMDs for comparisons in the opposite direction, positive values should be converted into negative values, and vice versa. Similarly, reciprocals should be taken to obtain ORs for comparisons in the opposite direction.

Abbreviations: ACEIs, angiotensin-converting enzyme inhibitors; ARBs, angiotensin II receptor blockers; CIs, confidence intervals; MRAs, mineralocorticoid receptor antagonists; NA, not applicable; ORs, odds ratios; RCTs, randomised-controlled trials; SMDs, standardised mean differences.

Table S6. Pairwise Analysis for Primary Outcomes: Finding from RCTs and Non-Randomised Studies

**Treatment Comparison**	**No. of Studies**	**No. of Participants**	**Effect Estimate (95% CI)**	***P* Value**	**Heterogeneity**			
					***Q* Statistic**	***P* Value**	**I^2 (95% CI)**	**τ^2**
**Change in rGFR (mL/min/1.73 m^2)**								
ACEIs vs. active control	2	62	SMD 0.17 (-0.80 to 1.15)	0.727	3.30	0.069	70% (NA)	0.353
ARBs vs. active control	3	104	SMD 0.82 (0.17 to 1.47)	0.013	4.91	0.086	59% (0-88)	0.195
MRAs vs. active control	NA	NA	NA	NA	NA	NA	NA	NA
Mixed ACEIs/ARBs vs. active control	2	711	SMD 0.41 (0.25 to 0.57)	<0.001	0.25	0.620	0% (NA)	<0.001
ACEIs vs. ARBs	2	70	SMD -0.19 (-0.63 to 0.25)	0.400	0.0	0.961	0% (NA)	<0.001
**Change in Urine Volume (mL/day)**								
ACEIs vs. active control	1	43	SMD 0.20 (-0.45 to 0.86)	0.583	NA	NA	NA	NA
ARBs vs. active control	4	120	SMD 1.07 (-0.07 to 2.21)	0.065	23.81	<0.001	87% (70-95)	1.164
MRAs vs. active control	1	20	SMD -0.24 (-0.86 to 0.39)	0.456	NA	NA	NA	NA
Mixed ACEIs/ARBs vs. active control	NA	NA	NA	NA	NA	NA	NA	NA
ACEIs vs. ARBs	NA	NA	NA	NA	NA	NA	NA	NA
**Incidence of Anuria**								
ACEIs vs. active control	4	1,265	OR 0.69 (0.57 to 0.83)	<0.001	2.72	0.436	0% (0-85)	<0.001
ARBs vs. active control	2	76	OR 0.89 (0.45 to 1.73)	0.724	0.01	0.903	0% (NA)	<0.001
MRAs vs. active control	NA	NA	NA	NA	NA	NA	NA	NA
Mixed ACEIs/ARBs vs. active control	2	1,338	OR 0.88 (0.75 to 1.03)	0.115	0.68	0.409	0% (NA)	<0.001
ACEIs vs. ARBs	1	60	OR 1.09 (0.57 to 2.08)	0.794	NA	NA	NA	NA

Abbreviations: ACEIs, angiotensin-converting enzyme inhibitors; ARBs, angiotensin II receptor blockers; CIs, confidence intervals; MRAs, mineralocorticoid receptor antagonists; NA, not applicable; ORs, odds ratios; RCTs, randomised-controlled trials; rGFR, residual glomerular filtration rate; SMDs, standardised mean differences.

Table S6. Pairwise Analysis for Primary Outcomes: Finding from RCTs and Non-Randomised Studies (Continued)

**Treatment Comparison**	**No. of Studies**	**No. of Participants**	**Effect Estimate (95% CI)**	***P* Value**	**Heterogeneity**			
					***Q* Statistic**	***P* Value**	**I^2 (95% CI)**	**τ^2**
**D/P Cr Ratio**								
ACEIs vs. active control	NA	NA	NA	NA	NA	NA	NA	NA
ARBs vs. active control	2	28	SMD 0.04 (-0.48 to 0.57)	0.896	0.0	0.957	0% (NA)	<0.001
MRAs vs. active control	NA	NA	NA	NA	NA	NA	NA	NA
Mixed ACEIs/ARBs vs. active control	1	66	SMD -1.60 (-2.16 to -1.04)	<0.001	NA	NA	NA	NA
ACEIs vs. ARBs	NA	NA	NA	NA	NA	NA	NA	NA
**Acceptability of Treatment**								
ACEIs vs. active control	4	185	OR 0.93 (0.26 to 3.26)	0.906	7.33	0.062	59% (0-86)	0.897
ARBs vs. active control	6	151	OR 1.08 (0.29 to 3.99)	0.909	0.38	0.996	0% (0-75)	<0.001
MRAs vs. active control	2	178	OR 1.46 (0.75 to 2.84)	0.264	0.04	0.850	0% (NA)	<0.001
Mixed ACEIs/ARBs vs. active control	NA	NA	NA	NA	NA	NA	NA	NA
ACEIs vs. ARBs	2	81	OR 1.00 (0.06 16.45)	1.000	0.0	1.000	0.0%	<0.001

Abbreviations: ACEIs, angiotensin-converting enzyme inhibitors; ARBs, angiotensin II receptor blockers; CIs, confidence intervals; D/P Cr, Dialysate-to-plasma creatinine; MRAs, mineralocorticoid receptor antagonists; NA, not applicable; ORs, odds ratios; RCTs, randomised-controlled trials; SMDs, standardised mean differences; UF, ultrafiltration.

Table S7. Subgroup Analysis of Primary Outcomes: Pairwise Analysis (Finding from RCTs and Non-Randomised Studies)

**Subgroup Comparison[a]: rGFR (mL/min/1.73 m^2)**	**No. of Studies**	**No. of Participants**	**Effect Estimate (95% CI)**	***P* Value**	**Heterogeneity**			
					***Q* Statistic**	***P* Value**	**I^2 (95% CI)**	**τ^2**
**Study Size ≤50 Participants**								
ACEIs	1	9	SMD -0.40 (-1.34 to 0.53)	0.398	NA	NA	NA	NA
ARBs	3	104	SMD 0.82 (0.17 to 1.47)	0.013	4.91	0.086	59% (0-88)	0.195
MRAs	NA	NA	NA	NA	NA	NA	NA	NA
Mixed ACEIs/ARBs	NA	NA	NA	NA	NA	NA	NA	NA
**Study Size >50 Participants**								
ACEIs	1	53	SMD 0.60 (0.05 to 1.15)	0.032	NA	NA	NA	NA
ARBs	NA	NA	NA	NA	NA	NA	NA	NA
MRAs	NA	NA	NA	NA	NA	NA	NA	NA
Mixed ACEIs/ARBs	2	711	SMD 0.41 (0.25 to 0.57)	<0.001	0.25	0.620	0% (NA)	NA
**Asian Countries**								
ACEIs	1	53	SMD 0.60 (0.05 to 1.15)	0.032	NA	NA	NA	NA
ARBs	3	104	SMD 2.27 (1.19, 4.34)	0.013	4.91	0.086	59% (0-88)	0.195
MRAs	NA	NA	NA	NA	NA	NA	NA	NA
Mixed ACEIs/ARBs	2	711	SMD 0.41 (0.25 to 0.57)	<0.001	0.25	0.620	0% (NA)	NA
**Non-Asian Countries**								
ACEIs	1	9	SMD -0.40 (-1.34 to 0.53)	0.398	NA	NA	NA	NA
ARBs	NA	NA	NA	NA	NA	NA	NA	NA
MRAs	NA	NA	NA	NA	NA	NA	NA	NA
Mixed ACEIs/ARBs	NA	NA	NA	NA	NA	NA	NA	NA

[a]Summary of treatment effects compared with active control.

Abbreviations: ACEIs, angiotensin-converting enzyme inhibitors; ARBs, angiotensin II receptor blockers; CIs, confidence intervals; MRAs, mineralocorticoid receptor antagonists; NA, not applicable; RCTs, randomised-controlled trials; rGFR, residual glomerular filtration rate; SMDs, standardised mean differences.

Table S7. Subgroup Analysis of Primary Outcomes: Pairwise Analysis (Finding from RCTs and Non-Randomised Studies) (Continued)

**Subgroup Comparison[a]: Urine Volume (mL/day)**	**No. of Studies**	**No. of Participants**	**Effect Estimate (95% CI)**	***P* Value**	**Heterogeneity**			
					***Q* Statistic**	***P* Value**	**I^2 (95% CI)**	**τ^2**
**Study Size ≤50 Participants**								
ACEIs	1	43	SMD 0.20 (-0.45 to 0.86)	0.538	NA	NA	NA	NA
ARBs	4	120	SMD 1.07 (-0.07 to 2.21)	0.065	23.81	<0.001	87% (70-95)	1.164
MRAs	1	20	SMD -0.24 (-0.86 to 0.39)	0.456	NA	NA	NA	NA
Mixed ACEIs/ARBs								
**Study Size >50 Participants**								
ACEIs	NA	NA	NA	NA	NA	NA	NA	NA
ARBs	NA	NA	NA	NA	NA	NA	NA	NA
MRAs	NA	NA	NA	NA	NA	NA	NA	NA
Mixed ACEIs/ARBs	NA	NA	NA	NA	NA	NA	NA	NA
**Asian Countries**								
ACEIs	NA	NA	NA	NA	NA	NA	NA	NA
ARBs	4	120	SMD 1.07 (-0.07 to 2.21)	0.065	23.81	<0.001	87% (70-95)	1.164
MRAs	1	20	SMD -0.24 (-0.86 to 0.39)	0.456	NA	NA	NA	NA
Mixed ACEIs/ARBs	NA	NA	NA	NA	NA	NA	NA	NA
**Non-Asian Countries**								
ACEIs	1	43	SMD 0.20 (-0.45 to 0.86)	0.538	NA	NA	NA	NA
ARBs	NA	NA	NA	NA	NA	NA	NA	NA
MRAs	NA	NA	NA	NA	NA	NA	NA	NA
Mixed ACEIs/ARBs	NA	NA	NA	NA	NA	NA	NA	NA

[a]Summary of treatment effects compared with active control.

Abbreviations: ACEIs, angiotensin-converting enzyme inhibitors; ARBs, angiotensin II receptor blockers; CIs, confidence intervals; MRAs, mineralocorticoid receptor antagonists; NA, not applicable; RCTs, randomised-controlled trials; SMDs, standardised mean differences.

Table S7. Subgroup Analysis of Primary Outcomes: Pairwise Analysis (Finding from RCTs and Non-Randomised Studies) (Continued)

**Subgroup Comparison[a]: Incidence of Anuria**	**No. of Studies**	**No. of Participants**	**Effect Estimate (95% CI)**	***P* Value**	**Heterogeneity**			
					***Q* Statistic**	***P* Value**	**I^2 (95% CI)**	**τ^2**
**Study Size ≤50 Participants**								
ACEIs	1	27	OR 0.29 (0.08 to 1.06)	0.061	NA	NA	NA	NA
ARBs	2	76	OR 0.89 (0.45 to 1.73)	0.724	0.01	0.903	0% (NA)	<0.001
MRAs	NA	NA	NA	NA	NA	NA	NA	NA
Mixed ACEIs/ARBs	NA	NA	NA	NA	NA	NA	NA	NA
**Study Size >50 Participants**								
ACEIs	3	1,238	OR 0.70 (0.58 to 0.84)	<0.001	1.00	0.607	0% (0-90)	<0.001
ARBs	NA	NA	NA	NA	NA	NA	NA	NA
MRAs	NA	NA	NA	NA	NA	NA	NA	NA
Mixed ACEIs/ARBs	2	1,338	OR 0.88 (0.75 to 1.03)	0.115	0.68	0.409	0% (NA)	<0.001
**Asian Countries**								
ACEIs	1	60	OR 0.58 (0.36 to 0.94)	0.026	NA	NA	NA	NA
ARBs	2	76	OR 0.89 (0.45 to 1.73)	0.724	0.01	0.903	0% (NA)	<0.001
MRAs	NA	NA	NA	NA	NA	NA	NA	NA
Mixed ACEIs/ARBs	NA	NA	NA	NA	NA	NA	NA	NA
**Non-Asian Countries**								
ACEIs	3	1,205	OR 0.71 (0.56 to 0.89)	0.003	2.18	0.337	8% (0-90)	0.005
ARBs	NA	NA	NA	NA	NA	NA	NA	NA
MRAs	NA	NA	NA	NA	NA	NA	NA	NA
Mixed ACEIs/ARBs	2	1,338	OR 0.88 (0.75 to 1.03)	0.115	0.68	0.409	0% (NA)	<0.001

[a]Summary of treatment effects compared with active control.

Abbreviations: ACEIs, angiotensin-converting enzyme inhibitors; ARBs, angiotensin II receptor blockers; CIs, confidence intervals; MRAs, mineralocorticoid receptor antagonists; NA, not applicable; ORs, odds ratios; RCTs, randomised-controlled trials.

Table S7. Subgroup Analysis of Primary Outcomes: Pairwise Analysis (Finding from RCTs and Non-Randomised Studies) (Continued)

**Subgroup Comparison[a]: D/P Cr Ratio**	**No. of Studies**	**No. of Participants**	**Effect Estimate (95% CI)**	***P* Value**	**Heterogeneity**			
					***Q* Statistic**	***P* Value**	**I^2 (95% CI)**	**τ^2**
**Study Size ≤50 Participants**								
ACEIs	NA	NA	NA	NA	NA	NA	NA	NA
ARBs	2	28	SMD 0.04 (-0.48 to 0.57)	0.869	0.00	0.957	0% (NA)	<0.001
MRAs	NA	NA	NA	NA	NA	NA	NA	NA
Mixed ACEIs/ARBs	NA	NA	NA	NA	NA	NA	NA	NA
**Study Size >50 Participants**								
ACEIs	NA	NA	NA	NA	NA	NA	NA	NA
ARBs	NA	NA	NA	NA	NA	NA	NA	NA
MRAs	NA	NA	NA	NA	NA	NA	NA	NA
Mixed ACEIs/ARBs	1	66	SMD -1.60 (-2.16 to -1.04)	<0.001	NA	NA	NA	NA
**Asian Countries**								
ACEIs	NA	NA	NA	NA	NA	NA	NA	NA
ARBs	1	8	SMD 0.07 (-0.91 to 1.05)	0.894	NA	NA	NA	NA
MRAs	NA	NA	NA	NA	NA	NA	NA	NA
Mixed ACEIs/ARBs	1	66	SMD -1.60 (-2.16 to -1.04)	<0.001	NA	NA	NA	NA
**Non-Asian Countries**								
ACEIs	NA	NA	NA	NA	NA	NA	NA	NA
ARBs	1	20	SMD 0.04 (-0.58 to 0.65)	0.912	NA	NA	NA	NA
MRAs	NA	NA	NA	NA	NA	NA	NA	NA
Mixed ACEIs/ARBs	NA	NA	NA		NA	NA	NA	NA

[a]Summary of treatment effects compared with active control.

Abbreviations: ACEIs, angiotensin-converting enzyme inhibitors; ARBs, angiotensin II receptor blockers; CIs, confidence intervals; D/P Cr, dialysate-to-plasma creatinine; MRAs, mineralocorticoid receptor antagonists; NA, not applicable; RCTs, randomised-controlled trials; SMDs, standardised mean differences.

Table S7. Subgroup Analysis of Primary Outcomes: Pairwise Analysis (Finding from RCTs and Non-Randomised Studies) (Continued)

**Subgroup Comparison[a]: Acceptability of Treatment**	**No. of Studies**	**No. of Participants**	**Effect Estimate (95% CI)**	***P* Value**	**Heterogeneity**			
					***Q* Statistic**	***P* Value**	**I^2 (95% CI)**	**τ^2**
**Study Size ≤50 Participants**								
ACEIs	3	125	OR 0.54 (0.23 to 1.26)	0.155	1.93	0.381	0% (0-90)	<0.001
ARBs	6	151	OR 1.08 (0.29 to 3.99)	0.909	0.38	0.996	0 (0-75)	<0.001
MRAs	1	20	OR 1.00 (0.02 to 52.98)	1.000	NA	NA	NA	NA
Mixed ACEIs/ARBs	NA	NA	NA	NA	NA	NA	NA	NA
**Study Size >50 Participants**								
ACEIs	1	60	OR 3.86 (0.93 to 16.05)	0.063	NA	NA	NA	NA
ARBs	NA	NA	NA	NA	NA	NA	NA	NA
MRAs	1	158	OR 1.48 (0.75 to 2.90)	0.258	NA	NA	NA	NA
Mixed ACEIs/ARBs	NA	NA	NA	NA	NA	NA	NA	NA
**Asian Countries**								
ACEIs	2	122	OR 1.69 (0.39 to 7.41)	0.485	2.68	0.102	63% (NA)	0.716
ARBs	5	131	OR 1.09 (0.27 to 4.35)	1.000	0.38	0.984	0% (0-79)	<0.001
MRAs	2	178	OR 1.46 (0.75 to 2.84)	0.264	0.04	0.850	0% (NA)	<0.001
Mixed ACEIs/ARBs	NA	NA	NA	NA	NA	NA	NA	NA
**Non-Asian Countries**								
ACEIs	2	63	OR 0.29 (0.08 to 1.07)	0.063	0.41	0.521	0% (NA)	<0.001
ARBs	1	20	OR 1.00 (0.02 to 52.98)	0.904	NA	NA	NA	NA
MRAs	NA	NA	NA	NA	NA	NA	NA	NA
Mixed ACEIs/ARBs	NA	NA	NA	NA	NA	NA	NA	NA

[a]Summary of treatment effects compared with active control.

Abbreviations: ACEIs, angiotensin-converting enzyme inhibitors; ARBs, angiotensin II receptor blockers; CIs, confidence intervals; MRAs, mineralocorticoid receptor antagonists; NA, not applicable; ORs, odds ratios; RCTs, randomised-controlled trials.

Table S8. Subgroup Analysis of Primary Outcomes: Network Meta-Analysis (Finding from RCTs and Non-Randomised Studies)
(1A) Residual Glomerular Filtration Rate (mL/min/1.73 m^2): Study Size (≤50 vs. >50 Participant)

Effect Estimate: SMDs (95% CIs)			
**ACEIs**	-0.43 (-1.48 to 0.63)	NA	0.23 (-0.83 to 1.29)
0.19 (-0.31 to 0.70)	**ARBs**	NA	0.65 (-0.05 to 1.35)
0.19 (-0.38 to 0.76)	-0.01 (-0.77 to 0.76)	**Mixed ACEIs/ARBs**	NA
**0.60 (0.05 to 1.15)**	0.41 (-0.34 to 1.16)	**0.41 (0.25 to 0.57)**	**Active Control**

Note: NMA for study size ≤50 participants (upper triangle: green) and >50 participants (lower triangle: blue) results for outcome. For study size ≤50 participants, SMDs >0 or ORs <1 indicate that the treatment specified in the row is more efficacious than that in the column. For study size >50 participants, SMDs >0 or ORs <1 indicate that the treatment specified in the column is more efficacious than that in the row column. Bold underlined results indicate statistical significance. To obtain SMDs for comparisons in the opposite direction, positive values should be converted into negative values, and vice versa. Similarly, reciprocals should be taken to obtain ORs for comparisons in the opposite direction.

Abbreviations: ACEIs, angiotensin-converting enzyme inhibitors; ARBs, angiotensin II receptor blockers; CIs, confidence intervals; NMA, network meta-analysis; RCTs, randomised-controlled trials; SMDs, standardised mean differences.

Table S8. Subgroup Analysis of Primary Outcomes: Network Meta-Analysis (Finding from RCTs and Non-Randomised Studies) (Continued)

(2A) Residual Glomerular Filtration Rate (mL/min/1.73 m^2): Asian vs. Non-Asian Countries

Effect Estimate: SMDs (95% CIs)			
**ACEIs**	-0.05 (-0.59 to 0.49)	0.28 (-0.23 to 0.78)	**0.69 (0.21 to 1.17)**
0.19 (-0.31 to 0.70)	**ARBs**	0.33 (-0.09 to 0.74)	**0.74 (0.36 to 1.12)**
NA	NA	**Mixed ACEIs/ARBs**	**0.41 (0.25 to 0.57)**
-0.40 (-1.34 to 0.53)	-0.60 (-1.66 to 0.47)	NA	**Active Control**

Note: NMA for Asian countries (upper triangle: green) and Non-Asian countries (lower triangle: blue) results for outcome. For Asian rcountries, SMDs >0 or ORs <1 indicate that the treatment specified in the row is more efficacious than that in the column. For Non-Asian countries, SMDs >0 or ORs <1 indicate that the treatment specified in the column is more efficacious than that in the row column. Bold underlined results indicate statistical significance. To obtain SMDs for comparisons in the opposite direction, positive values should be converted into negative values, and vice versa. Similarly, reciprocals should be taken to obtain ORs for comparisons in the opposite direction.

Abbreviations: ACEIs, angiotensin-converting enzyme inhibitors; ARBs, angiotensin II receptor blockers; CIs, confidence intervals; NMA, network meta-analysis; RCTs, randomised-controlled trials; SMDs, standardised mean differences.

Table S8. Subgroup Analysis of Primary Outcomes: Network Meta-Analysis (Finding from RCTs and Non-Randomised Studies) (Continued)

(1B) Urine Volume (mL/day): Study Size (≤50 vs. >50 Participant)

**Effect Estimate: SMDs (95% CIs)**			
**ACEIs**	-0.88 (-3.79 to 2.04)	0.44 (-3.23 to 4.11)	0.20 (-2.39 to 2.80)
NA	**ARBs**	1.32 (-1.59 to 4.23)	1.08 (-0.25 to 2.41)
NA	NA	**MRAs**	-0.24 (-2.83 to 2.35)
NA	NA	NA	**Active Control**

Note: NMA for study size ≤50 participants (upper triangle: green) and >50 participants (lower triangle: blue) results for outcome. For study size ≤50 participants, SMDs >0 or ORs <1 indicate that the treatment specified in the row is more efficacious than that in the column. For study size >50 participants, SMDs >0 or ORs <1 indicate that the treatment specified in the column is more efficacious than that in the row column. Bold underlined results indicate statistical significance. To obtain SMDs for comparisons in the opposite direction, positive values should be converted into negative values, and vice versa. Similarly, reciprocals should be taken to obtain ORs for comparisons in the opposite direction.

Abbreviations: ACEIs, angiotensin-converting enzyme inhibitors; ARBs, angiotensin II receptor blockers; CIs, confidence intervals; MRAs, mineralocorticoid receptor antagonists; NMA, network meta-analysis; RCTs, randomised-controlled trials; SMDs, standardised mean differences.

Table S8. Subgroup Analysis of Primary Outcomes: Network Meta-Analysis (Finding from RCTs and Non-Randomised Studies) (Continued)
(2B) Urine Volume (mL/day): Asian vs. Non-Asian Countries

Effect Estimate: SMDs (95% CIs)			
**ACEIs**	NA	NA	NA
NA	**ARBs**	1.32 (-1.59 to 4.23)	1.08 (-0.25 to 2.41)
NA	NA	**MRAs**	-0.24 (-2.83 to 2.35)
-0.20 (-2.27 to 1.86)	NA	NA	**Active Control**

Note: NMA for Asian countries (upper triangle: green) and Non-Asian countries (lower triangle: blue) results for outcome. For Asian countries, SMDs >0 or ORs <1 indicate that the treatment specified in the row is more efficacious than that in the column. For Non-Asian countries, SMDs >0 or ORs <1 indicate that the treatment specified in the column is more efficacious than that in the row column. Bold underlined results indicate statistical significance. To obtain SMDs for comparisons in the opposite direction, positive values should be converted into negative values, and vice versa. Similarly, reciprocals should be taken to obtain ORs for comparisons in the opposite direction.

Abbreviations: ACEIs, angiotensin-converting enzyme inhibitors; ARBs, angiotensin II receptor blockers; CIs, confidence intervals; MRAs, mineralocorticoid receptor antagonists; NMA, network meta-analysis; RCTs, randomised-controlled trials; SMDs, standardised mean differences.

Table S8. Subgroup Analysis of Primary Outcomes: Network Meta-Analysis (Finding from RCTs and Non-Randomised Studies) (Continued)
(1C) Incidence of Anuria: Study Size (≤50 vs. >50 Participant)

Effect Estimate: ORs (95% CIs)			
**ACEIs**	0.33 (0.08 to 1.41)	NA	0.29 (0.08 to 1.06)
0.92 (0.48 to 1.75)	**ARBs**	NA	0.89 (0.45 to 1.73)
0.79 (0.62 to 1.01)	0.86 (0.43 to 1.72)	**Mixed ACEIs/ARBs**	NA
**0.70 (0.58 to 0.84)**	0.76 (0.39 to 1.49)	0.88 (0.75 to 1.03)	**Active Control**

Note: NMA for study size ≤50 participants (upper triangle: green) and >50 participants (lower triangle: blue) results for outcome. For study size ≤50 participants, SMDs >0 or ORs <1 indicate that the treatment specified in the row is more efficacious than that in the column. For study size >50 participants, SMDs >0 or ORs <1 indicate that the treatment specified in the column is more efficacious than that in the row column. Bold underlined results indicate statistical significance. To obtain SMDs for comparisons in the opposite direction, positive values should be converted into negative values, and vice versa. Similarly, reciprocals should be taken to obtain ORs for comparisons in the opposite direction.

Abbreviations: ACEIs, angiotensin-converting enzyme inhibitors; ARBs, angiotensin II receptor blockers; CIs, confidence intervals; NMA, network meta-analysis; RCTs, randomised-controlled trials; SMDs, standardised mean differences.

Table S8. Subgroup Analysis of Primary Outcomes: Network Meta-Analysis (Finding from RCTs and Non-Randomised Studies) (Continued)
(2C) Incidence of Anuria: Asian vs. Non-Asian Countries

Effect Estimate: ORs (95% CIs)			
**ACEIs**	0.65 (0.29 to 1.49)	NA	**0.58 (0.36 to 0.94)**
0.92 (0.48 to 1.75)	**ARBs**	NA	0.89 (0.45 to 1.73)
0.80 (0.62 to 1.03)	0.87 (0.44 to 1.75)	**Mixed ACEIs/ARBs**	NA
**0.71 (0.58 to 0.86)**	0.77 (0.39 to 1.52)	0.88 (0.75 to 1.03)	**Active Control**

Note: NMA for Asian countries (upper triangle: green) and Non-Asian countries (lower triangle: blue) results for outcome. For Asian countries, SMDs >0 or ORs <1 indicate that the treatment specified in the row is more efficacious than that in the column. For Non-Asian countries, SMDs >0 or ORs <1 indicate that the treatment specified in the column is more efficacious than that in the row column. Bold underlined results indicate statistical significance. To obtain SMDs for comparisons in the opposite direction, positive values should be converted into negative values, and vice versa. Similarly, reciprocals should be taken to obtain ORs for comparisons in the opposite direction.

Abbreviations: ACEIs, angiotensin-converting enzyme inhibitors; ARBs, angiotensin II receptor blockers; CIs, confidence intervals; NMA, network meta-analysis; RCTs, randomised-controlled trials; SMDs, standardised mean differences.

Table S8. Subgroup Analysis of Primary Outcomes: Network Meta-Analysis (Finding from RCTs and Non-Randomised Studies) (Continued)

(1D) Dialysate-to-Plasma Creatinine Ratio: Study Size (≤50 vs. >50 Participant)

Effect Estimate: SMDs (95% CIs)		
**ARBs**	NA	0.04 (-0.48 to 0.57)
NA	**Mixed ACEIs/ARBs**	NA
NA	-1.60 (-3.64 to 0.44)	**Active Control**

Note: NMA for study size ≤50 participants (upper triangle: green) and >50 participants (lower triangle: blue) results for outcome. For study size ≤50 participants, SMDs >0 or ORs <1 indicate that the treatment specified in the row is more efficacious than that in the column. For study size >50 participants, SMDs >0 or ORs <1 indicate that the treatment specified in the column is more efficacious than that in the row column. Bold underlined results indicate statistical significance. To obtain SMDs for comparisons in the opposite direction, positive values should be converted into negative values, and vice versa. Similarly, reciprocals should be taken to obtain ORs for comparisons in the opposite direction.

Abbreviations: ACEIs, angiotensin-converting enzyme inhibitors; ARBs, angiotensin II receptor blockers; CIs, confidence intervals; NMA, network meta-analysis; RCTs, randomised-controlled trials; SMDs, standardised mean differences.

Table S8. Subgroup Analysis of Primary Outcomes: Network Meta-Analysis (Finding from RCTs and Non-Randomised Studies) (Continued)
(2D) Dialysate-to-Plasma Creatinine Ratio: Asian vs. Non-Asian Countries

**Effect Estimate: SMDs (95% CIs)**		
**ARBs**	1.66 (-0.71 to 4.04)	0.07 (-1.72 to 1.85)
NA	**Mixed ACEIs/ARBs**	**-1.60 (-3.17 -0.03)**
0.04 (-2.43 to 2.50)	NA	**Active Control**

Note: NMA for Asian countries (upper triangle: green) and Non-Asian countries (lower triangle: blue) results for outcome. For Asian countries, SMDs >0 or ORs <1 indicate that the treatment specified in the row is more efficacious than that in the column. For Non-Asian countries, SMDs >0 or ORs <1 indicate that the treatment specified in the column is more efficacious than that in the row column. Bold underlined results indicate statistical significance. To obtain SMDs for comparisons in the opposite direction, positive values should be converted into negative values, and vice versa. Similarly, reciprocals should be taken to obtain ORs for comparisons in the opposite direction.

Abbreviations: ACEIs, angiotensin-converting enzyme inhibitors; ARBs, angiotensin II receptor blockers; CIs, confidence intervals; NMA, network meta-analysis; RCTs, randomised-controlled trials; SMDs, standardised mean differences.

Table S8. Subgroup Analysis of Primary Outcomes: Network Meta-Analysis (Finding from RCTs and Non-Randomised Studies) (Continued)

(1E) Acceptability of Treatment: Study Size (≤50 vs. >50 Participant)

Effect Estimate: ORs (95% CIs)			
**ACEIs**	0.55 (0.13 to 2.34)	0.55 (0.01 to 32.03)	0.55 (0.24 to 1.28)
1.00 (0.02 to 52.09)	**ARBs**	1.01 (0.02 to 64.79)	1.01 (0.29 to 3.51)
2.61 (0.54 to 12.65)	2.61 (0.04 to 184.25)	**MRAs**	1.00 (0.02 to 52.98)
3.86 (0.93 to 16.05)	3.86 (0.06 to 257.77)	1.48 (0.75 to 2.90)	**Active Control**

Note: NMA for study size ≤50 participants (upper triangle: green) and >50 participants (lower triangle: blue) results for outcome. For study size ≤50 participants, SMDs >0 or ORs <1 indicate that the treatment specified in the row is more efficacious than that in the column. For study size >50 participants, SMDs >0 or ORs <1 indicate that the treatment specified in the column is more efficacious than that in the row column. Bold underlined results indicate statistical significance. To obtain SMDs for comparisons in the opposite direction, positive values should be converted into negative values, and vice versa. Similarly, reciprocals should be taken to obtain ORs for comparisons in the opposite direction.

Abbreviations: ACEIs, angiotensin-converting enzyme inhibitors; ARBs, angiotensin II receptor blockers; CIs, confidence intervals; MRAs, mineralocorticoid receptor antagonists; NMA, network meta-analysis; RCTs, randomised-controlled trials; SMDs, standardised mean differences.

Table S8. Subgroup Analysis of Primary Outcomes: Network Meta-Analysis (Finding from RCTs and Non-Randomised Studies) (Continued)
(2E) Acceptability of Treatment: Asian vs. Non-Asian Countries

Effect Estimate: ORs (95% CIs)			
**ACEIs**	1.35 (0.27 to 6.76)	1.07 (0.27 to 4.21)	1.54 (0.57 to 4.18)
0.56 (0.03 to 9.82)	**ARBs**	0.79 (0.15 to 4.08)	1.14 (0.29 to 4.41)
NA	NA	**MRAs**	1.45 (0.57 to 3.66)
0.31 (0.09 to 1.10)	0.55 (0.03 to 9.78)	NA	**Active Control**

Note: NMA for Asian countries (upper triangle: green) and Non-Asian countries (lower triangle: blue) results for outcome. For Asian countries, SMDs >0 or ORs <1 indicate that the treatment specified in the row is more efficacious than that in the column. For Non-Asian countries, SMDs >0 or ORs <1 indicate that the treatment specified in the column is more efficacious than that in the row column. Bold underlined results indicate statistical significance. To obtain SMDs for comparisons in the opposite direction, positive values should be converted into negative values, and vice versa. Similarly, reciprocals should be taken to obtain ORs for comparisons in the opposite direction.

Abbreviations: ACEIs, angiotensin-converting enzyme inhibitors; ARBs, angiotensin II receptor blockers; CIs, confidence intervals; MRAs, mineralocorticoid receptor antagonists; NMA, network meta-analysis; RCTs, randomised-controlled trials; SMDs, standardised mean differences.

Table S9. Summary Results from Network Meta-Analysis of Secondary Outcomes: Finding from RCTs vs. RCTs/Non-Randomised Studies

(A) 4-Hour Ultrafiltration Volume by Peritoneal Equilibration Test (mL/4 hour)

Effect Estimate: SMDs (95% CIs)			
**ACEIs**	NA	NA	NA
-0.16 (-2.09 to 1.76)	**ARBs**	NA	NA
**-1.72 (-3.42 to -0.02)**	-1.56 (-3.65 to 0.54)	**Mixed ACEIs/ARBs**	NA
-0.36 (-1.41 to 0.70)	-0.19 (-1.81 to 1.42)	**1.36 (0.02 to 2.70)**	**Active Control**

Note: Network meta-analysis results for outcome from RCTs only (upper triangle: green) and RCTs/non-randomised studies (lower triangle: blue) For RCT only, SMDs >0 or ORs <1 indicate that the treatment specified in the row is more efficacious than that in the column. For RCTs/non-randomised studies results, SMDs >0 or ORs <1 indicate that the treatment specified in the column is more efficacious than that in the row column. Bold underlined results indicate statistical significance. To obtain SMDs for comparisons in the opposite direction, positive values should be converted into negative values, and vice versa. Similarly, reciprocals should be taken to obtain ORs for comparisons in the opposite direction.

Abbreviations: ACEIs, angiotensin-converting enzyme inhibitors; ARBs, angiotensin II receptor blockers; CIs, confidence intervals; NA, not applicable; ORs, odds ratios; RCTs, randomised-controlled trials; SMDs, standardised mean differences.

Table S9. Summary Results from Network Meta-Analysis of Secondary Outcomes: Finding from RCTs vs. RCTs/Non-Randomised Studies (Continued)

(B) Daily Ultrafiltration Volume (mL/day)

**Effect Estimate: SMDs (95% CIs)**		
**ACEIs**	NA	NA
1.08 (-0.95 to 3.12)	**ARBs**	NA
0.74 (-0.35 to 1.83)	-0.34 (-2.06 to 1.38)	**Active Control**

Note: Network meta-analysis results for outcome from RCTs only (upper triangle: green) and RCTs/non-randomised studies (lower triangle: blue) For RCT only, SMDs >0 or ORs <1 indicate that the treatment specified in the row is more efficacious than that in the column. For RCTs/non-randomised studies results, SMDs >0 or ORs <1 indicate that the treatment specified in the column is more efficacious than that in the row column. Bold underlined results indicate statistical significance. To obtain SMDs for comparisons in the opposite direction, positive values should be converted into negative values, and vice versa. Similarly, reciprocals should be taken to obtain ORs for comparisons in the opposite direction.

Abbreviations: ACEIs, angiotensin-converting enzyme inhibitors; ARBs, angiotensin II receptor blockers; CIs, confidence intervals; NA, not applicable; ORs, odds ratios; RCTs, randomised-controlled trials; SMDs, standardised mean differences.

Table S9. Summary Results from Network Meta-Analysis of Secondary Outcomes: Finding from RCTs vs. RCTs/Non-Randomised Studies (Continued)
(C) Hyperkalaemia

Effect Estimate: ORs (95% CIs)			
**ACEIs**	0.83 (0.25 to 2.78)	1.11 (0.05 to 26.27)	2.30 (0.20 to 27.06)
0.86 (0.27 to 2.77)	**ARBs**	1.34 (0.05 to 39.75)	2.78 (0.18 to 43.36)
0.62 (0.04 to 9.56)	0.21 (0.03 to 1.72)	**MRAs**	2.07 (0.29 to 15.03)
1.29 (0.20 to 8.44)	1.50 (0.19 to 12.04)	2.07 (0.29 to 15.03)	**Active Control**

Note: Network meta-analysis results for outcome from RCTs only (upper triangle: green) and RCTs/non-randomised studies (lower triangle: blue) For RCT only, SMDs >0 or ORs <1 indicate that the treatment specified in the row is more efficacious than that in the column. For RCTs/non-randomised studies results, SMDs >0 or ORs <1 indicate that the treatment specified in the column is more efficacious than that in the row column. Bold underlined results indicate statistical significance. To obtain SMDs for comparisons in the opposite direction, positive values should be converted into negative values, and vice versa. Similarly, reciprocals should be taken to obtain ORs for comparisons in the opposite direction.

Abbreviations: ACEIs, angiotensin-converting enzyme inhibitors; ARBs, angiotensin II receptor blockers; CIs, confidence intervals; MRAs, mineralocorticoid receptor antagonists; NA, not applicable; ORs, odds ratios; RCTs, randomised-controlled trials; SMDs, standardised mean differences.

Table S9. Summary Results from Network Meta-Analysis of Secondary Outcomes: Finding from RCTs vs. RCTs/Non-Randomised Studies (Continued)
(D) Dry Cough

Effect Estimate: ORs (95% CIs)		
**ACEIs**	2.83 (0.14 to 57.94)	17.40 (0.68 to 446.25)
2.93 (0.11 to 75.62)	**ARBs**	6.15 (0.07 to 522.45)
5.38 (0.32 to 89.75)	1.83 (0.02 to 134.76)	**Active Control**

Note: Network meta-analysis results for outcome from RCTs only (upper triangle: green) and RCTs/non-randomised studies (lower triangle: blue) For RCT only, SMDs >0 or ORs <1 indicate that the treatment specified in the row is more efficacious than that in the column. For RCTs/non-randomised studies results, SMDs >0 or ORs <1 indicate that the treatment specified in the column is more efficacious than that in the row column. Bold underlined results indicate statistical significance. To obtain SMDs for comparisons in the opposite direction, positive values should be converted into negative values, and vice versa. Similarly, reciprocals should be taken to obtain ORs for comparisons in the opposite direction.

Abbreviations: ACEIs, angiotensin-converting enzyme inhibitors; ARBs, angiotensin II receptor blockers; CIs, confidence intervals; NA, not applicable; ORs, odds ratios; RCTs, randomised-controlled trials; SMDs, standardised mean differences.

Table S9. Summary Results from Network Meta-Analysis of Secondary Outcomes: Finding from RCTs vs. RCTs/ Non-Randomised Studies (Continued)
(E) Hypotension

**Effect Estimate: ORs (95% CIs)**		
**ACEIs**	NA	NA
0.46 (0.01 to 29.13)	**MRAs**	1.25 (0.12 to 12.64)
0.58 (0.02 to 17.98)	1.25 (0.12 to 12.64)	**Active Control**

Note: Network meta-analysis results for outcome from RCTs only (upper triangle: green) and RCTs/non-randomised studies (lower triangle: blue) For RCT only, SMDs >0 or ORs <1 indicate that the treatment specified in the row is more efficacious than that in the column. For RCTs/non-randomised studies results, SMDs >0 or ORs <1 indicate that the treatment specified in the column is more efficacious than that in the row column. Bold underlined results indicate statistical significance. To obtain SMDs for comparisons in the opposite direction, positive values should be converted into negative values, and vice versa. Similarly, reciprocals should be taken to obtain ORs for comparisons in the opposite direction.

Abbreviations: ACEIs, angiotensin-converting enzyme inhibitors; ARBs, angiotensin II receptor blockers; CIs, confidence intervals; MRAs, mineralocorticoid receptor antagonists; NA, not applicable; ORs, odds ratios; RCTs, randomised-controlled trials; SMDs, standardised mean differences.

Table S9. Summary Results from Network Meta-Analysis of Secondary Outcomes: Finding from RCTs vs. RCTs/Non-Randomised Studies (Continued)
(F) Dizziness

**Effect Estimate: ORs (95% CIs)**		
**ACEIs**	NA	NA
3.11 (0.03 to 379.55)	**MRAs**	NA
6.56 (0.18 to 243.86)	2.11 (0.09 to 50.03)	**Active Control**

Note: Network meta-analysis results for outcome from RCTs only (upper triangle: green) and RCTs/non-randomised studies (lower triangle: blue) For RCT only, SMDs >0 or ORs <1 indicate that the treatment specified in the row is more efficacious than that in the column. For RCTs/non-randomised studies results, SMDs >0 or ORs <1 indicate that the treatment specified in the column is more efficacious than that in the row column. Bold underlined results indicate statistical significance. To obtain SMDs for comparisons in the opposite direction, positive values should be converted into negative values, and vice versa. Similarly, reciprocals should be taken to obtain ORs for comparisons in the opposite direction.

Abbreviations: ACEIs, angiotensin-converting enzyme inhibitors; ARBs, angiotensin II receptor blockers; CIs, confidence intervals; MRAs, mineralocorticoid receptor antagonists; NA, not applicable; ORs, odds ratios; RCTs, randomised-controlled trials; SMDs, standardised mean differences.

Table S9. Summary Results from Network Meta-Analysis of Secondary Outcomes: Finding from RCTs vs. RCTs/Non-Randomised Studies (Continued)
(G) Peritonitis

Effect Estimate: ORs (95% CIs)			
**ACEIs**	0.98 (0.35 to 2.76)	0.86 (0.28 to 2.66)	0.82 (0.36 to 1.89)
0.94 (0.34 to 2.65)	**ARBs**	0.87 (0.22 to 3.43)	0.84 (0.27 to 2.59)
0.77 (0.26 to 2.31)	0.81 (0.21 to 3.15)	**MRAs**	0.96 (0.44 to 2.06)
0.73 (0.33 to 1.61)	0.78 (0.25 2.38)	0.96 (0.44 to 2.06)	**Active Control**

Note: Network meta-analysis results for outcome from RCTs only (upper triangle: green) and RCTs/non-randomised studies (lower triangle: blue) For RCT only, SMDs >0 or ORs <1 indicate that the treatment specified in the row is more efficacious than that in the column. For RCTs/non-randomised studies results, SMDs >0 or ORs <1 indicate that the treatment specified in the column is more efficacious than that in the row column. Bold underlined results indicate statistical significance. To obtain SMDs for comparisons in the opposite direction, positive values should be converted into negative values, and vice versa. Similarly, reciprocals should be taken to obtain ORs for comparisons in the opposite direction.

Abbreviations: ACEIs, angiotensin-converting enzyme inhibitors; ARBs, angiotensin II receptor blockers; CIs, confidence intervals; MRAs, mineralocorticoid receptor antagonists; NA, not applicable; ORs, odds ratios; RCTs, randomised-controlled trials; SMDs, standardised mean differences.

Table S9. Summary Results from Network Meta-Analysis of Secondary Outcomes: Finding from RCTs vs. RCTs/Non-Randomised Studies (Continued)
(H) Hospitalisation

Effect Estimate: ORs (95% CIs)		
**ACEIs**	0.82 (0.24 to 2.81)	1.14 (0.41 to 3.17)
0.82 (0.24 to 2.81)	**ARBs**	1.39 (0.28 to 6.88)
1.14 (0.41 to 3.17)	1.39 (0.28 to 6.88)	**Active Control**

Note: Network meta-analysis results for outcome from RCTs only (upper triangle: green) and RCTs/non-randomised studies (lower triangle: blue) For RCT only, SMDs >0 or ORs <1 indicate that the treatment specified in the row is more efficacious than that in the column. For RCTs/non-randomised studies results, SMDs >0 or ORs <1 indicate that the treatment specified in the column is more efficacious than that in the row column. Bold underlined results indicate statistical significance. To obtain SMDs for comparisons in the opposite direction, positive values should be converted into negative values, and vice versa. Similarly, reciprocals should be taken to obtain ORs for comparisons in the opposite direction.

Abbreviations: ACEIs, angiotensin-converting enzyme inhibitors; ARBs, angiotensin II receptor blockers; CIs, confidence intervals; NA, not applicable; ORs, odds ratios; RCTs, randomised-controlled trials; SMDs, standardised mean differences.

Table S10. Pairwise Analysis for Secondary Outcomes: Finding from RCTs and Non-Randomised Studies

**Treatment Comparison**	**No. of Studies**	**No. of Participants**	**Effect Estimate (95% CI)**	***P* Value**	**Heterogeneity**			
					***Q* Statistic**	***P* Value**	**I^2 (95% CI)**	**τ^2**
**4-hour UF Volume by PET (mL/4 hour)**								
ACEIs vs. active control	2	52	SMD -0.36 (-1.40 to 0.68)	0.500	3.15	0.076	69% (NA)	0.389
ARBs vs. active control	1	7	SMD -0.19 (-1.24 to 0.86)	0.717	NA	NA	NA	NA
MRAs vs. active control	NA	NA	NA	NA	NA	NA	NA	NA
Mixed ACEIs/ARBs vs. active control	1	66	SMD 1.36 (0.82 to 1.90)	<0.001	NA	NA	NA	NA
ACEIs vs. ARBs	NA	NA	NA	NA	NA	NA	NA	NA
**Daily UF Volume (mL/day)**								
ACEIs vs. active control	2	89	SMD 0.74 (-0.35 to 1.83)	0.183	5.73	0.017	83% (NA)	0.513
ARBs vs. active control	1	8	SMD -0.34 (-1.33 to 0.65)	0.499	NA	NA	NA	NA
MRAs vs. active control	NA	NA	NA	NA	NA	NA	NA	NA
Mixed ACEIs/ARBs vs. active control	NA	NA	NA	NA	NA	NA	NA	NA
ACEIs vs. ARBs	NA	NA	NA	NA	NA	NA	NA	NA
**Hyperkalaemia**								
ACEIs vs. active control	3	149	OR 1.44 (0.18 to 11.69)	0.734	0.78	0.677	0% (0-90)	<0.001
ARBs vs. active control	1	7	OR 1.00 (0.02 to 58.43)	1.000	NA	NA	NA	NA
MRAs vs. active control	2	178	OR 2.07 (0.29 to 15.03)	0.472	0.00	0.995	0% (NA)	<0.001
Mixed ACEIs/ARBs vs. active control	NA	NA	NA	NA	NA	NA	NA	NA
ACEIs vs. ARBs	2	81	OR 1.21 (0.36 to 4.06)	0.758	0.01	0.921	0% (NA)	<0.001

Abbreviations: ACEIs, angiotensin-converting enzyme inhibitors; ARBs, angiotensin II receptor blockers; CIs, confidence intervals; MRAs, mineralocorticoid receptor antagonists; NA, not applicable; ORs, odds ratios; PET, peritoneal equilibration test; RCTs, randomised-controlled trials; SMDs, standardised mean differences; UF, ultrafiltration.

Table S10. Pairwise Analysis for Secondary Outcomes: Finding from RCTs and Non-Randomised Studies (Continued)

**Treatment Comparison**	**No. of Studies**	**No. of Participants**	**Effect Estimate (95% CI)**	***P* Value**	**Heterogeneity**			
					***Q* Statistic**	***P* Value**	**I^2 (95% CI)**	**τ^2**
**Dry cough**								
ACEIs vs. active control	3	176	OR 5.38 (0.34 to 85.78)	0.234	5.29	0.071	62% (0-89)	3.726
ARBs vs. active control	NA	NA	NA	NA	NA	NA	NA	NA
MRAs vs. active control	NA	NA	NA	NA	NA	NA	NA	NA
Mixed ACEIs/ARBs vs. active control	NA	NA	NA	NA	NA	NA	NA	NA
ACEIs vs. ARBs	2	81	OR 0.34 (0.01 to 9.76)	0.525	3.78	0.052	74% (44-88)	4.409
**Hypotension**								
ACEIs vs. active control	1	54	OR 0.58 (0.03 to 9.74)	0.702	NA	NA	NA	NA
ARBs vs. active control	NA	NA	NA	NA	NA	NA	NA	NA
MRAs vs. active control	1		OR 1.25 (0.37 to 4.28)	0.722	NA	NA	NA	NA
Mixed ACEIs/ARBs vs. active control	NA	NA	NA	NA	NA	NA	NA	NA
ACEIs vs. ARBs	NA	NA	NA	NA	NA	NA	NA	NA
**Dizziness**								
ACEIs vs. active control	1	60	OR 6.56 (0.31 to 136.92)	0.225	NA	NA	NA	NA
ARBs vs. active control	NA	NA	NA	NA	NA	NA	NA	NA
MRAs vs. active control	1	20	OR 2.11 (0.18 to 25.35)		NA	NA	NA	NA
Mixed ACEIs/ARBs vs. active control	NA	NA	NA	NA	NA	NA	NA	NA
ACEIs vs. ARBs	NA	NA	NA	NA	NA	NA	NA	NA

Abbreviations: ACEIs, angiotensin-converting enzyme inhibitors; ARBs, angiotensin II receptor blockers; CIs, confidence intervals; MRAs, mineralocorticoid receptor antagonists; NA, not applicable; ORs, odds ratios; RCTs, randomised-controlled trials; SMDs, standardised mean differences.

Table S10. Pairwise Analysis for Secondary Outcomes (Finding from RCTs and Non-Randomised Studies) (Continued)

**Treatment Comparison**	**No. of Studies**	**No. of Participants**	**Effect Estimate (95% CI)**	***P* Value**	**Heterogeneity**			
					***Q* Statistic**	***P* Value**	**I^2 (95% CI)**	**τ^2**
**Gynaecomastia**								
ACEIs vs. active control	NA	NA	NA	NA	NA	NA	NA	NA
ARBs vs. active control	NA	NA	NA	NA	NA	NA	NA	NA
MRAs vs. active control	1	158	OR 6.40 (1.37 to 29.92)	0.018	NA	NA	NA	NA
Mixed ACEIs/ARBs vs. active control	NA	NA	NA	NA	NA	NA	NA	NA
ACEIs vs. ARBs	NA	NA	NA	NA	NA	NA	NA	NA
**Peritonitis**								
ACEIs vs. active control	3	176	OR 0.79 (0.34 to 1.84)	0.578	1.40	0.497	0% (0-90)	<0.001
ARBs vs. active control	1	34	OR 0.60 (0.11 to 3.21)	0.551	NA	NA	NA	NA
MRAs vs. active control	1	158	OR 0.96 (0.44 to 2.06)	0.909	NA	NA	NA	NA
Mixed ACEIs/ARBs vs. active control	NA	NA	NA	NA	NA	NA	NA	NA
ACEIs vs. ARBs	1	60	OR 1.22 (0.36 to 4.17)	0.754	NA	NA	NA	NA
**Hospitalisation**								
ACEIs vs. active control	1	60	OR 1.14 (0.41 to 3.17)	0.795	NA	NA	NA	NA
ARBs vs. active control	NA	NA	NA	NA	NA	NA	NA	NA
MRAs vs. active control	NA	NA	NA	NA	NA	NA	NA	NA
Mixed ACEIs/ARBs vs. active control	NA	NA	NA	NA	NA	NA	NA	NA
ACEIs vs. ARBs	1	60	OR 1.17 (0.54 to 2.57)	0.754	NA	NA	NA	NA

Abbreviations: ACEIs, angiotensin-converting enzyme inhibitors; ARBs, angiotensin II receptor blockers; CIs, confidence intervals; MRAs, mineralocorticoid receptor antagonists; NA, not applicable; ORs, odds ratios; RCTs, randomised-controlled trials; SMDs, standardised mean differences.

Table S11. Sensitivity Analysis: Assuming the Correlation Coefficient of 0.56 for Estimating the SD_{Change} for Continuous Outcomes (Finding from RCTs and Non-Randomised Studies)

(A) Residual Glomerular Filtration Rate (mL/min/1.73 m^2)

**Effect Estimate: SMDs (95% CIs)**			
**ACEIs**	-0.10 (-0.69 to 0.49)	NA	0.52 (-0.07 to 1.11)
-0.07 (-0.56 to 0.42)	**ARBs**	NA	**0.62 (0.10 to 1.15)**
0.10 (-0.56 to 0.76)	0.17 (-0.44 to 0.78)	**Mixed ACEIs/ARBs**	NA
**0.55 (0.06 to 1.04)**	**0.62 (0.19 to 1.05)**	**0.45 (0.02 to 0.88)**	**Active Control**

Note: Network meta-analysis results for outcome from RCTs only (upper triangle: green) and RCTs/non-randomised studies (lower triangle: blue) For RCT only, SMDs >0 indicate that the treatment specified in the row is more efficacious than that in the column. For RCTs/non-randomised studies results, SMDs >0 indicate that the treatment specified in the column is more efficacious than that in the row column. Bold underlined results indicate statistical significance. To obtain SMDs for comparisons in the opposite direction, positive values should be converted into negative values, and vice versa.

Abbreviations: ACEIs, angiotensin-converting enzyme inhibitors; ARBs, angiotensin II receptor blockers; CIs, confidence intervals; NA, not applicable; RCTs, randomised-controlled trials; SMDs, standardised mean differences.

Table S11. Sensitivity Analysis: Assuming the Correlation Coefficient of 0.56 for Estimating the SD_{Change} for Continuous Outcomes (Finding from RCTs and Non-Randomised Studies) (Continued)

(B) Urine Volume (mL/day)

Effect Estimate: SMDs (95% CIs)			
**ACEIs**	NA	NA	NA
-0.89 (-3.87 to 2.08)	**ARBs**	1.64 (-1.72 to 5.01)	1.41 (-0.29 to 3.10)
0.44 (-3.30 to 4.19)	1.33 (-1.63 to 4.30)	**MRAs**	-0.24 (-3.14 to 2.67)
0.20 (-2.45 to 2.86)	1.10 (-0.25 to 2.44)	-0.24 (-2.88 to 2.41)	**Active Control**

Note: Network meta-analysis results for outcome from RCTs only (upper triangle: green) and RCTs/non-randomised studies (lower triangle: blue) For RCT only, SMDs >0 indicate that the treatment specified in the row is more efficacious than that in the column. For RCTs/non-randomised studies results, SMDs >0 indicate that the treatment specified in the column is more efficacious than that in the row column. Bold underlined results indicate statistical significance. To obtain SMDs for comparisons in the opposite direction, positive values should be converted into negative values, and vice versa.

Abbreviations: ACEIs, angiotensin-converting enzyme inhibitors; ARBs, angiotensin II receptor blockers; CIs, confidence intervals; MRAs, mineralocorticoid receptor antagonists; NA, not applicable; RCTs, randomised-controlled trials; SMDs, standardised mean differences.

Table S11. Sensitivity Analysis: Assuming the Correlation Coefficient of 0.56 for Estimating the SD_{Change} for Continuous Outcomes (Finding from RCTs and Non-Randomised Studies) (Continued)

(C) Dialysate-to-Plasma Creatinine Ratio

**Effect Estimate: ORs (95% CIs)**		
**ARBs**	NA	NA
**1.64 (0.87 to 2.41)**	**Mixed ACEIs/ARBs**	NA
0.04 (-0.48 to 0.57)	**-1.60 (-2.16 to -1.04)**	**Active Control**

Note: Network meta-analysis results for outcome from RCTs only (upper triangle: green) and RCTs/non-randomised studies (lower triangle: blue) For RCT only, SMDs >0 indicate that the treatment specified in the row is more efficacious than that in the column. For RCTs/non-randomised studies results, SMDs >0 indicate that the treatment specified in the column is more efficacious than that in the row column. Bold underlined results indicate statistical significance. To obtain SMDs for comparisons in the opposite direction, positive values should be converted into negative values, and vice versa.

Abbreviations: ACEIs, angiotensin-converting enzyme inhibitors; ARBs, angiotensin II receptor blockers; CIs, confidence intervals; NA, not applicable; RCTs, randomised-controlled trials; SMDs, standardised mean differences.

Table S11. Sensitivity Analysis: Assuming the Correlation Coefficient of 0.56 for Estimating the SD_{Change} for Continuous Outcomes (Finding from RCTs and Non-Randomised Studies) (Continued)

(D) 4-Hour Ultrafiltration Volume by Peritoneal Equilibration Test (mL/4 hour)

Effect Estimate: SMDs (95% CIs)			
**ACEIs**	NA	NA	NA
-0.17 (-2.10 to 1.76)	**ARBs**	NA	NA
**-1.72 (-3.43 to -0.02)**	-1.56 (-3.66 to 0.55)	**MRAs**	NA
-0.36 (-1.42 to 0.69)	-0.19 (-1.81 to 1.42)	**1.36 (0.02 to 2.71)**	**Active Control**

Note: Network meta-analysis results for outcome from RCTs only (upper triangle: green) and RCTs/non-randomised studies (lower triangle: blue) For RCT only, SMDs >0 indicate that the treatment specified in the row is more efficacious than that in the column. For RCTs/non-randomised studies results, SMDs >0 indicate that the treatment specified in the column is more efficacious than that in the row column. Bold underlined results indicate statistical significance. To obtain SMDs for comparisons in the opposite direction, positive values should be converted into negative values, and vice versa.

Abbreviations: ACEIs, angiotensin-converting enzyme inhibitors; ARBs, angiotensin II receptor blockers; CIs, confidence intervals; MRAs, mineralocorticoid receptor antagonists; NA, not applicable; RCTs, randomised-controlled trials; SMDs, standardised mean differences.

Table S11. Sensitivity Analysis: Assuming the Correlation Coefficient of 0.56 for Estimating the SD_{Change} for Continuous Outcomes (Finding from RCTs and Non-Randomised Studies) (Continued)

(E) Daily Ultrafiltration Volume (mL/day)

Effect Estimate: ORs (95% CIs)		
**ARBs**	NA	NA
1.09 (-0.95 to 3.12)	**Mixed ACEIs/ARBs**	NA
0.75 (-0.35 to 1.84)	-0.34 (-2.06 to 1.38)	**Active Control**

Note: Network meta-analysis results for outcome from RCTs only (upper triangle: green) and RCTs/non-randomised studies (lower triangle: blue) For RCT only, SMDs >0 indicate that the treatment specified in the row is more efficacious than that in the column. For RCTs/non-randomised studies results, SMDs >0 indicate that the treatment specified in the column is more efficacious than that in the row column. Bold underlined results indicate statistical significance. To obtain SMDs for comparisons in the opposite direction, positive values should be converted into negative values, and vice versa.

Abbreviations: ACEIs, angiotensin-converting enzyme inhibitors; ARBs, angiotensin II receptor blockers; CIs, confidence intervals; NA, not applicable; RCTs, randomised-controlled trials; SMDs, standardised mean differences.

Table S12. Sensitivity Analysis: Excluding Studies from Mainland China (Finding from RCTs and Non-Randomised Studies)

(A) Residual Glomerular Filtration Rate (mL/min/1.73 m^2)

**Effect Estimate: SMDs (95% CIs)**			
**ACEIs**	-0.14 (-0.92 to 0.64)	NA	0.52 (-0.27 to 1.32)
-0.14 (-0.92 to 0.64)	**ARBs**	NA	0.66 (-0.25 to 1.58)
0.12 (-1.22 to 1.46)	0.27 (-1.14 to 1.68)	**Mixed ACEIs/ARBs**	NA
0.52 (-0.27 to 1.32)	0.66 (-0.25 to 1.58)	0.40 (-0.68 to 1.47)	**Active Control**

Note: Network meta-analysis results for outcome from RCTs only (upper triangle: green) and RCTs/non-randomised studies (lower triangle: blue) For RCT only, SMDs >0 or ORs <1 indicate that the treatment specified in the row is more efficacious than that in the column. For RCTs/non-randomised studies results, SMDs >0 or ORs <1 indicate that the treatment specified in the column is more efficacious than that in the row column. Bold underlined results indicate statistical significance. To obtain SMDs for comparisons in the opposite direction, positive values should be converted into negative values, and vice versa. Similarly, reciprocals should be taken to obtain ORs for comparisons in the opposite direction.

Abbreviations: ACEIs, angiotensin-converting enzyme inhibitors; ARBs, angiotensin II receptor blockers; CIs, confidence intervals; NA, not applicable; ORs, odds ratios; RCTs, randomised-controlled trials; SMDs, standardised mean differences.

Table S12. Sensitivity Analysis: Excluding Studies from Mainland China (Finding from RCTs and Non-Randomised Studies) (Continued)
(B) Urine Volume (mL/day)

**Effect Estimate: SMDs (95% CIs)**			
**ACEIs**	NA	NA	NA
-1.45 (-6.58 to 3.68)	**ARBs**	**3.42 (2.30 to 4.54)**	**3.19 (2.51 to 3.87)**
0.44 (-5.45 to 6.33)	1.89 (-3.23 to 7.02)	**MRAs**	-0.24 (-1.81 to 1.34)
0.20 (-3.96 to 4.37)	1.65 (-1.34 to 4.65)	-0.24 (-4.40 to 3.92)	**Active Control**

Note: Network meta-analysis results for outcome from RCTs only (upper triangle: green) and RCTs/non-randomised studies (lower triangle: blue) For RCT only, SMDs >0 or ORs <1 indicate that the treatment specified in the row is more efficacious than that in the column. For RCTs/non-randomised studies results, SMDs >0 or ORs <1 indicate that the treatment specified in the column is more efficacious than that in the row column. Bold underlined results indicate statistical significance. To obtain SMDs for comparisons in the opposite direction, positive values should be converted into negative values, and vice versa. Similarly, reciprocals should be taken to obtain ORs for comparisons in the opposite direction.

Abbreviations: ACEIs, angiotensin-converting enzyme inhibitors; ARBs, angiotensin II receptor blockers; CIs, confidence intervals; MRAs, mineralocorticoid receptor antagonists; NA, not applicable; ORs, odds ratios; RCTs, randomised-controlled trials; SMDs, standardised mean differences.

Table S12. Sensitivity Analysis: Excluding Studies from Mainland China (Finding from RCTs and Non-Randomised Studies) (Continued)
(C) Incidence of Anuria

Effect Estimate: ORs (95% CIs)			
**ACEIs**	0.92 (0.48 to 1.75)	NA	**0.58 (0.36 to 0.94)**
0.92 (0.48 to 1.75)	**ARBs**	NA	0.63 (0.28 to 1.42)
**0.78 (0.61 to 0.99)**	0.85 (0.42 to 1.69)	**Mixed ACEIs/ARBs**	NA
**0.69 (0.57 to 0.83)**	0.75 (0.38 to 1.47)	0.88 (0.75 to 1.03)	**Active Control**

Note: Network meta-analysis results for outcome from RCTs only (upper triangle: green) and RCTs/non-randomised studies (lower triangle: blue) For RCT only, SMDs >0 or ORs <1 indicate that the treatment specified in the row is more efficacious than that in the column. For RCTs/non-randomised studies results, SMDs >0 or ORs <1 indicate that the treatment specified in the column is more efficacious than that in the row column. Bold underlined results indicate statistical significance. To obtain SMDs for comparisons in the opposite direction, positive values should be converted into negative values, and vice versa. Similarly, reciprocals should be taken to obtain ORs for comparisons in the opposite direction.

Abbreviations: ACEIs, angiotensin-converting enzyme inhibitors; ARBs, angiotensin II receptor blockers; CIs, confidence intervals; NA, not applicable; ORs, odds ratios; RCTs, randomised-controlled trials; SMDs, standardised mean differences.

Table S12. Sensitivity Analysis: Excluding Studies from Mainland China (Finding from RCTs and Non-Randomised Studies) (Continued)
(D) Dialysate-to-Plasma Creatinine Ratio

Effect Estimate: SMDs (95% CIs)		
**ARBs**	NA	NA
NA	**Mixed ACEIs/ARBs**	NA
0.04 (-0.48 to 0.57)	NA	**Active Control**

Note: Network meta-analysis results for outcome from RCTs only (upper triangle: green) and RCTs/non-randomised studies (lower triangle: blue) For RCT only, SMDs >0 or ORs <1 indicate that the treatment specified in the row is more efficacious than that in the column. For RCTs/non-randomised studies results, SMDs >0 or ORs <1 indicate that the treatment specified in the column is more efficacious than that in the row column. Bold underlined results indicate statistical significance. To obtain SMDs for comparisons in the opposite direction, positive values should be converted into negative values, and vice versa. Similarly, reciprocals should be taken to obtain ORs for comparisons in the opposite direction.

Abbreviations: ACEIs, angiotensin-converting enzyme inhibitors; ARBs, angiotensin II receptor blockers; CIs, confidence intervals; NA, not applicable; ORs, odds ratios; RCTs, randomised-controlled trials; SMDs, standardised mean differences.

Table S12. Sensitivity Analysis: Excluding Studies from Mainland China (Finding from RCTs and Non-Randomised Studies) (Continued)
(E) Acceptability of Treatment

Effect Estimate: ORs (95% CIs)			
**ACEIs**	1.20 (0.11 to 12.80)	1.06 (0.24 to 4.72)	1.52 (0.54 to 4.27)
0.97 (0.15 to 6.11)	**ARBs**	0.88 (0.06 to 12.81)	1.27 (0.11 to 14.65)
0.65 (0.12 to 3.66)	0.67 (0.07 to 6.36)	**MRAs**	1.44 (0.49 to 4.21)
0.92 (0.34 to 2.51)	0.95 (0.17 to 5.50)	1.41 (0.35 to 5.74)	**Active Control**

Note: Network meta-analysis results for outcome from RCTs only (upper triangle: green) and RCTs/non-randomised studies (lower triangle: blue) For RCT only, SMDs >0 or ORs <1 indicate that the treatment specified in the row is more efficacious than that in the column. For RCTs/non-randomised studies results, SMDs >0 or ORs <1 indicate that the treatment specified in the column is more efficacious than that in the row column. Bold underlined results indicate statistical significance. To obtain SMDs for comparisons in the opposite direction, positive values should be converted into negative values, and vice versa. Similarly, reciprocals should be taken to obtain ORs for comparisons in the opposite direction.

Abbreviations: ACEIs, angiotensin-converting enzyme inhibitors; ARBs, angiotensin II receptor blockers; CIs, confidence intervals; MRAs, mineralocorticoid receptor antagonists; NA, not applicable; ORs, odds ratios; RCTs, randomised-controlled trials; SMDs, standardised mean differences.

Table S12. Sensitivity Analysis: Excluding Studies from Mainland China (Finding from RCTs and Non-Randomised Studies) (Continued)

(F) 4-Hour Ultrafiltration Volume by Peritoneal Equilibration Test (mL/4 hour)

**Effect Estimate: SMDs (95% CIs)**			
**ACEIs**	NA	NA	0.96 (-1.24 to 3.15)
-0.16 (-2.09 to 1.76)	**ARBs**	NA	NA
NA	NA	**Mixed ACEIs/ARBs**	NA
-0.36 (-1.41 to 0.70)	-0.16 (-1.81 to 1.42)	NA	**Active Control**

Note: Network meta-analysis results for outcome from RCTs only (upper triangle: green) and RCTs/non-randomised studies (lower triangle: blue) For RCT only, SMDs >0 or ORs <1 indicate that the treatment specified in the row is more efficacious than that in the column. For RCTs/non-randomised studies results, SMDs >0 or ORs <1 indicate that the treatment specified in the column is more efficacious than that in the row column. Bold underlined results indicate statistical significance. To obtain SMDs for comparisons in the opposite direction, positive values should be converted into negative values, and vice versa. Similarly, reciprocals should be taken to obtain ORs for comparisons in the opposite direction.

Abbreviations: ACEIs, angiotensin-converting enzyme inhibitors; ARBs, angiotensin II receptor blockers; CIs, confidence intervals; NA, not applicable; ORs, odds ratios; RCTs, randomised-controlled trials; SMDs, standardised mean differences.

Table S12. Sensitivity Analysis: Excluding Studies from Mainland China (Finding from RCTs and Non-Randomised Studies) (Continued)
(G) Daily Ultrafiltration Volume (mL/day)

**Effect Estimate: SMDs (95% CIs)**		
**ACEIs**	NA	-1.30 (-3.97 to 1.38)
1.08 (-0.95 to 3.12)	**ARBs**	NA
0.74 (-0.35 to 1.83)	-0.34 (-2.06 to 1.38)	**Active Control**

Note: Network meta-analysis results for outcome from RCTs only (upper triangle: green) and RCTs/non-randomised studies (lower triangle: blue) For RCT only, SMDs >0 or ORs <1 indicate that the treatment specified in the row is more efficacious than that in the column. For RCTs/non-randomised studies results, SMDs >0 or ORs <1 indicate that the treatment specified in the column is more efficacious than that in the row column. Bold underlined results indicate statistical significance. To obtain SMDs for comparisons in the opposite direction, positive values should be converted into negative values, and vice versa. Similarly, reciprocals should be taken to obtain ORs for comparisons in the opposite direction.

Abbreviations: ACEIs, angiotensin-converting enzyme inhibitors; ARBs, angiotensin II receptor blockers; CIs, confidence intervals; NA, not applicable; ORs, odds ratios; RCTs, randomised-controlled trials; SMDs, standardised mean differences.

Table S12. Sensitivity Analysis: Excluding Studies from Mainland China (Finding from RCTs and Non-Randomised Studies) (Continued)
(H) Hyperkalaemia

Effect Estimate: ORs (95% CIs)			
**ACEIs**	0.83 (0.25 to 2.78)	1.11 (0.05 to 26.27)	2.30 (0.20 to 27.06)
0.86 (0.27 to 2.77)	**ARBs**	1.34 (0.05 to 39.75)	2.78 (0.18 to 43.36)
0.62 (0.04 to 9.56)	0.73 (0.04 to 12.86)	**MRAs**	2.07 (0.29 to 15.03)
1.29 (0.20 to 8.44)	1.50 (0.19 to 12.04)	2.07 (0.29 to 15.03)	**Active Control**

Note: Network meta-analysis results for outcome from RCTs only (upper triangle: green) and RCTs/non-randomised studies (lower triangle: blue) For RCT only, SMDs >0 or ORs <1 indicate that the treatment specified in the row is more efficacious than that in the column. For RCTs/non-randomised studies results, SMDs >0 or ORs <1 indicate that the treatment specified in the column is more efficacious than that in the row column. Bold underlined results indicate statistical significance. To obtain SMDs for comparisons in the opposite direction, positive values should be converted into negative values, and vice versa. Similarly, reciprocals should be taken to obtain ORs for comparisons in the opposite direction.

Abbreviations: ACEIs, angiotensin-converting enzyme inhibitors; ARBs, angiotensin II receptor blockers; CIs, confidence intervals; MRAs, mineralocorticoid receptor antagonists; NA, not applicable; ORs, odds ratios; RCTs, randomised-controlled trials; SMDs, standardised mean differences.

Table S12. Sensitivity Analysis: Excluding Studies from Mainland China (Finding from RCTs and Non-Randomised Studies) (Continued)
(I) Dry Cough

Effect Estimate: ORs (95% CIs)		
**ACEIs**	2.83 (0.14 to 57.94)	17.40 (0.68 to 446.25)
2.93 (0.11 to 75.62)	**ARBs**	6.15 (0.07 to 522.45)
5.38 (0.32 to 89.75)	1.83 (0.02 to 134.76)	**Active Control**

Note: Network meta-analysis results for outcome from RCTs only (upper triangle: green) and RCTs/non-randomised studies (lower triangle: blue) For RCT only, SMDs >0 or ORs <1 indicate that the treatment specified in the row is more efficacious than that in the column. For RCTs/non-randomised studies results, SMDs >0 or ORs <1 indicate that the treatment specified in the column is more efficacious than that in the row column. Bold underlined results indicate statistical significance. To obtain SMDs for comparisons in the opposite direction, positive values should be converted into negative values, and vice versa. Similarly, reciprocals should be taken to obtain ORs for comparisons in the opposite direction.

Abbreviations: ACEIs, angiotensin-converting enzyme inhibitors; ARBs, angiotensin II receptor blockers; CIs, confidence intervals; NA, not applicable; ORs, odds ratios; RCTs, randomised-controlled trials; SMDs, standardised mean differences.

Table S12. Sensitivity Analysis: Excluding Studies from Mainland China (Finding from RCTs and Non-Randomised Studies) (Continued)

(J) Hypotension

Effect Estimate: ORs (95% CIs)		
**ACEIs**	NA	NA
0.46 (0.01 to 29.13)	**MRAs**	0.80 (0.00 to 1155.18)
0.58 (0.02 to 17.98)	1.25 (0.12 to 12.64)	**Active Control**

Note: Network meta-analysis results for outcome from RCTs only (upper triangle: green) and RCTs/non-randomised studies (lower triangle: blue) For RCT only, SMDs >0 or ORs <1 indicate that the treatment specified in the row is more efficacious than that in the column. For RCTs/non-randomised studies results, SMDs >0 or ORs <1 indicate that the treatment specified in the column is more efficacious than that in the row column. Bold underlined results indicate statistical significance. To obtain SMDs for comparisons in the opposite direction, positive values should be converted into negative values, and vice versa. Similarly, reciprocals should be taken to obtain ORs for comparisons in the opposite direction.

Abbreviations: ACEIs, angiotensin-converting enzyme inhibitors; ARBs, angiotensin II receptor blockers; CIs, confidence intervals; MRAs, mineralocorticoid receptor antagonists; NA, not applicable; ORs, odds ratios; RCTs, randomised-controlled trials; SMDs, standardised mean differences.

Table S12. Sensitivity Analysis: Excluding Studies from Mainland China (Finding from RCTs and Non-Randomised Studies) (Continued)
(K) Dizziness

**Effect Estimate: ORs (95% CIs)**		
**ACEIs**	3.11 (0.03 to 379.55)	6.56 (0.18 to 243.86)
3.11 (0.03 to 379.55)	**MRAs**	2.11 (0.09 to 50.03)
6.56 (0.18 to 243.86)	2.11 (0.09 to 50.03)	**Active Control**

Note: Network meta-analysis results for outcome from RCTs only (upper triangle: green) and RCTs/non-randomised studies (lower triangle: blue) For RCT only, SMDs >0 or ORs <1 indicate that the treatment specified in the row is more efficacious than that in the column. For RCTs/non-randomised studies results, SMDs >0 or ORs <1 indicate that the treatment specified in the column is more efficacious than that in the row column. Bold underlined results indicate statistical significance. To obtain SMDs for comparisons in the opposite direction, positive values should be converted into negative values, and vice versa. Similarly, reciprocals should be taken to obtain ORs for comparisons in the opposite direction.

Abbreviations: ACEIs, angiotensin-converting enzyme inhibitors; ARBs, angiotensin II receptor blockers; CIs, confidence intervals; MRAs, mineralocorticoid receptor antagonists; NA, not applicable; ORs, odds ratios; RCTs, randomised-controlled trials; SMDs, standardised mean differences.

Table S12. Sensitivity Analysis: Excluding Studies from Mainland China (Finding from RCTs and Non-Randomised Studies) (Continued)
(L) Peritonitis

Effect Estimate: ORs (95% CIs)			
**ACEIs**	0.98 (0.35 to 2.76)	0.86 (0.28 to 2.66)	0.82 (0.36 to 1.89)
0.94 (0.34 to 2.65)	**ARBs**	0.87 (0.22 to 3.43)	0.84 (0.27 to 2.59)
0.77 (0.26 to 2.31)	0.81 (0.21 to 3.15)	**MRAs**	0.96 (0.44 to 2.06)
0.73 (0.33 to 1.61)	0.78 (0.25 to 2.38)	0.96 (0.44 to 2.06)	**Active Control**

Note: Network meta-analysis results for outcome from RCTs only (upper triangle: green) and RCTs/non-randomised studies (lower triangle: blue) For RCT only, SMDs >0 or ORs <1 indicate that the treatment specified in the row is more efficacious than that in the column. For RCTs/non-randomised studies results, SMDs >0 or ORs <1 indicate that the treatment specified in the column is more efficacious than that in the row column. Bold underlined results indicate statistical significance. To obtain SMDs for comparisons in the opposite direction, positive values should be converted into negative values, and vice versa. Similarly, reciprocals should be taken to obtain ORs for comparisons in the opposite direction.

Abbreviations: ACEIs, angiotensin-converting enzyme inhibitors; ARBs, angiotensin II receptor blockers; CIs, confidence intervals; MRAs, mineralocorticoid receptor antagonists; NA, not applicable; ORs, odds ratios; RCTs, randomised-controlled trials; SMDs, standardised mean differences.

Table S12. Sensitivity Analysis: Excluding Studies from Mainland China (Finding from RCTs and Non-Randomised Studies) (Continued)

(M) Hospitalisation

**Effect Estimate: ORs (95% CIs)**		
**ACEIs**	0.82 (0.24 to 2.81)	1.14 (0.41 to 3.17)
0.82 (0.24 to 2.81)	**ARBs**	1.39 (0.28 to 6.88)
1.14 (0.41 to 3.17)	1.39 (0.28 to 6.88)	**Active Control**

Note: Network meta-analysis results for outcome from RCTs only (upper triangle: green) and RCTs/non-randomised studies (lower triangle: blue) For RCT only, SMDs >0 or ORs <1 indicate that the treatment specified in the row is more efficacious than that in the column. For RCTs/non-randomised studies results, SMDs >0 or ORs <1 indicate that the treatment specified in the column is more efficacious than that in the row column. Bold underlined results indicate statistical significance. To obtain SMDs for comparisons in the opposite direction, positive values should be converted into negative values, and vice versa. Similarly, reciprocals should be taken to obtain ORs for comparisons in the opposite direction.

Abbreviations: ACEIs, angiotensin-converting enzyme inhibitors; ARBs, angiotensin II receptor blockers; CIs, confidence intervals; NA, not applicable; ORs, odds ratios; RCTs, randomised-controlled trials; SMDs, standardised mean differences.

Table S13. Sensitivity Analysis: Excluding Crossover Studies (Finding from RCTs and Non-Randomised Studies)
(A) Residual Glomerular Filtration Rate (mL/min/1.73 m^2)

**Effect Estimate: SMDs (95% CIs)**			
**ACEIs**	0.04 (-0.59 to 0.66)	NA	**0.77 (0.15 to 1.40)**
0.06 (-0.35 to 0.47)	**ARBs**	NA	**0.74 (0.24 to 1.23)**
0.35 (-0.10 to 0.80)	0.29 (-0.11 to 0.68)	**Mixed ACEIs/ARBs**	NA
**0.76 (0.34 to 1.18)**	**0.70 (0.34 to 1.06)**	**0.41 (0.25 to 0.57)**	**Active Control**

Note: Network meta-analysis results for outcome from RCTs only (upper triangle: green) and RCTs/non-randomised studies (lower triangle: blue) For RCT only, SMDs >0 or ORs <1 indicate that the treatment specified in the row is more efficacious than that in the column. For RCTs/non-randomised studies results, SMDs >0 or ORs <1 indicate that the treatment specified in the column is more efficacious than that in the row column. Bold underlined results indicate statistical significance. To obtain SMDs for comparisons in the opposite direction, positive values should be converted into negative values, and vice versa. Similarly, reciprocals should be taken to obtain ORs for comparisons in the opposite direction.

Abbreviations: ACEIs, angiotensin-converting enzyme inhibitors; ARBs, angiotensin II receptor blockers; CIs, confidence intervals; NA, not applicable; ORs, odds ratios; RCTs, randomised-controlled trials; SMDs, standardised mean differences.

Table S13. Sensitivity Analysis: Excluding Crossover Studies (Finding from RCTs and Non-Randomised Studies) (Continued)
(B) Urine Volume (mL/day)

Effect Estimate: SMDs (95% CIs)			
**ACEIs**	NA	NA	NA
-1.19 (-4.52 to 2.15)	**ARBs**	NA	1.39 (-0.29 to 3.08)
NA	NA	**MRAs**	NA
0.20 (-2.67 to 3.08)	1.39 (-0.29 to 3.08)	NA	**Active Control**

Note: Network meta-analysis results for outcome from RCTs only (upper triangle: green) and RCTs/non-randomised studies (lower triangle: blue) For RCT only, SMDs >0 or ORs <1 indicate that the treatment specified in the row is more efficacious than that in the column. For RCTs/non-randomised studies results, SMDs >0 or ORs <1 indicate that the treatment specified in the column is more efficacious than that in the row column. Bold underlined results indicate statistical significance. To obtain SMDs for comparisons in the opposite direction, positive values should be converted into negative values, and vice versa. Similarly, reciprocals should be taken to obtain ORs for comparisons in the opposite direction.

Abbreviations: ACEIs, angiotensin-converting enzyme inhibitors; ARBs, angiotensin II receptor blockers; CIs, confidence intervals; MRAs, mineralocorticoid receptor antagonists; NA, not applicable; ORs, odds ratios; RCTs, randomised-controlled trials; SMDs, standardised mean differences.

Table S13. Sensitivity Analysis: Excluding Crossover Studies (Finding from RCTs and Non-Randomised Studies) (Continued)
(C) Incidence of Anuria

Effect Estimate: ORs (95% CIs)			
**ACEIs**	0.81 (0.48 to 1.34)	NA	**0.62 (0.41 to 0.95)**
0.85 (0.53 to 1.36)	**ARBs**	NA	0.77 (0.46 to 1.29)
0.78 (0.61 to 1.00)	0.92 (0.56 to 1.52)	**Mixed ACEIs/ARBs**	NA
**0.69 (0.57 to 0.83)**	0.81 (0.51 to 1.31)	0.88 (0.75 to 1.03)	**Active Control**

Note: Network meta-analysis results for outcome from RCTs only (upper triangle: green) and RCTs/non-randomised studies (lower triangle: blue) For RCT only, SMDs >0 or ORs <1 indicate that the treatment specified in the row is more efficacious than that in the column. For RCTs/non-randomised studies results, SMDs >0 or ORs <1 indicate that the treatment specified in the column is more efficacious than that in the row column. Bold underlined results indicate statistical significance. To obtain SMDs for comparisons in the opposite direction, positive values should be converted into negative values, and vice versa. Similarly, reciprocals should be taken to obtain ORs for comparisons in the opposite direction.

Abbreviations: ACEIs, angiotensin-converting enzyme inhibitors; ARBs, angiotensin II receptor blockers; CIs, confidence intervals; NA, not applicable; ORs, odds ratios; RCTs, randomised-controlled trials; SMDs, standardised mean differences.

Table S13. Sensitivity Analysis: Excluding Crossover Studies (Finding from RCTs and Non-Randomised Studies) (Continued)
(D) Dialysate-to-Plasma Creatinine Ratio

**Effect Estimate: SMDs (95% CIs)**		
**ARBs**	NA	NA
NA	**Mixed ACEIs/ARBs**	-1.60 (-3.64 to 0.44)
NA	-1.60 (-3.64 to 0.44)	**Active Control**

Note: Network meta-analysis results for outcome from RCTs only (upper triangle: green) and RCTs/non-randomised studies (lower triangle: blue) For RCT only, SMDs >0 or ORs <1 indicate that the treatment specified in the row is more efficacious than that in the column. For RCTs/non-randomised studies results, SMDs >0 or ORs <1 indicate that the treatment specified in the column is more efficacious than that in the row column. Bold underlined results indicate statistical significance. To obtain SMDs for comparisons in the opposite direction, positive values should be converted into negative values, and vice versa. Similarly, reciprocals should be taken to obtain ORs for comparisons in the opposite direction.

Abbreviations: ACEIs, angiotensin-converting enzyme inhibitors; ARBs, angiotensin II receptor blockers; CIs, confidence intervals; NA, not applicable; ORs, odds ratios; RCTs, randomised-controlled trials; SMDs, standardised mean differences.

Table S13. Sensitivity Analysis: Excluding Crossover Studies (Finding from RCTs and Non-Randomised Studies) (Continued)

(E) Acceptability of Treatment

Effect Estimate: ORs (95% CIs)			
**ACEIs**	1.32 (0.21 to 8.26)	1.07 (0.21 to 5.49)	1.58 (0.52 to 4.80)
0.83 (0.11 to 6.07)	**ARBs**	0.81 (0.11 to 5.93)	1.20 (0.24 to 5.86)
0.63 (0.08 to 5.25)	0.76 (0.06 to 9.10)	**MRAs**	1.48 (0.45 to 4.90)
0.93 (0.29 to 3.00)	1.12 (0.20 to 6.43)	1.48 (0.25 to 8.62)	**Active Control**

Note: Network meta-analysis results for outcome from RCTs only (upper triangle: green) and RCTs/non-randomised studies (lower triangle: blue) For RCT only, SMDs >0 or ORs <1 indicate that the treatment specified in the row is more efficacious than that in the column. For RCTs/non-randomised studies results, SMDs >0 or ORs <1 indicate that the treatment specified in the column is more efficacious than that in the row column. Bold underlined results indicate statistical significance. To obtain SMDs for comparisons in the opposite direction, positive values should be converted into negative values, and vice versa. Similarly, reciprocals should be taken to obtain ORs for comparisons in the opposite direction.

Abbreviations: ACEIs, angiotensin-converting enzyme inhibitors; ARBs, angiotensin II receptor blockers; CIs, confidence intervals; MRAs, mineralocorticoid receptor antagonists; NA, not applicable; ORs, odds ratios; RCTs, randomised-controlled trials; SMDs, standardised mean differences.

Table S13. Sensitivity Analysis: Excluding Crossover Studies (Finding from RCTs and Non-Randomised Studies) (Continued)
(F) 4-Hour Ultrafiltration Volume by Peritoneal Equilibration Test (mL/4 hour)

Effect Estimate: ORs (95% CIs)			
**ACEIs**	NA	NA	NA
NA	**ARBs**	NA	NA
-1.25 (-4.15 to 1.65)	NA	**Mixed ACEIs/ARBs**	NA
0.11 (-1.95 to 2.18)	NA	1.36 (-0.67 to 3.39)	**Active Control**

Note: Network meta-analysis results for outcome from RCTs only (upper triangle: green) and RCTs/non-randomised studies (lower triangle: blue) For RCT only, SMDs >0 or ORs <1 indicate that the treatment specified in the row is more efficacious than that in the column. For RCTs/non-randomised studies results, SMDs >0 or ORs <1 indicate that the treatment specified in the column is more efficacious than that in the row column. Bold underlined results indicate statistical significance. To obtain SMDs for comparisons in the opposite direction, positive values should be converted into negative values, and vice versa. Similarly, reciprocals should be taken to obtain ORs for comparisons in the opposite direction.

Abbreviations: ACEIs, angiotensin-converting enzyme inhibitors; ARBs, angiotensin II receptor blockers; CIs, confidence intervals; NA, not applicable; ORs, odds ratios; RCTs, randomised-controlled trials; SMDs, standardised mean differences.

Table S13. Sensitivity Analysis: Excluding Crossover Studies (Finding from RCTs and Non-Randomised Studies) (Continued)

(G) Daily Ultrafiltration Volume (mL/day)

**Effect Estimate: SMDs (95% CIs)**		
**ACEIs**	NA	-1.30 (-3.97 to 1.38)
NA	**ARBs**	NA
0.74 (-0.35 to 1.83)	NA	**Active Control**

Note: Network meta-analysis results for outcome from RCTs only (upper triangle: green) and RCTs/non-randomised studies (lower triangle: blue) For RCT only, SMDs >0 or ORs <1 indicate that the treatment specified in the row is more efficacious than that in the column. For RCTs/non-randomised studies results, SMDs >0 or ORs <1 indicate that the treatment specified in the column is more efficacious than that in the row column. Bold underlined results indicate statistical significance. To obtain SMDs for comparisons in the opposite direction, positive values should be converted into negative values, and vice versa. Similarly, reciprocals should be taken to obtain ORs for comparisons in the opposite direction.

Abbreviations: ACEIs, angiotensin-converting enzyme inhibitors; ARBs, angiotensin II receptor blockers; CIs, confidence intervals; NA, not applicable; ORs, odds ratios; RCTs, randomised-controlled trials; SMDs, standardised mean differences.

Table S13. Sensitivity Analysis: Excluding Crossover Studies (Finding from RCTs and Non-Randomised Studies) (Continued)
(H) Hyperkalaemia

**Effect Estimate: ORs (95% CIs)**			
**ACEIs**	1.00 (0.02 to 52.09)	1.11 (0.03 to 35.04)	2.30 (0.20 to 27.07)
1.00 (0.02 to 52.09)	**ARBs**	1.11 (0.01 to 210.88)	2.30 (0.02 to 242.66)
0.69 (0.03 to 16.99)	0.69 (0.00 to 111.93)	**MRAs**	2.08 (0.18 to 23.40)
1.44 (0.18 to 11.68)	1.44 (0.02 to 126.06)	2.08 (0.18 to 23.40)	**Active Control**

Note: Network meta-analysis results for outcome from RCTs only (upper triangle: green) and RCTs/non-randomised studies (lower triangle: blue) For RCT only, SMDs >0 or ORs <1 indicate that the treatment specified in the row is more efficacious than that in the column. For RCTs/non-randomised studies results, SMDs >0 or ORs <1 indicate that the treatment specified in the column is more efficacious than that in the row column. Bold underlined results indicate statistical significance. To obtain SMDs for comparisons in the opposite direction, positive values should be converted into negative values, and vice versa. Similarly, reciprocals should be taken to obtain ORs for comparisons in the opposite direction.

Abbreviations: ACEIs, angiotensin-converting enzyme inhibitors; ARBs, angiotensin II receptor blockers; CIs, confidence intervals; MRAs, mineralocorticoid receptor antagonists; NA, not applicable; ORs, odds ratios; RCTs, randomised-controlled trials; SMDs, standardised mean differences.

Table S13. Sensitivity Analysis: Excluding Crossover Studies (Finding from RCTs and Non-Randomised Studies) (Continued)
(I) Dry Cough

Effect Estimate: ORs (95% CIs)		
**ACEIs**	0.64 (0.03 to 12.43)	**17.83 (1.22 to 261.44)**
0.64 (0.01 to 41.96)	**ARBs**	27.73 (0.51 to 1510.88)
5.38 (0.34 to 84.02)	8.36 (0.06 to 1242.42)	**Active Control**

Note: Network meta-analysis results for outcome from RCTs only (upper triangle: green) and RCTs/non-randomised studies (lower triangle: blue) For RCT only, SMDs >0 or ORs <1 indicate that the treatment specified in the row is more efficacious than that in the column. For RCTs/non-randomised studies results, SMDs >0 or ORs <1 indicate that the treatment specified in the column is more efficacious than that in the row column. Bold underlined results indicate statistical significance. To obtain SMDs for comparisons in the opposite direction, positive values should be converted into negative values, and vice versa. Similarly, reciprocals should be taken to obtain ORs for comparisons in the opposite direction.

Abbreviations: ACEIs, angiotensin-converting enzyme inhibitors; ARBs, angiotensin II receptor blockers; CIs, confidence intervals; NA, not applicable; ORs, odds ratios; RCTs, randomised-controlled trials; SMDs, standardised mean differences.

Table S13. Sensitivity Analysis: Excluding Crossover Studies (Finding from RCTs and Non-Randomised Studies) (Continued)
(J) Hypotension

Effect Estimate: ORs (95% CIs)		
**ACEIs**	NA	NA
0.46 (0.01 to 29.13)	**MRAs**	0.80 (0.00 to 1155.18)
0.58 (0.02 to 17.98)	1.25 (0.12 to 12.64)	**Active Control**

Note: Network meta-analysis results for outcome from RCTs only (upper triangle: green) and RCTs/non-randomised studies (lower triangle: blue) For RCT only, SMDs >0 or ORs <1 indicate that the treatment specified in the row is more efficacious than that in the column. For RCTs/non-randomised studies results, SMDs >0 or ORs <1 indicate that the treatment specified in the column is more efficacious than that in the row column. Bold underlined results indicate statistical significance. To obtain SMDs for comparisons in the opposite direction, positive values should be converted into negative values, and vice versa. Similarly, reciprocals should be taken to obtain ORs for comparisons in the opposite direction.

Abbreviations: ACEIs, angiotensin-converting enzyme inhibitors; ARBs, angiotensin II receptor blockers; CIs, confidence intervals; MRAs, mineralocorticoid receptor antagonists; NA, not applicable; ORs, odds ratios; RCTs, randomised-controlled trials; SMDs, standardised mean differences.

Table S13. Sensitivity Analysis: Excluding Crossover Studies (Finding from RCTs and Non-Randomised Studies) (Continued)

(K) Dizziness

**Effect Estimate: ORs (95% CIs)**		
**ACEIs**	NA	0.15 (0.00 to 5.67)
NA	**MRAs**	NA
0.15 (0.00 to 5.67)	NA	**Active Control**

Note: Network meta-analysis results for outcome from RCTs only (upper triangle: green) and RCTs/non-randomised studies (lower triangle: blue) For RCT only, SMDs >0 or ORs <1 indicate that the treatment specified in the row is more efficacious than that in the column. For RCTs/non-randomised studies results, SMDs >0 or ORs <1 indicate that the treatment specified in the column is more efficacious than that in the row column. Bold underlined results indicate statistical significance. To obtain SMDs for comparisons in the opposite direction, positive values should be converted into negative values, and vice versa. Similarly, reciprocals should be taken to obtain ORs for comparisons in the opposite direction.

Abbreviations: ACEIs, angiotensin-converting enzyme inhibitors; ARBs, angiotensin II receptor blockers; CIs, confidence intervals; MRAs, mineralocorticoid receptor antagonists; NA, not applicable; ORs, odds ratios; RCTs, randomised-controlled trials; SMDs, standardised mean differences.

Table S13. Sensitivity Analysis: Excluding Crossover Studies (Finding from RCTs and Non-Randomised Studies) (Continued)
(L) Peritonitis

Effect Estimate: ORs (95% CIs)			
**ACEIs**	0.98 (0.35 to 2.76)	0.86 (0.28 to 2.66)	0.82 (0.36 to 1.89)
0.94 (0.34 to 2.65)	**ARBs**	0.87 (0.22 to 3.43)	0.84 (0.27 to 2.59)
0.77 (0.26 to 2.31)	0.81 (0.21 to 3.15)	**MRAs**	0.96 (0.44 to 2.06)
0.73 (0.33 to 1.61)	0.78 (0.25 to 2.38)	0.96 (0.44 to 2.06)	**Active Control**

Note: Network meta-analysis results for outcome from RCTs only (upper triangle: green) and RCTs/non-randomised studies (lower triangle: blue) For RCT only, SMDs >0 or ORs <1 indicate that the treatment specified in the row is more efficacious than that in the column. For RCTs/non-randomised studies results, SMDs >0 or ORs <1 indicate that the treatment specified in the column is more efficacious than that in the row column. Bold underlined results indicate statistical significance. To obtain SMDs for comparisons in the opposite direction, positive values should be converted into negative values, and vice versa. Similarly, reciprocals should be taken to obtain ORs for comparisons in the opposite direction.

Abbreviations: ACEIs, angiotensin-converting enzyme inhibitors; ARBs, angiotensin II receptor blockers; CIs, confidence intervals; MRAs, mineralocorticoid receptor antagonists; NA, not applicable; ORs, odds ratios; RCTs, randomised-controlled trials; SMDs, standardised mean differences.

Table S13. Sensitivity Analysis: Excluding Crossover Studies (Finding from RCTs and Non-Randomised Studies) (Continued)

(M) Hospitalisation

**Effect Estimate: ORs (95% CIs)**		
**ACEIs**	0.82 (0.24 to 2.81)	1.14 (0.41 to 3.17)
0.82 (0.24 to 2.81)	**ARBs**	1.39 (0.28 to 6.88)
1.14 (0.41 to 3.17)	1.39 (0.28 to 6.88)	**Active Control**

Note: Network meta-analysis results for outcome from RCTs only (upper triangle: green) and RCTs/non-randomised studies (lower triangle: blue) For RCT only, SMDs >0 or ORs <1 indicate that the treatment specified in the row is more efficacious than that in the column. For RCTs/non-randomised studies results, SMDs >0 or ORs <1 indicate that the treatment specified in the column is more efficacious than that in the row column. Bold underlined results indicate statistical significance. To obtain SMDs for comparisons in the opposite direction, positive values should be converted into negative values, and vice versa. Similarly, reciprocals should be taken to obtain ORs for comparisons in the opposite direction.

Abbreviations: ACEIs, angiotensin-converting enzyme inhibitors; ARBs, angiotensin II receptor blockers; CIs, confidence intervals; NA, not applicable; ORs, odds ratios; RCTs, randomised-controlled trials; SMDs, standardised mean differences.

Table S14. Sensitivity Analysis: Excluding Studies with Mixed Treatment Intervention (Finding from RCTs and Non-Randomised Studies)

(A) Residual Glomerular Filtration Rate (mL/min/1.73 m^2)

**Effect Estimate: SMDs (95% CIs)**		
**ACEIs**	-0.10 (-0.68 to 0.49)	0.52 (-0.07 1.11)
-0.10 (-0.68 to 0.49)	**ARBs**	**0.62 (0.10 to 1.14)**
0.52 (-0.07 to 1.11)	**0.62 (0.10 to 1.14)**	**Active Control**

Note: Network meta-analysis results for outcome from RCTs only (upper triangle: green) and RCTs/non-randomised studies (lower triangle: blue) For RCT only, SMDs >0 or ORs <1 indicate that the treatment specified in the row is more efficacious than that in the column. For RCTs/non-randomised studies results, SMDs >0 or ORs <1 indicate that the treatment specified in the column is more efficacious than that in the row column. Bold underlined results indicate statistical significance. To obtain SMDs for comparisons in the opposite direction, positive values should be converted into negative values, and vice versa. Similarly, reciprocals should be taken to obtain ORs for comparisons in the opposite direction.

Abbreviations: ACEIs, angiotensin-converting enzyme inhibitors; ARBs, angiotensin II receptor blockers; CIs, confidence intervals; NA, not applicable; ORs, odds ratios; RCTs, randomised-controlled trials; SMDs, standardised mean differences.

Table S14. Sensitivity Analysis: Excluding Studies with Mixed Treatment Intervention (Finding from RCTs and Non-Randomised Studies) (Continued)
(B) Incidence of Anuria

Effect Estimate: ORs (95% CIs)		
**ACEIs**	0.81 (0.48 to 1.34)	**0.62 (0.41 to 0.95)**
0.85 (0.53 to 1.36)	**ARBs**	0.77 (0.46 to 1.29)
**0.69 (0.57 to 0.83)**	0.81 (0.51 to 1.31)	**Active Control**

Note: Network meta-analysis results for outcome from RCTs only (upper triangle: green) and RCTs/non-randomised studies (lower triangle: blue) For RCT only, SMDs >0 or ORs <1 indicate that the treatment specified in the row is more efficacious than that in the column. For RCTs/non-randomised studies results, SMDs >0 or ORs <1 indicate that the treatment specified in the column is more efficacious than that in the row column. Bold underlined results indicate statistical significance. To obtain SMDs for comparisons in the opposite direction, positive values should be converted into negative values, and vice versa. Similarly, reciprocals should be taken to obtain ORs for comparisons in the opposite direction.

Abbreviations: ACEIs, angiotensin-converting enzyme inhibitors; ARBs, angiotensin II receptor blockers; CIs, confidence intervals; NA, not applicable; ORs, odds ratios; RCTs, randomised-controlled trials; SMDs, standardised mean differences.

Table S14. Sensitivity Analysis: Excluding Studies with Mixed Treatment Intervention (Finding from RCTs and Non-Randomised Studies) (Continued)

(C) Dialysate-to-Plasma Creatinine Ratio

**Effect Estimate: SMDs (95% CIs)**	
**ARBs**	NA
-0.04 (-0.57 to 0.48)	**Active Control**

Note: Network meta-analysis results for outcome from RCTs only (upper triangle: green) and RCTs/non-randomised studies (lower triangle: blue) For RCT only, SMDs >0 or ORs <1 indicate that the treatment specified in the row is more efficacious than that in the column. For RCTs/non-randomised studies results, SMDs >0 or ORs <1 indicate that the treatment specified in the column is more efficacious than that in the row column. Bold underlined results indicate statistical significance. To obtain SMDs for comparisons in the opposite direction, positive values should be converted into negative values, and vice versa. Similarly, reciprocals should be taken to obtain ORs for comparisons in the opposite direction.

Abbreviations: ARBs, angiotensin II receptor blockers; CIs, confidence intervals; NA, not applicable; ORs, odds ratios; RCTs, randomised-controlled trials; SMDs, standardised mean differences.

Table S15. Meta-Regression for Pairwise Analysis: Residual Glomerular Filtration Rate (Finding from RCTs and Non-Randomised Studies)

**Covariate**	**Effect Estimate SMDs**					
	**ACEIs[a]**			**ARBs[a]**		
	**No. of Studies (N)**	**Regression Equation ß Coefficient (95% CI)[b]**	***P* Value**	**No. of Studies (N)**	**Regression Equation ß Coefficient (95% CI)[b]**	***P* Value**
**Study-Level Characteristics**						
Sample size (≤50 vs. >50)	NA	NA	NA	3 (104)	NA	NA
Asian vs. Non-Asian countries	NA	NA	NA	3 (104)	NA	NA
Study design (RCTs vs. Non-RCTs	NA	NA	NA	3 (104)	NA	NA
**Baseline Participant-Level Characteristics**						
Age (mean, per 1 year)	NA	NA	NA	3 (104)	0.04 (-0.38 to 0.45)	0.470
Female (per %)	NA	NA	NA	3 (104)	0.07 (-0.35 to 0.48)	0.292
Diabetes mellitus (per %)	NA	NA	NA	NA	NA	NA
rGFR (mean, per mL/min/1.73 m^2)	NA	NA	NA	3 (104)	-0.36 (-23.48 to 22.77)	0.877
Systolic BP (mean, per mmHg)	NA	NA	NA	3 (104)	0.03 (-0.16 to 0.22)	0.273
Diastolic BP (mean, per mmHg)	NA	NA	NA	3 (104)	-0.00 (-0.52 to 0.52)	0.926
CAPD (per %)	NA	NA	NA	3 (104)	0.82 (-0.60 to 2.24)	0.131

[a]Summary of treatment specified compared with active control.
[b]β coefficient for natural logarithm of effect size for each variable of interest reflecting unit change.
Abbreviations: ACEIs, angiotensin-converting enzyme inhibitors; ARBs, angiotensin II receptor blockers; BP, blood pressure; CAPD, continuous ambulatory peritoneal dialysis; CIs, confidence intervals; NA, not applicable; RCTs, randomised-controlled trials; rGFR, residual glomerular filtration rate; SMDs, standardised mean differences.

Table S15. Meta-Regression for Pairwise Analysis: Urine Volume (Finding from RCTs and Non-Randomised Studies) (Continued)

Covariate	Effect Estimate SMDs					
	ACEIs[a]			ARBs[a]		
	No. of Studies (N)	Regression Equation ß Coefficient (95% CI)[b]	*P* Value	No. of Studies (N)	Regression Equation ß Coefficient (95% CI)[b]	*P* Value
**Study-Level Characteristics**						
Sample size (≤50 vs. >50)	NA	NA	NA	4 (120)	NA	NA
Asian vs. Non-Asian countries	NA	NA	NA	4 (120)	NA	NA
Study design (RCTs vs. Non-RCTs	NA	NA	NA	4 (120)	-1.26 (-8.84 to 6.32)	0.548
**Baseline Participant-Level Characteristics**						
Age (mean, per 1 year)	NA	NA	NA	4 (120)	0.04 (-0.27 to 0.34)	0.644
Female (per %)	NA	NA	NA	4 (120)	0.05 (-0.40 to 0.50)	0.664
Diabetes mellitus (per %)	NA	NA	NA	3 (86)	0.06 (-0.83 to 0.96)	0.524
rGFR (mean, per mL/min/1.73 m^2)	NA	NA	NA	3 (112)	-3.42 (-48.87 to 42.03)	0.514
Systolic BP (mean, per mmHg)	NA	NA	NA	3 (112)	0.06 (-0.73 to 0.86)	0.485
Diastolic BP (mean, per mmHg)	NA	NA	NA	3 (112)	-0.07 (-1.15 to 1.01)	0.562
CAPD (per %)	NA	NA	NA	4 (120)	0.05 (-0.25 to 0.35)	0.548

[a]Summary of treatment specified compared with active control.
[b]β coefficient for natural logarithm of effect size for each variable of interest reflecting unit change.
Abbreviations: ACEIs, angiotensin-converting enzyme inhibitors; ARBs, angiotensin II receptor blockers; BP, blood pressure; CAPD, continuous ambulatory peritoneal dialysis; CIs, confidence intervals; NA, not applicable; RCTs, randomised-controlled trials; SMDs, standardised mean differences.

Table S15. Meta-Regression for Pairwise Analysis: Incidence of Anuria (Finding from RCTs and Non-Randomised Studies) (Continued)

**Covariate**	**Effect Estimate ORs**					
	**ACEIs[a]**			**ARBs[a]**		
	**No. of Studies (N)**	**Regression Equation ß Coefficient (95% CI)[b]**	***P* Value**	**No. of Studies (N)**	**Regression Equation ß Coefficient (95% CI)[b]**	***P* Value**
**Study-Level Characteristics**						
Sample size (≤50 vs. >50)	4 (1,265)	2.41 (0.14 to 42.72)	0.319	NA	NA	NA
Asian vs. Non-Asian countries	4 (1,265)	1.22 (0.37 to 4.02)	0.552	NA	NA	NA
Study design (RCTs vs. Non-RCTs	4 (1,265)	1.22 (0.37 to 4.02)	0.552	NA	NA	NA
**Baseline Participant-Level Characteristics**						
Age (mean, per 1 year)	4 (1,265)	1.03 (0.87 to 1.22)	0.562	NA	NA	NA
Female (per %)	4 (1,265)	1.02 (0.95 to 1.09)	0.385	NA	NA	NA
Diabetes mellitus (per %)	4 (1,265)	1.00 (0.94 to 1.07)	0.809	NA	NA	NA
rGFR (mean, per mL/min/1.73 m^2)	3 (1,238)	1.02 (0.46 to 2.23)	0.824	NA	NA	NA
Systolic BP (mean, per mmHg)	3 (813)	1.02 (0.73 to 1.41)	0.644	NA	NA	NA
Diastolic BP (mean, per mmHg)	3 (813)	1.05 (0.49 to 2.25)	0.540	NA	NA	NA
CAPD (per %)	4 (1,265)	1.00 (0.96 to 1.03)	0.660	NA	NA	NA

[a]Summary of treatment specified compared with active control.

[b]β coefficient for natural logarithm of effect size for each variable of interest reflecting unit change.

Abbreviations: ACEIs, angiotensin-converting enzyme inhibitors; ARBs, angiotensin II receptor blockers; BP, blood pressure; CAPD, continuous ambulatory peritoneal dialysis; CIs, confidence intervals; NA, not applicable; ORs, odds ratios; RCTs, randomised-controlled trials.

Table S15. Meta-Regression for Pairwise Analysis: Acceptability of Treatment (Finding from RCTs and Non-Randomised Studies) (Continued)

**Covariate**	**Effect Estimate ORs**					
	**ACEIs[a]**			**ARBs[a]**		
	**No. of Studies (N)**	**Regression Equation ß Coefficient (95% CI)[b]**	***P* Value**	**No. of Studies (N)**	**Regression Equation ß Coefficient (95% CI)[b]**	***P* Value**
**Study-Level Characteristics**						
Sample size (≤50 vs. >50)	4 (185)	7.37 (0.09 to 587.32)	0.189	6 (151)	NA	NA
Asian vs. Non-Asian countries	4 (185)	0.19 (0.00 to 25.72)	0.283	6 (151)	0.92 (0.00 to 354.37)	0.970
Study design (RCTs vs. Non-RCTs	4 (185)	0.16 (0.00, 24.62)	0.255	6 (151)	0.89 (0.02 to 48.02)	0.942
**Baseline Participant-Level Characteristics**						
Age (mean, per 1 year)	4 (185)	1.14 (0.91 to 1.42)	0.127	6 (151)	0.99 (0.82 to 1.20)	0.882
Female (per %)	4 (185)	0.95 (0.62 to 1.43)	0.619	6 (151)	1.00 (0.80 to 1.25)	0.976
Diabetes mellitus (per %)	3 (176)	1.06 (0.59 to 1.91)	0.441	5 (117)	1.00 (0.82 to 1.21)	1.000
rGFR (mean, per mL/min/1.73 m^2)	NA	NA	NA	4 (123)	1.07 (0.10 to 11.73)	0.910
Systolic BP (mean, per mmHg)	3 (176)	1.07 (0.68 to 1.67)	0.313	4 (136)	1.02 (0.79 to 1.32)	0.746
Diastolic BP (mean, per mmHg)	3 (176)	1.23 (0.46 to 3.24)	0.226	4 (136)	1.05 (0.73 to 1.52)	0.623
CAPD (per %)	4 (185)	NA	NA	6 (151)	1.00 (0.79 to 1.28)	0.971

[a]Summary of treatment specified compared with active control.

[b]β coefficient for natural logarithm of effect size for each variable of interest reflecting unit change.

Abbreviations: ACEIs, angiotensin-converting enzyme inhibitors; ARBs, angiotensin II receptor blockers; BP, blood pressure; CAPD, continuous ambulatory peritoneal dialysis; CIs, confidence intervals; NA, not applicable; ORs, odds ratios; RCTs, randomised-controlled trials.

Table S16. Evaluation of Inconsistency: Loop-Specific Approach

Loop	IF (95% CI)	*P* Value	Loop Heterogeneity (τ^2)
**Primary Outcomes**			
Residual glomerular Filtration Rate			
ACEIs-ARBs-Active Control	0.78 (0.00-1.96)	0.198	0.146
Urine Volume			
No triangular or quadratic loops	NA	NA	NA
Incidence of Anuria			
ACEIs-ARBs-Active Control	0.17 (0.00-1.12)	0.725	<0.001
D/P Cr Ratio			
No triangular or quadratic loops	NA	NA	NA
Acceptability of Treatment			
ACEIs-ARBs-Active Control	0.18 (0.00-3.35)	0.913	<0.001
**Secondary Outcomes**			
4-hour Ultrafiltration Volume by PET			
No triangular or quadratic loops	NA	NA	NA
Daily Ultrafiltration Volume			
No triangular or quadratic loops	NA	NA	NA
Hyperkalaemia			
ACEIs-ARBs-Active Control	0.55 (0.00-5.29)	0.819	<0.001
Dry Cough			
No triangular or quadratic loops	NA	NA	NA
Hypotension			
No triangular or quadratic loops	NA	NA	NA
Dizziness			
No triangular or quadratic loops	NA	NA	NA
Peritonitis			
ACEIs-ARBs-Active Control	0.47 (0.00-2.71)	0.685	<0.001
Hospitalisation			
No triangular or quadratic loops	NA	NA	NA

Abbreviations: ACEIs, angiotensin-converting enzyme inhibitors; ARBs, angiotensin-II receptor blockers; CI, confidence interval; D/P Cr, dialysate-to-plasma creatinine; IF, inconsistency factor; PET, peritoneal equilibration test.

Table S17. Evaluation of Inconsistency: Node-Splitting Model

**Outcomes**	***P* Value for Side-Splitting**[a]		
	**Active Control-ACEIs**	**Active Control-ARBs**	**ACEIs-ARBs**
**Primary Outcomes**			
Residual glomerular filtration rate	NA	NA	NA
Urine volume	NA	NA	NA
Incidence anuria	0.725	0.725	0.725
Dialysate-to-plasma creatinine ratio	NA	NA	NA
Acceptability of treatment	0.920	0.920	0.920
**Secondary Outcomes**			
4-hour ultrafiltration volume	NA	NA	NA
Daily ultrafiltration volume	NA	NA	NA
Hyperkalaemia	0.819	0.819	0.819
Dry cough	NA	NA	NA
Hypotension	NA	NA	NA
Dizziness	NA	NA	NA
Peritonitis	0.685	0.685	0.685
Hospitalisation	NA	NA	NA

[a]$P<0.05$ indicates that there is inconsistency between direct and indirect evidence for this comparison.
Abbreviations: ACEIs, angiotensin-converting enzyme inhibitors; ARBs, angiotensin II receptor blockers; NA, not applicable.

Table S18. Evaluation of Inconsistency: Design-by-Treatment Interaction Model

**Outcomes**	**Chi-Square**	***P* Value for Test of Global Inconsitency**[a]
**Primary Outcomes**		
Residual glomerular filtration rate (mL/min/1.73 m^2)	1.99	0.158
Urine volume (mL/day)	0.03	0.858
Incidence of anuria	0.12	0.724
Dialysate-to-plasma creatinine ratio	31.15	<0.001
Acceptability of treatment	0.01	0.920
**Secondary Outcomes**		
4-hour ultrafiltration volume by (mL/4 hour)	3.98	0.046
Daily ultrafiltration volume (mL/day)	0.15	0.697
Hyperkalaemia	0.05	0.819
Dry cough	0.42	0.516
Hypotension	NA	NA
Dizziness	NA	NA
Peritonitis	0.17	0.684
Hospitalisation	0.10	0.754

[a]*P*>0.05 indicates that data fits consistency model, P<0.05 indicates it fits inconsistency model better.
Abbreviations: NA, not applicable.

Table S19. Evaluation of the Strength of the Body of Evidence: Finding from RCTs and Non-Randomised Studies

**Treatment Comparison**	**Direct Evidence**		**Indirect Evidence**		**Summary Network Effect Estimate (95% CI)**	**Summary of SOE**
	**Effect Estimate (95% CI)**	**Quality of Evidence**	**Effect Estimate (95% CI)**	**Quality of Evidence**		
**Primary Outcomes**						
Change in residual glomerular filtration rate, mL/min/1.73/ m²						
ACEIs vs. active control	SMD 0.17 (-0.80 to 1.15)	Insufficient[a,c]	NA	NA	SMD 0.55 (0.06 to 1.04)[e]	Low ●○○○
ARBs vs. active control	SMD 0.82 (0.17 to 1.47)	Low[a,d]	NA	NA	SMD 0.62 (0.19 to 1.04)[e]	Low ●○○○
Mixed ACEIs/ARBs vs. active control	SMD 0.41 (0.25 to 0.57)	Insufficient[a,b,f]	NA	NA	SMD 0.45 (0.03 to 0.86)[e]	Insufficient[g] ○○○○
ACEIs vs. ARBs	SMD -0.19 (-0.63 to 0.25)	Low[a,d]	NA	NA	SMD -0.07 (-0.55 to 0.42)[e]	Low ●○○○
ACEIs vs. mixed ACEIs/ARBs	NA	NA	NA	NA	SMD 0.10 (-0.55 to 0.75)[e]	Insufficient[g] ○○○○
ARBs vs. mixed ACEIs/ARBs	NA	NA	NA	NA	SMD 1.18 (0.65 to 2.15)[e]	Insufficient[g] ○○○○
Change in urine volume, mL/day						
ACEIs vs. active control	SMD 0.20 (-0.45 to 0.86)	Insufficient[a,b,c,d]	NA	NA	SMD 0.20 (-2.39 to 2.80)[e]	Insufficient[g] ○○○○
ARBs vs. active control	SMD 1.07 (-0.07 to 2.21)	Low[a,d]	NA	NA	SMD 1.08 (-0.25 to 2.41)[e]	Low ●○○○
MRAs vs. active control	SMD -0.24 (-0.86 to 0.39)	Insufficient[a,c,d]	NA	NA	SMD -0.24 (-2.83 to 2.35)[e]	Insufficient[g] ○○○○

[a]Study limitations (risk of bias); [b]Indirectness; [c]Inconsistency; [d]Imprecision; [e]reporting bias; [f]Other issues (plausible confounding that would decreased observed effect); [g]Indirectness because of intransitivity; [h]Severe imprecision

Abbreviations: ACEIs, angiotensin-converting enzyme inhibitors; ARBs, angiotensin II receptor blockers; CIs, confidence intervals; MRAs, mineralocorticoid receptor antagonists; NA, not applicable; ORs, odds ratios; RCTs, randomised-controlled trials; SMDs, standardised mean differences; SOE, strength of evidence.

Table S19. Evaluation of the Strength of the Body of Evidence: Finding from RCTs and Non-Randomised Studies (Continued)

Treatment Comparison	Direct Evidence		Indirect Evidence		Summary Network Effect Estimate (95% CI)	Summary of SOE
	Effect Estimate (95% CI)	Quality of Evidence	Effect Estimate (95% CI)	Quality of Evidence		
**Primary Outcomes**						
Change in urine volume, mL/day (continued)						
ACEIs vs. ARBs	NA	NA	NA	NA	SMD -0.88 (-3.79 to 2.04)[e]	Insufficient[g,h] ○○○○
ACEIs vs. MRAs	NA	NA	NA	NA	SMD 0.44 (-3.23 to 4.11)[e]	Insufficient[g,h] ○○○○
ARBs vs. MRAs	NA	NA	NA	NA	SMD 1.32 (-1.59 to 4.33)[e]	Insufficient[g,h] ○○○○
Incidence of anuria						
ACEIs vs. active control	OR 0.69 (0.57 to 0.83)	Low[a,f]	OR 0.81 (0.32 to 2.07)	Low[a,d]	OR 0.69 (0.57 to 0.83)	Low ●○○○
ARBs vs. active control	OR 0.89 (0.45 to 1.73)	Low[a,d]	OR 0.75 (0.38 to 1.47)	Low[a,d]	OR 0.81 (0.51 to 1.31)	Low ●○○○
Mixed ACEIs/ARBs vs. active control	OR 0.88 (0.75 to 1.03)	Insufficient[a,c,f]	NA	NA	OR 0.88 (0.75 to 1.03)	Insufficient[g] ○○○○
ACEIs vs. ARBs	OR 1.09 (0.57 to 2.08)	Insufficient[a,c,d]	OR 1.29 (0.64 to 1.29)	Insufficient[a,d]	OR 0.85 (0.53 to 1.36)	Insufficient[g] ○○○○
ACEIs vs. mixed ACEIs/ARBs	NA	NA	NA	NA	OR 0.78 (0.61 to 1.00)	Insufficient[g] ○○○○
ARBs vs. mixed ACEIs/ARBs	NA	NA	NA	NA	OR 0.92 (0.56 to 1.52)	Insufficient[g] ○○○○

[a]Study limitations (risk of bias); [b]Indirectness; [c]Inconsistency; [d]Imprecision; [e]reporting bias; [f]Other issues (plausible confounding that would decreased observed effect); [g]Indirectness because of intransitivity; [h]Severe imprecision

Abbreviations: ACEIs, angiotensin-converting enzyme inhibitors; ARBs, angiotensin II receptor blockers; CIs, confidence intervals; MRAs, mineralocorticoid receptor antagonists; NA, not applicable; ORs, odds ratios; RCTs, randomised-controlled trials; SMDs, standardised mean differences; SOE, strength of evidence.

Table S19. Evaluation of the Strength of the Body of Evidence: Finding from RCTs and Non-Randomised Studies (Continued)

Treatment Comparison	Direct Evidence		Indirect Evidence		Summary Network Effect Estimate (95% CI)	Summary of SOE
	Effect Estimate (95% CI)	Quality of Evidence	Effect Estimate (95% CI)	Quality of Evidence		
**Primary Outcomes**						
Change in Dialysate-to-Plasma Creatinine Ratio						
ARBs vs. active control	SMD 0.04 (-0.48 to 0.57)	Insufficient[a,d,f]	NA	NA	SMD 0.04 (-0.48 to 0.57)	Insufficient[g] ○○○○
Mixed ACEIs/ARBs vs. active control	SMD -1.60 (-2.16 to -1.04)	Insufficient[a,c,d,f]	NA	NA	SMD -1.60 (-2.16 to -1.04)	Insufficient[g] ○○○○
ARBs vs. mixed ACEIs/ARBs	NA	NA	NA	NA	SMD 1.64 (0.87 to 2.41)	Insufficient[g] ○○○○
Acceptability of Treatment						
ACEIs vs. active control	OR 0.93 (0.26 to 3.26)	Insufficient[a,c,d]	OR 1.09 (0.04 to 64.87)	Low[a,d]	OR 0.93 (0.37 to 2.37)	Low ●○○○
ARBs vs. active control	OR 1.08 (0.29 to 3.99)	Insufficient[a,c,d]	OR 0.92 (0.04 to 20.49)	Low[a,d]	OR 1.06 (0.29 to 3.84)	Low ●○○○
MRAs vs. active control	OR 1.46 (0.75 to 2.84)	Low[a,d]	NA	NA	OR 1.42 (0.40 to 5.05)	Low ●○○○
ACEIs vs. ARBs	OR 1.00 (0.06 to 16.45)	Low[a,d]	OR 1.19 (0.21 to 6.85)	Low[a,d]	OR 0.88 (0.20 to 3.86)	Low ●○○○
ACEIs vs. MRAs	NA	NA	NA	NA	OR 0.65 (0.14 to 3.16)	Insufficient[g] ○○○○
ARBs vs. MRAs	NA	NA	NA	NA	OR 0.92 (0.56 to 1.52)	Insufficient[g] ○○○○

[a]Study limitations (risk of bias); [b]Indirectness; [c]Inconsistency; [d]Imprecision; [e]reporting bias; [f]Other issues (plausible confounding that would decreased observed effect); [g]Indirectness because of intransitivity; [h]Severe imprecision

Abbreviations: ACEIs, angiotensin-converting enzyme inhibitors; ARBs, angiotensin II receptor blockers; CIs, confidence intervals; MRAs, mineralocorticoid receptor antagonists; NA, not applicable; ORs, odds ratios; RCTs, randomised-controlled trials; SMDs, standardised mean differences; SOE, strength of evidence.

Table S19. Evaluation of the Strength of the Body of Evidence: Finding from RCTs and Non-Randomised Studies (Continued)

**Treatment Comparison**	**Direct Evidence**		**Indirect Evidence**		**Summary Network Effect Estimate (95% CI)**	**Summary of SOE**
	**Effect Estimate (95% CI)**	**Quality of Evidence**	**Effect Estimate (95% CI)**	**Quality of Evidence**		
**Secondary Outcomes**						
Change in 4-hour ultrafiltration volume (mL/4 hour)						
ACEIs vs. active control	SMD -0.36 (-1.40 to 0.68)	Insufficient[a,c,d]	NA	NA	SMD -0.36 (-1.41 to 0.70)	Insufficient[g] ○○○○
ARBs vs. active control	SMD -0.19 (-1.24 to 0.86)	Insufficient[a,c,d,f]	NA	NA	SMD -0.19 (-1.81 to 1.42)	Insufficient[g] ○○○○
Mixed ACEIs/ARBs vs. active control	SMD 1.36 (0.82 to 1.90)	Insufficient[a,c,d,f]	NA	NA	SMD 1.36 (0.02 to 2.70)	Insufficient[g] ○○○○
ACEIs vs. ARBs	NA	NA	NA	NA	SMD -0.16 (-2.09 to 1.76)	Insufficient[g] ○○○○
ACEIs vs. mixed ACEIs/ARBs	NA	NA	NA	NA	SMD -1.72 (-3.42 to -0.02)	Insufficient[g] ○○○○
ARBs vs. mixed ACEIs/ARBs	NA	NA	NA	NA	SMD -1.56 (-3.65 to 0.54)	Insufficient[g] ○○○○
Daily ultrafiltration volume (mL/day)						
ACEIs vs. active control	SMD 0.74 (-0.35 to 1.83)	Low[a,d]	NA	NA	SMD 0.74 (-0.35 to 1.83)	Insufficient[g] ○○○○
ARBs vs. active control	SMD -0.34 (-1.33 to 0.65)	Insufficient[a,c,d]	NA	NA	SMD -0.34 (-2.06 to 1.38)	Insufficient[g] ○○○○
ACEIs vs. ARBs	NA	NA	NA	NA	SMD 1.08 (-0.95 to 3.12)	Insufficient[g] ○○○○

[a]Study limitations (risk of bias); [b]Indirectness; [c]Inconsistency; [d]Imprecision; [e]reporting bias; [f]Other issues (plausible confounding that would decreased observed effect); [g]Indirectness because of intransitivity; [h]Severe imprecision

Abbreviations: ACEIs, angiotensin-converting enzyme inhibitors; ARBs, angiotensin II receptor blockers; CIs, confidence intervals; MRAs, mineralocorticoid receptor antagonists; NA, not applicable; ORs, odds ratios; RCTs, randomised-controlled trials; SMDs, standardised mean differences; SOE, strength of evidence.

Table S19. Evaluation of the Strength of the Body of Evidence: Finding from RCTs and Non-Randomised Studies (Continued)

Treatment Comparison	Direct Evidence		Indirect Evidence		Summary Network Effect Estimate (95% CI)	Summary of SOE
	Effect Estimate (95% CI)	Quality of Evidence	Effect Estimate (95% CI)	Quality of Evidence		
**Secondary Outcomes**						
Hyperkalaemia						
ACEIs vs. active control	OR 1.44 (0.18 to 11.69)	Insufficient[a,c,d]	OR 0.83 (0.01 to 58.17)	Low[a,d]	OR 1.29 (0.20 to 8.44)	Insufficient[g,h] ○○○○
ARBs vs. active control	OR 1.00 (0.02 to 58.43)	Insufficient[a,c,d]	OR 1.74 (0.15 to 19.67)	Low[a,d]	OR 1.50 (0.19 to 12.04)	Insufficient[g,h] ○○○○
MRAs vs. active control	OR 2.07 (0.29 to 15.03)	Low[a,d]	NA	NA	OR 2.07 (0.29 to 15.03)	Insufficient[h] ○○○○
ACEIs vs. ARBs	OR 1.21 (0.36 to 4.06)	Low[a,d]	OR 0.70 (0.01 to 68.36)	Low[a,d]	OR 0.86 (0.27 to 2.77)	Low ●○○○
ACEIs vs. MRAs	NA	NA	NA	NA	OR 0.62 (0.04 to 9.56)	Insufficient[g,h] ○○○○
ARBs vs. MRAs	NA	NA	NA	NA	OR 0.21 (0.03 to 1.72)	Insufficient[h] ○○○○
Dry cough						
ACEIs vs. active control	OR 5.38 (0.34 to 85.78)	Insufficient[a,c,d]	NA	NA	OR 5.38 (0.32 to 89.75)	Insufficient[g,h] ○○○○
ARBs vs. active control	NA	NA	NA	NA	OR 1.83 (0.02 to 134.76)	Insufficient[g,h] ○○○○
ACEIs vs. ARBs	OR 0.34 (0.01 to 9.76)	Insufficient[a,c,d]	NA	NA	OR 2.93 (0.11 to 75.62)	Insufficient[g,h] ○○○○

[a]Study limitations (risk of bias); [b]Indirectness; [c]Inconsistency; [d]Imprecision; [e]reporting bias; [f]Other issues (plausible confounding that would decreased observed effect); [g]Indirectness because of intransitivity; [h]Severe imprecision

Abbreviations: ACEIs, angiotensin-converting enzyme inhibitors; ARBs, angiotensin II receptor blockers; CIs, confidence intervals; MRAs, mineralocorticoid receptor antagonists; NA, not applicable; ORs, odds ratios; RCTs, randomised-controlled trials; SMDs, standardised mean differences; SOE, strength of evidence.

Table S19. Evaluation of the Strength of the Body of Evidence: Finding from RCTs and Non-Randomised Studies (Continued)

Treatment Comparison	Direct Evidence		Indirect Evidence		Summary Network Effect Estimate (95% CI)	Summary of SOE
	Effect Estimate (95% CI)	Quality of Evidence	Effect Estimate (95% CI)	Quality of Evidence		
**Secondary Outcomes**						
Hypotension						
ACEIs vs. active control	OR 0.58 (0.03 to 9.74)	Insufficient[a,c,d]	NA	NA	OR 0.58 (0.02 to 17.98)	Insufficient[g,h] ○○○○
MRAs vs. active control	OR 1.25 (0.37 to 4.28)	Insufficient[a,c,d]	NA	NA	OR 1.25 (0.12 to 12.64)	Insufficient[g,h] ○○○○
ACEIs vs. MRAs	NA	NA	NA	NA	OR 0.46 (0.01 to 29.13)	Insufficient[g,h] ○○○○
Dizziness						
ACEIs vs. active control	OR 6.56 (0.31 to 136.92)	Insufficient[a,c,d]	NA	NA	OR 6.56 (0.18 to 243.86)	Insufficient[g,h] ○○○○
MRAs vs. active control	OR 2.11 (0.18 to 25.35)	Insufficient[a,c,d]	NA	NA	OR 2.11 (0.09 to 50.03)	Insufficient[g,h] ○○○○
ACEIs vs. MRAs	NA	NA	NA	NA	OR 3.11 (0.03 to 379.55)	Insufficient[g,h] ○○○○
Gynaecomastia						
MRAs vs. active control	OR 6.40 (1.37 to 29.92)	Insufficient[a,c,d]	NA	NA	OR 6.40 (1.37 to 29.92)	Insufficient[g,h] ○○○○

[a]Study limitations (risk of bias); [b]Indirectness; [c]Inconsistency; [d]Imprecision; [e]reporting bias; [f]Other issues (plausible confounding that would decreased observed effect); [g]Indirectness because of intransitivity; [h]Severe imprecision

Abbreviations: ACEIs, angiotensin-converting enzyme inhibitors; ARBs, angiotensin II receptor blockers; CIs, confidence intervals; MRAs, mineralocorticoid receptor antagonists; NA, not applicable; ORs, odds ratios; RCTs, randomised-controlled trials; SMDs, standardised mean differences; SOE, strength of evidence.

Table S19. Evaluation of the Strength of the Body of Evidence: Finding from RCTs and Non-Randomised Studies (Continued)

**Treatment Comparison**	**Direct Evidence**		**Indirect Evidence**		**Summary Network Effect Estimate (95% CI)**	**Summary of SOE**
	**Effect Estimate (95% CI)**	**Quality of Evidence**	**Effect Estimate (95% CI)**	**Quality of Evidence**		
**Secondary Outcomes**						
Peritonitis						
ACEIs vs. active control	OR 0.79 (0.24 to 1.84)	Insufficient[a,c,d]	OR 0.49 (0.06 to 3.97)	Low[a,d]	OR 0.73 (0.33 to 1.61)	Low ○○○○
ARBs vs. active control	OR 0.60 (0.11 to 3.21)	Insufficient[a,c,d]	OR 0.96 (0.21 to 4.29)	Low[a,d]	OR 0.78 (0.25 to 2.38)	Low[h] ○○○○
MRAs vs. active control	OR 0.96 (0.44 to 2.06)	Insufficient[a,c,d]	NA	NA	OR 0.96 (0.44 to 2.06)	Insufficient ○○○○
ACEIs vs. ARBs	OR 1.22 (0.36 to 4.17)	Insufficient[a,c,d]	OR 0.76 (0.12 to 5.04)	Low[a,d]	OR 0.94 (0.34 to 2.65)	Low[h] ○○○○
ACEIs vs. MRAs	NA	NA	NA	NA	OR 0.77 (0.26 to 2.31)	Insufficient[h] ○○○○
ARBs vs. MRAs	NA	NA	NA	NA	OR 0.81 (0.21 to 3.15)	Insufficient[h] ○○○○
Hospitalisation						
ACEIs vs. active control	OR 1.14 (0.41 to 3.17)	Insufficient[a,c,d]	NA	NA	OR 1.14 (0.41 to 3.17)	Insufficient[h] ○○○○
ARBs vs. active control	NA	NA	NA	NA	OR 1.39 (0.28 to 6.88)	Insufficient[h] ○○○○
ACEIs vs. ARBs	OR 1.17 (0.54 to 2.57)	Insufficient[a,c,d]	NA	NA	OR 0.82 (0.24 to 2.81)	Insufficient[h] ○○○○

[a]Study limitations (risk of bias); [b]Indirectness; [c]Inconsistency; [d]Imprecision; [e]reporting bias; [f]Other issues (plausible confounding that would decreased observed effect); [g]Indirectness because of intransitivity; [h]Severe imprecision

Abbreviations: ACEIs, angiotensin-converting enzyme inhibitors; ARBs, angiotensin II receptor blockers; CIs, confidence intervals; MRAs, mineralocorticoid receptor antagonists; NA, not applicable; ORs, odds ratios; RCTs, randomised-controlled trials; SMDs, standardised mean differences; SOE, strength of evidence.

Figure S1. Network Plot for Secondary Outcomes

(A) 4-Hour Ultrafiltration Volume by Peritoneal Equilibration Test (mL/4 hour)

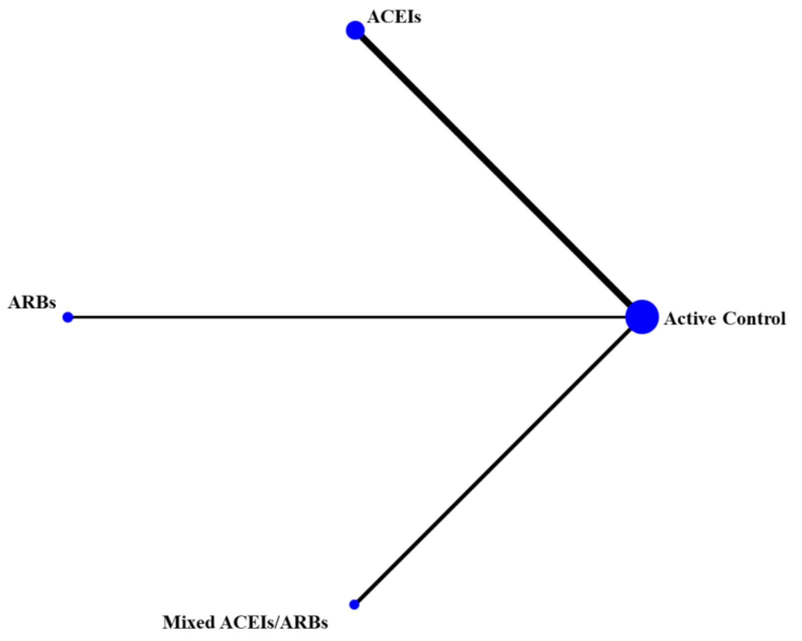

(B) Daily Ultrafiltration Volume (mL/day)

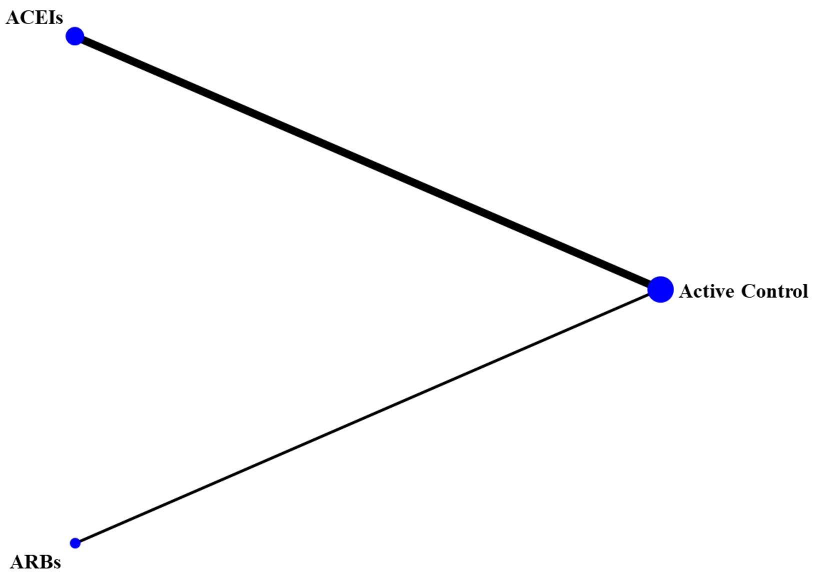

Notes: The circles (nodes) represent the available treatments and the lines (edges) represent the available comparisons. Size nodes and width of edges indicate weighting according to the numbers of studies involved for each treatment and comparison, respectively.
Abbreviations: ACEIs, angiotensin-converting enzyme inhibitors; ARBs, angiotensin II receptor blockers; MRAs, mineralocorticoid receptor antagonists.

Figure S1. Network plot for secondary outcomes (Continued)

(C) Hyperkalaemia

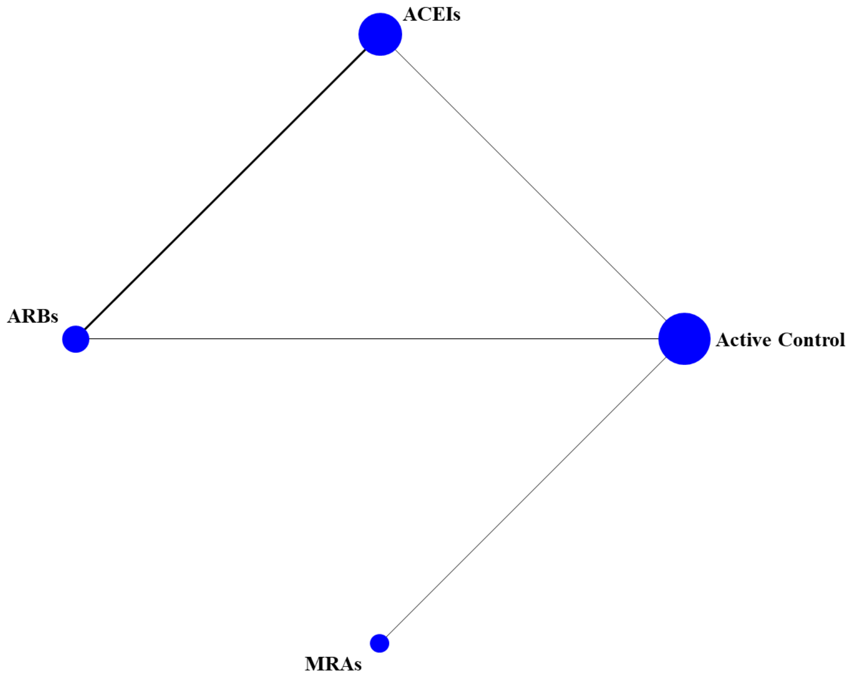

(D) Dry Cough

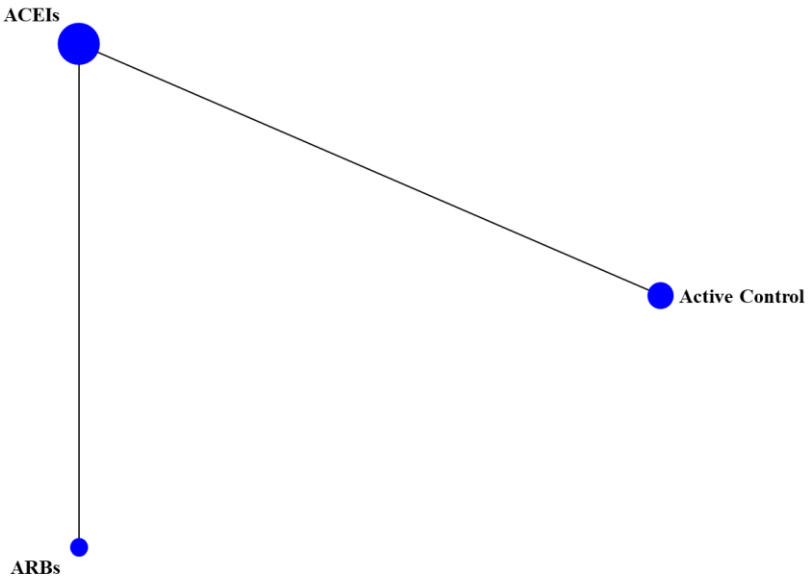

Notes: The circles (nodes) represent the available treatments and the lines (edges) represent the available comparisons. Size nodes and width of edges indicate weighting according to the numbers of studies involved for each treatment and comparison, respectively.

Abbreviations: ACEIs, angiotensin-converting enzyme inhibitors; ARBs, angiotensin II receptor blockers; MRAs, mineralocorticoid receptor antagonists.

Figure S1. Network plot for secondary outcomes (Continued)
(E) Hypotension

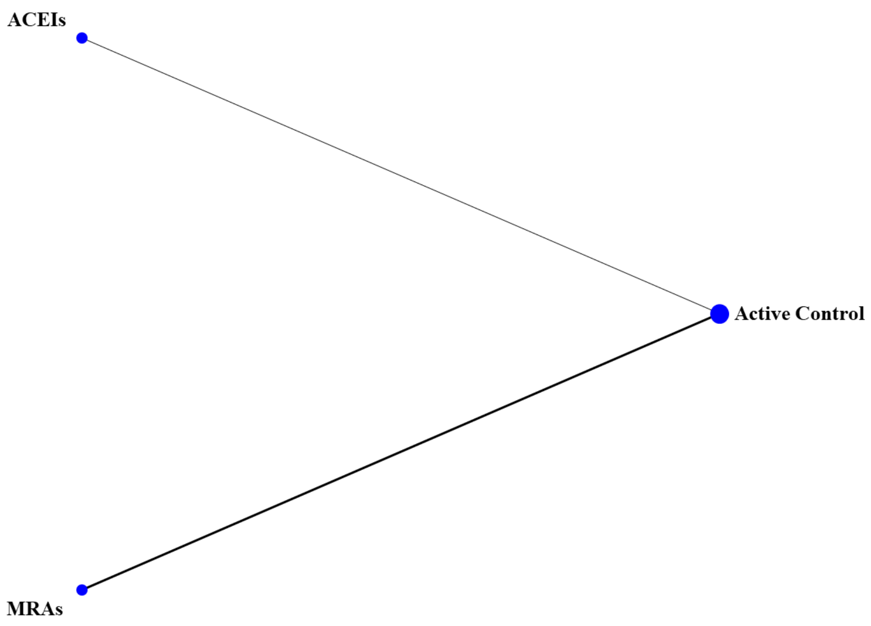

(F) Dizziness

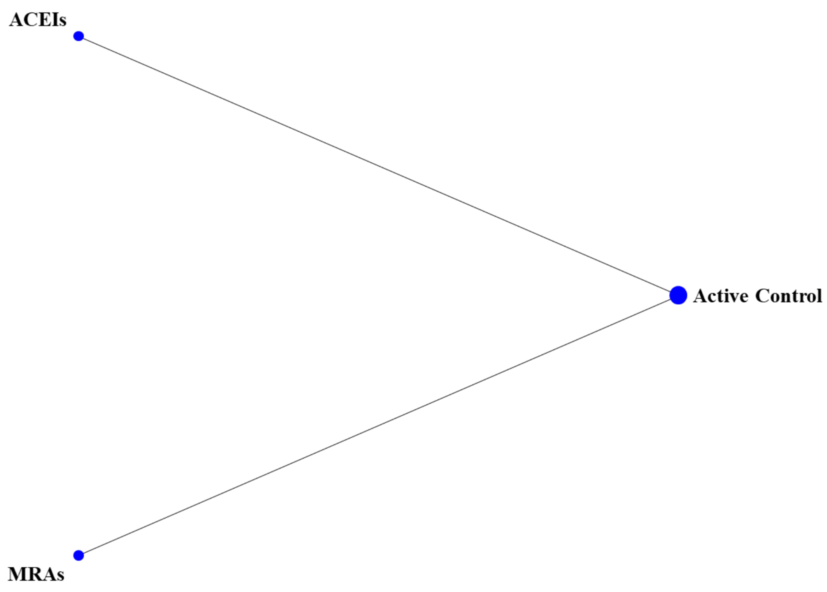

Notes: The circles (nodes) represent the available treatments and the lines (edges) represent the available comparisons. Size nodes and width of edges indicate weighting according to the numbers of studies involved for each treatment and comparison, respectively.
Abbreviations: ACEIs, angiotensin-converting enzyme inhibitors; ARBs, angiotensin II receptor blockers; MRAs, mineralocorticoid receptor antagonists.

Figure S1. Network plot for secondary outcomes (Continued)

(G) Peritonitis

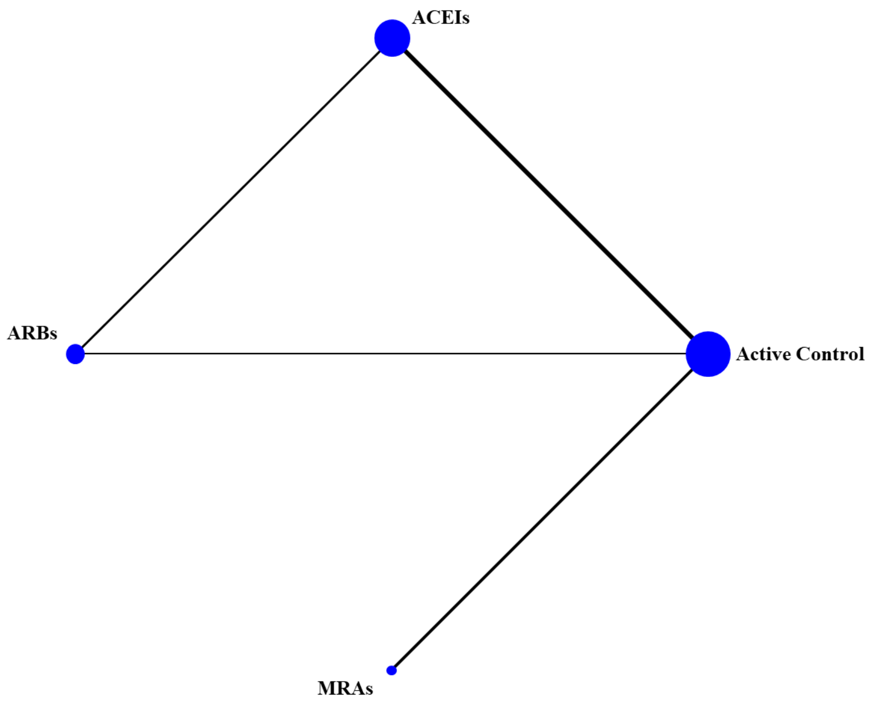

(H) Hospitalisation

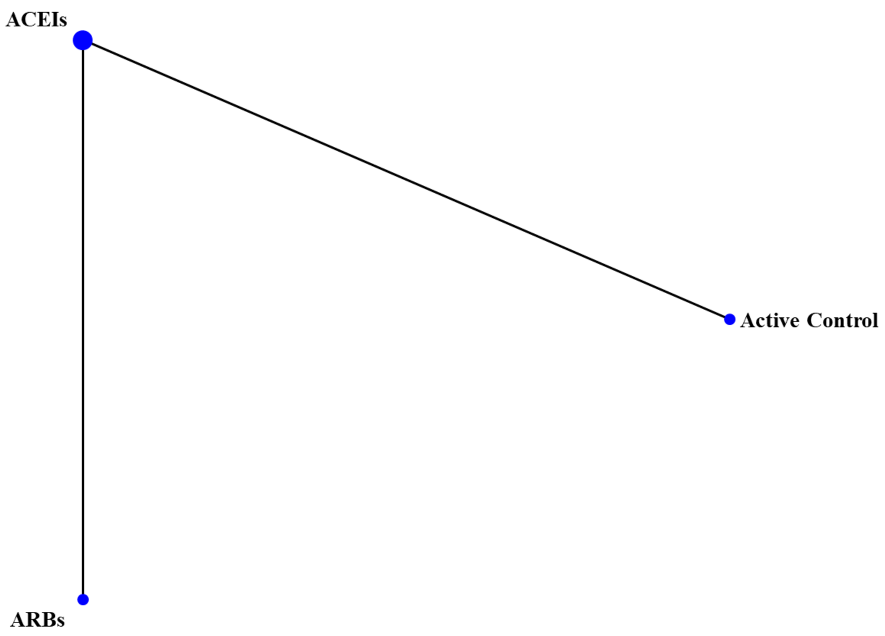

Notes: The circles (nodes) represent the available treatments and the lines (edges) represent the available comparisons. Size nodes and width of edges indicate weighting according to the numbers of studies involved for each treatment and comparison, respectively.

Abbreviations: ACEIs, angiotensin-converting enzyme inhibitors; ARBs, angiotensin II receptor blockers; MRAs, mineralocorticoid receptor antagonists.

Figure S2. Rankogram for Primary Outcomes

(A) Change in Residual Glomerular Filtration Rate (mL/min/1.73 m^2)

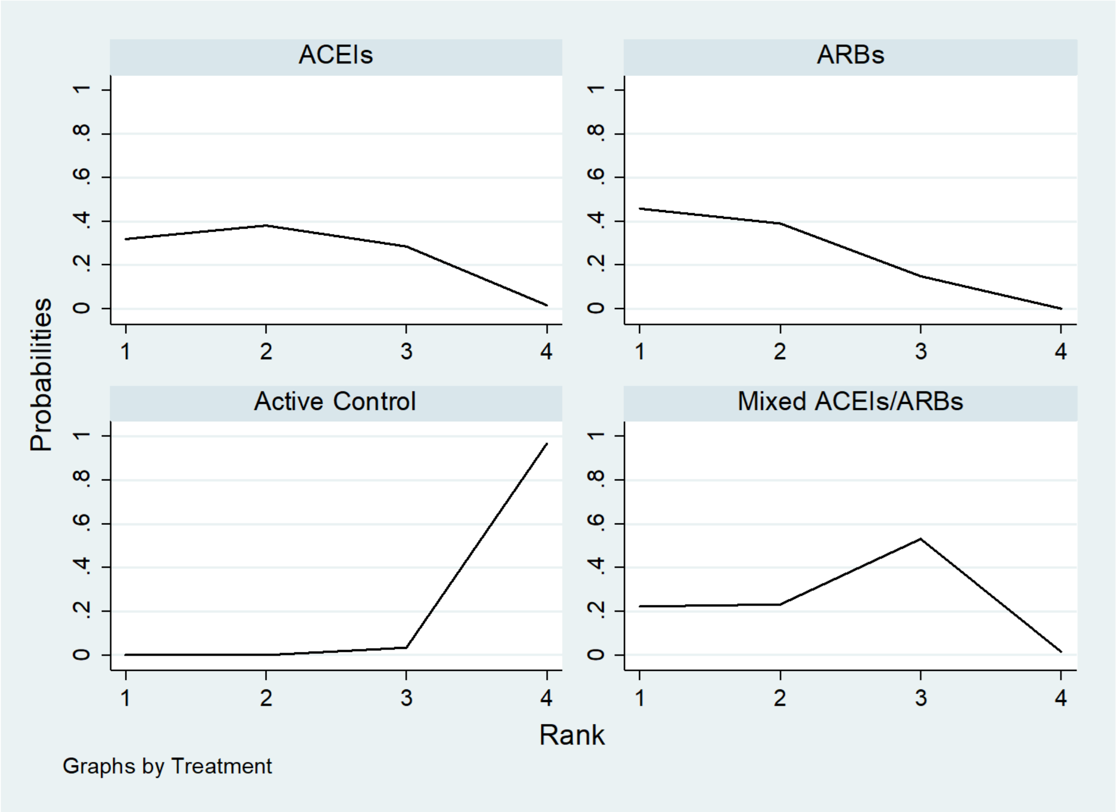

(B) Change in Urine Volume (mL/day)

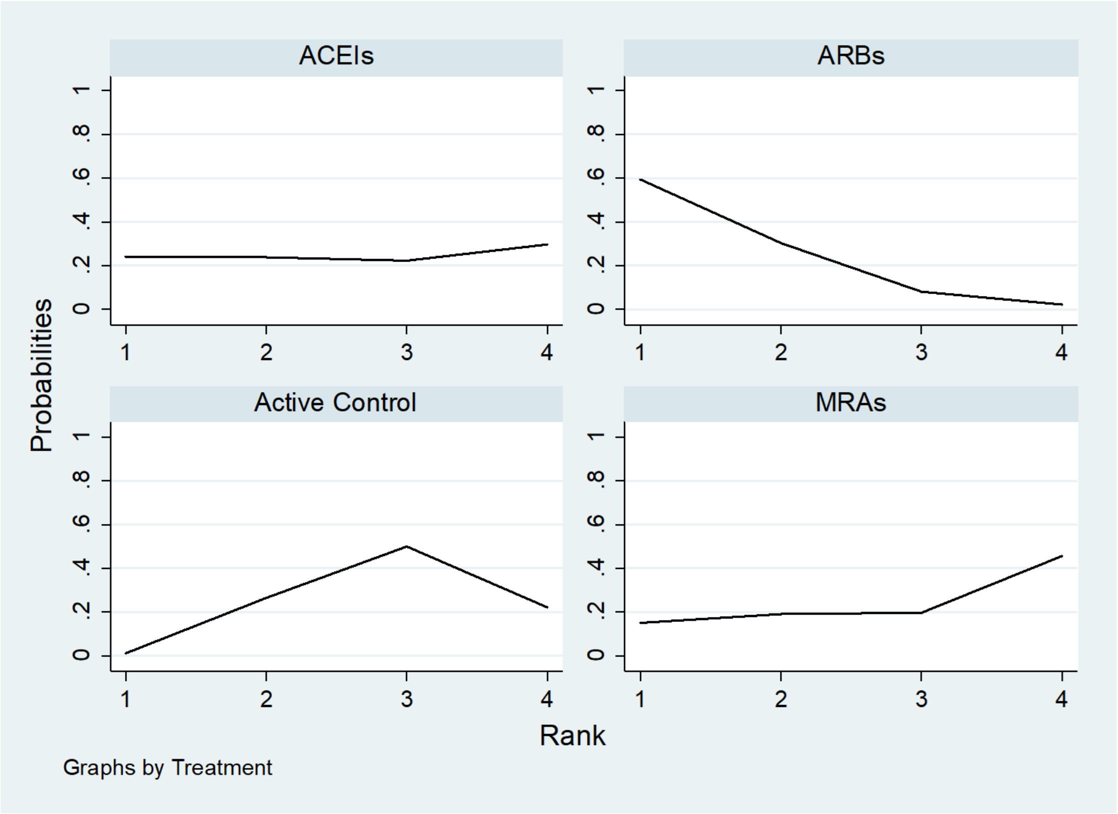

Abbreviations: ACEIs, angiotensin-converting enzyme inhibitors; ARBs, angiotensin II receptor blockers; MRAs; mineralocorticoid receptor antagonists.

Figure S2. Rankogram for Primary Outcomes (Continued)
(C) Incidence of Anuria

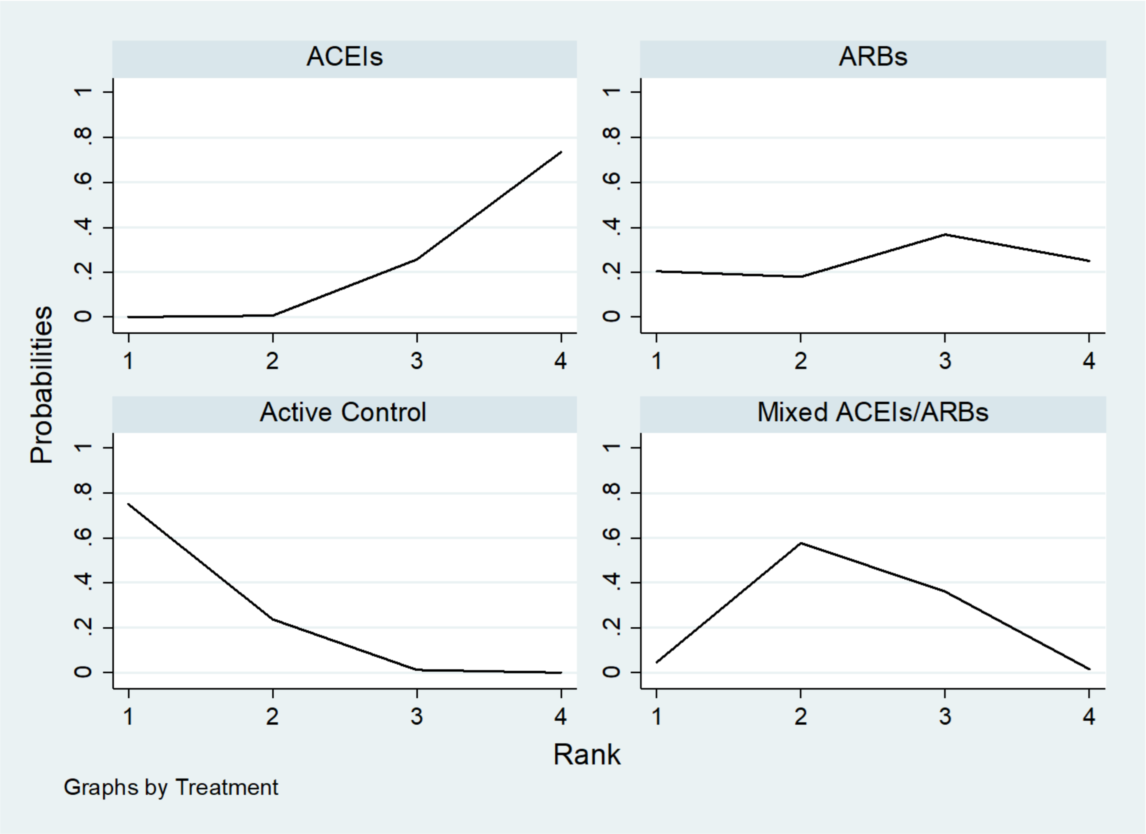

(D) Change in Dialysate-to-Plasma Creatinine Ratio

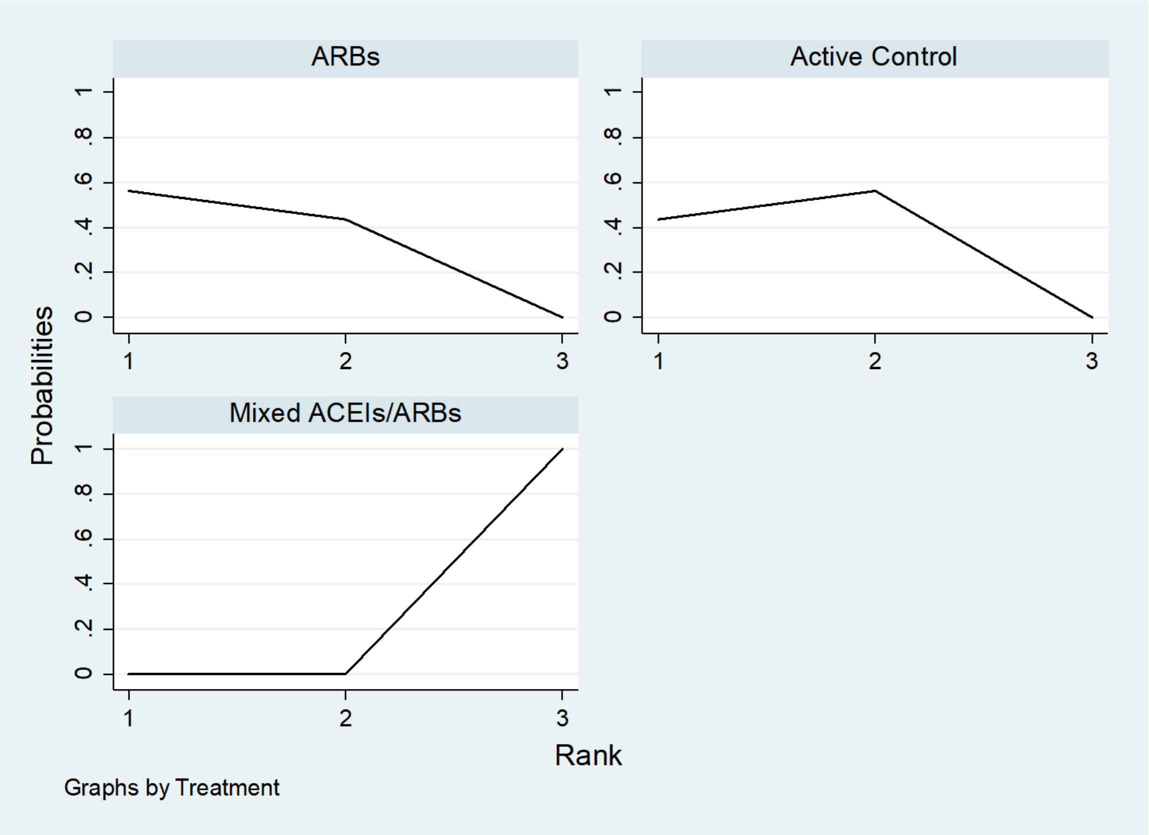

Abbreviations: ACEIs, angiotensin-converting enzyme inhibitors; ARBs, angiotensin II receptor blockers; MRAs; mineralocorticoid receptor antagonists.

Figure S2. Rankogram for Primary Outcomes (Continued)

(E) Acceptability of Treatment

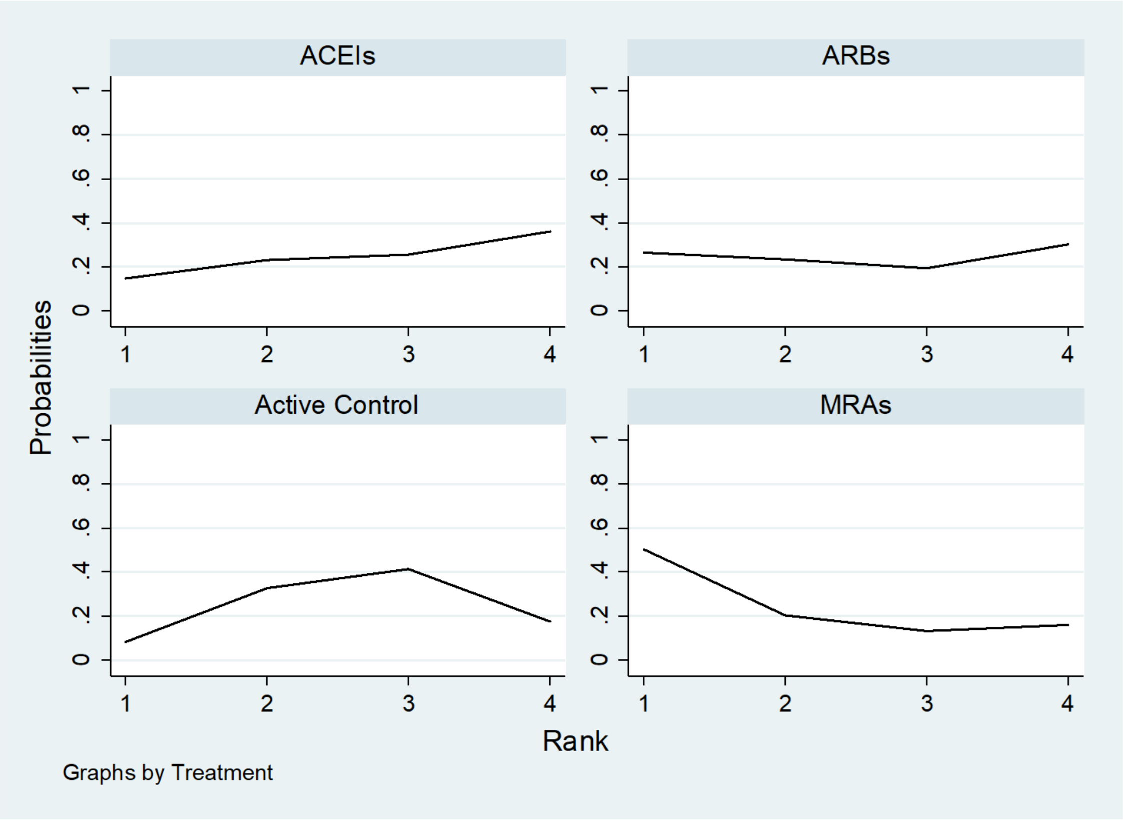

Abbreviations: ACEIs, angiotensin-converting enzyme inhibitors; ARBs, angiotensin II receptor blockers; MRAs; mineralocorticoid receptor antagonists.

Figure S3. SUCRA and Cumulative Probabilities Plots for Primary Outcomes

(A) Change in Residual Glomerular Filtration Rate (mL/min/1.73 m^2)

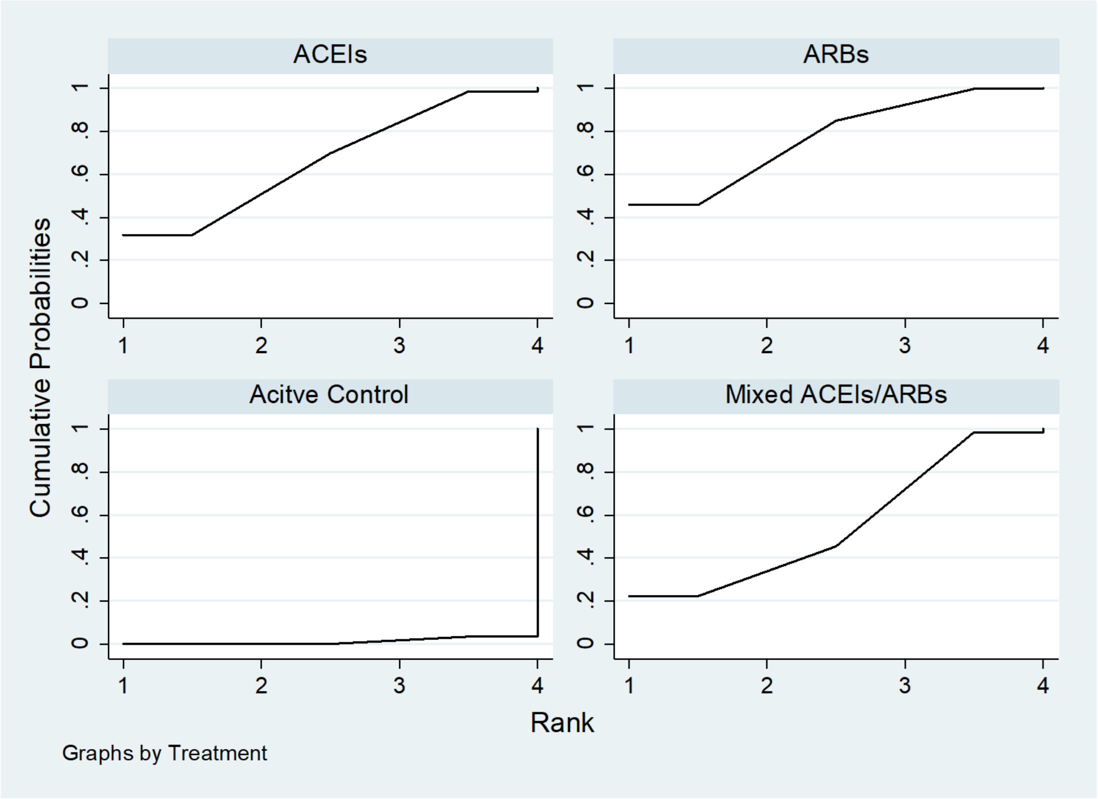

(B) Change in Urine Volume (mL/day)

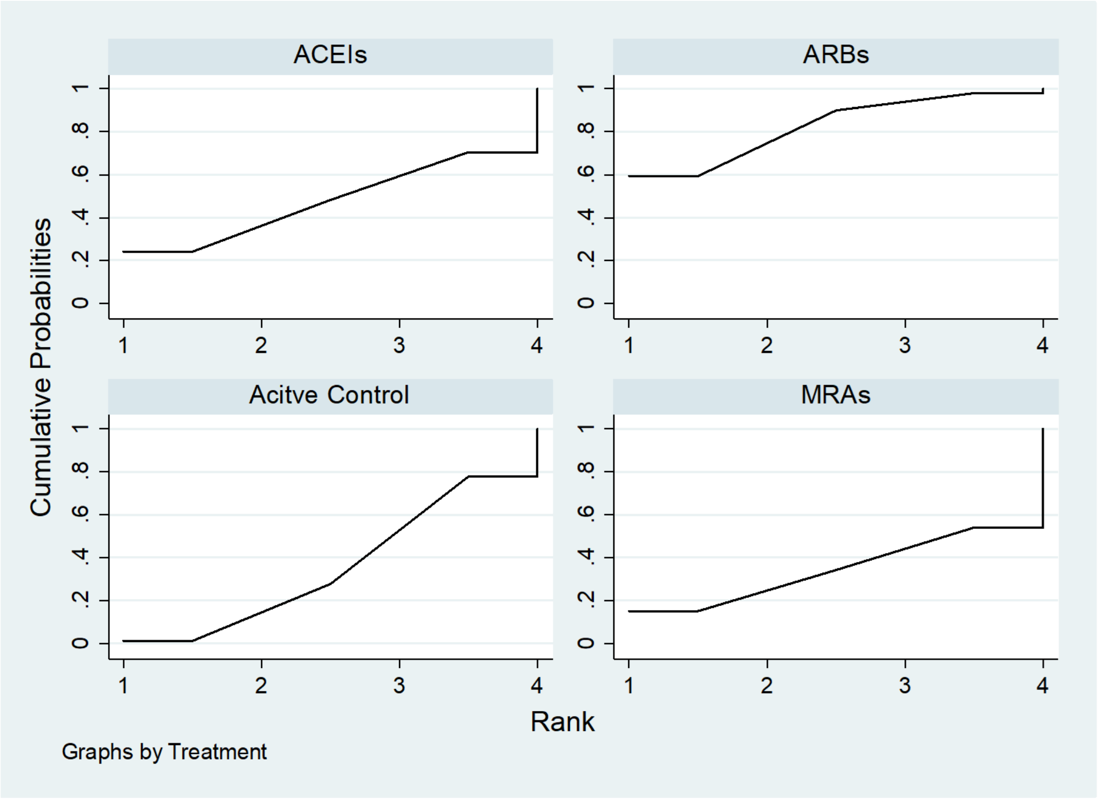

Note: In line with Cohen's guideline for magnitude of effect, SMDs of 0.2, 0.5 and 0.8 were taken to be small, medium, and large, respectively.

Abbreviations: ACEIs, angiotensin-converting enzyme inhibitors; ARBs, angiotensin II receptor blockers; MRAs; mineralocorticoid receptor antagonists; SMDs, standardised mean difference.

Figure S3. SUCRA and Cumulative Probabilities Plots for Primary Outcomes (Continued)
(C) Incidence of Anuria

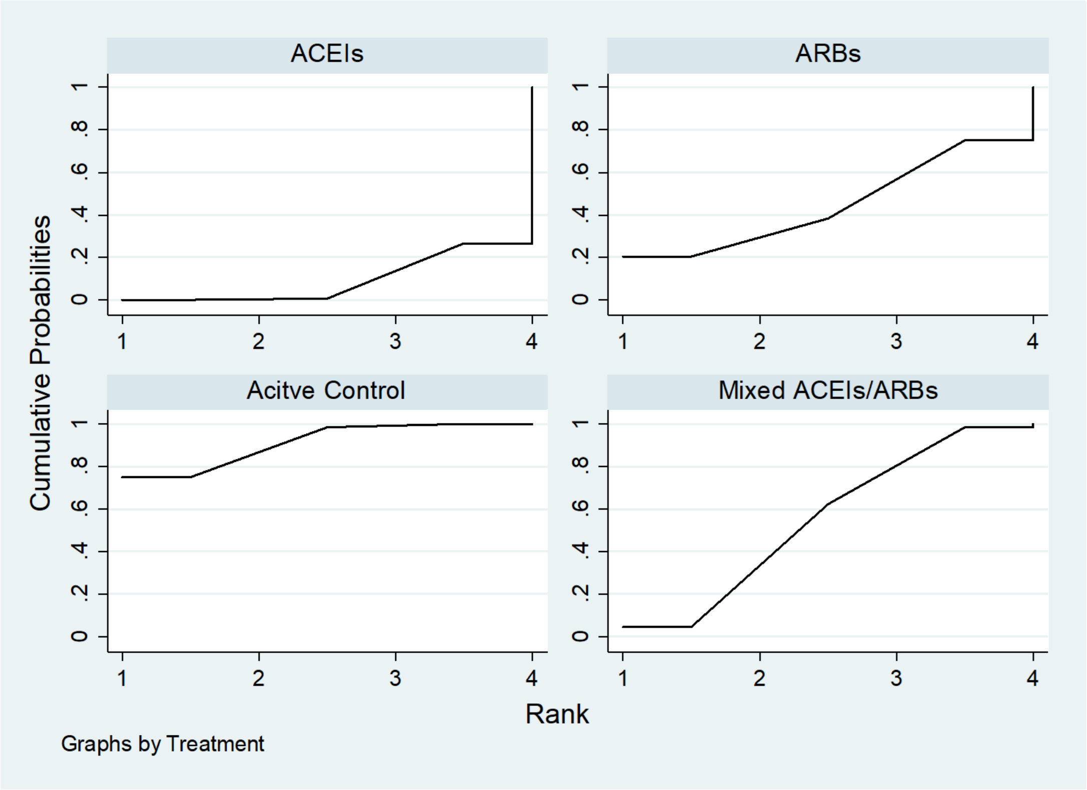

(D) Change in Dialysate-to-Plasma Creatinine Ratio

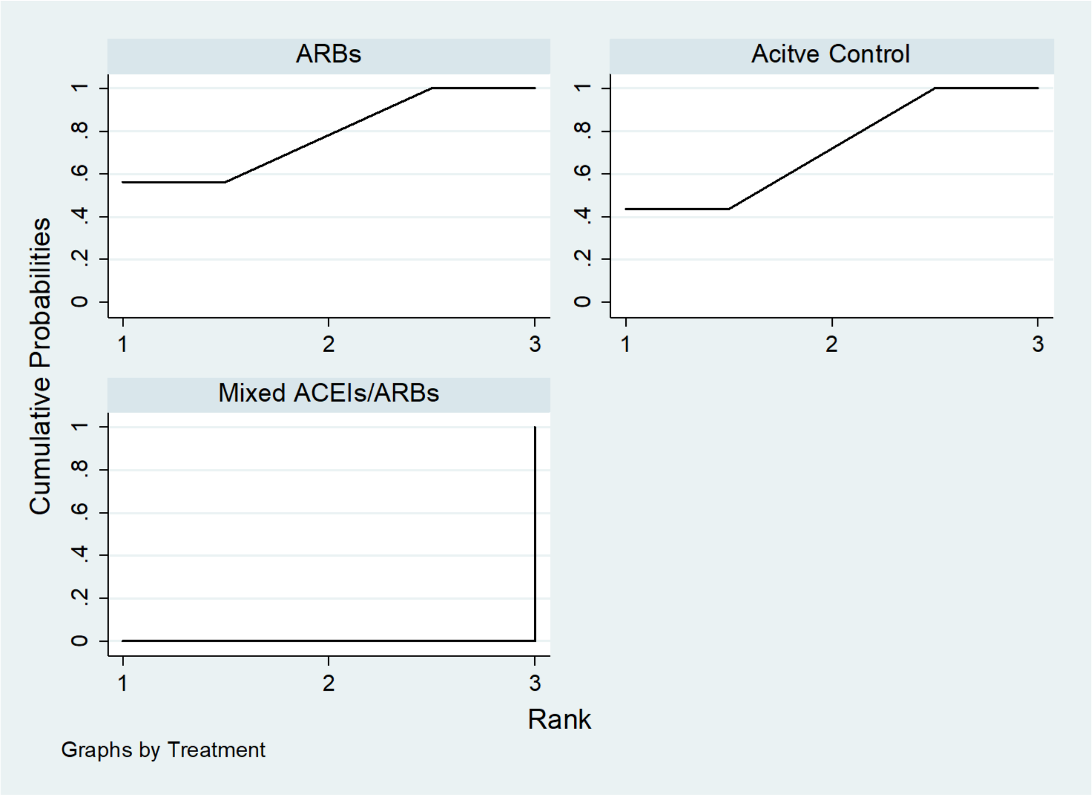

Note: In line with Cohen's guideline for magnitude of effect, SMDs of 0.2, 0.5 and 0.8 were taken to be small, medium, and large, respectively.
Abbreviations: ACEIs, angiotensin-converting enzyme inhibitors; ARBs, angiotensin II receptor blockers; MRAs; mineralocorticoid receptor antagonists; SMDs, standardised mean difference.

Figure S3. SUCRA and Cumulative Probabilities Plots for Primary Outcomes (Continued)
(E) Acceptability of Treatment

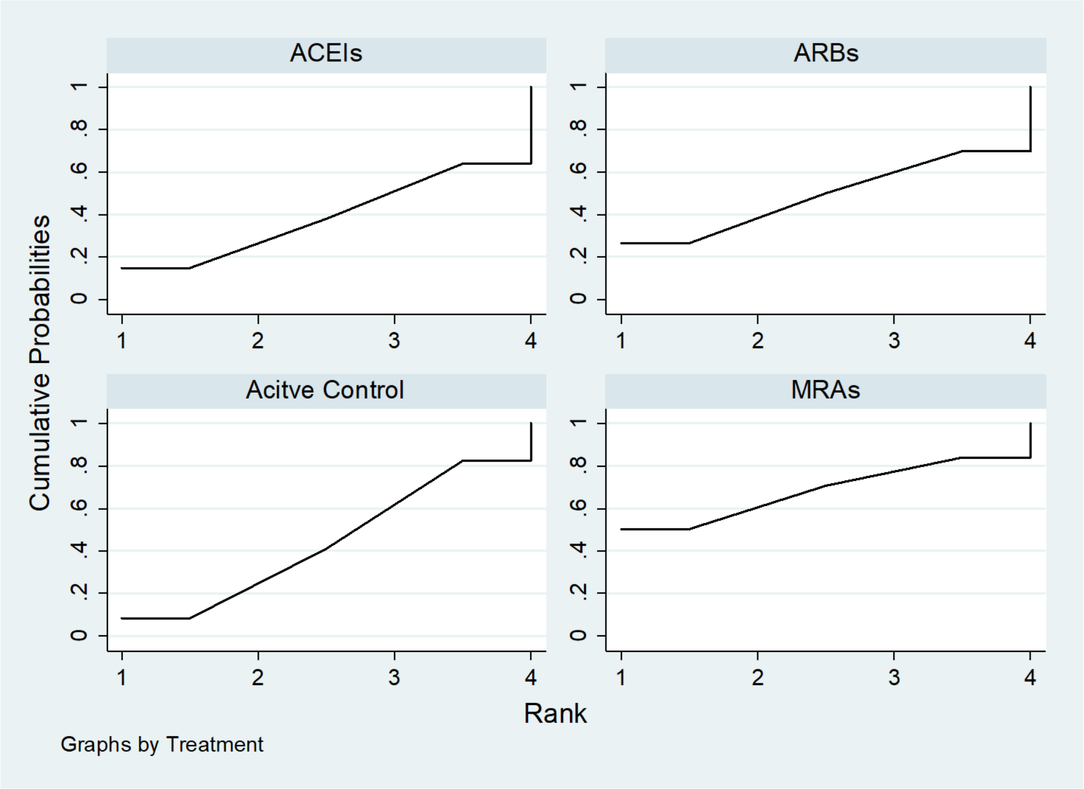

Note: In line with Cohen's guideline for magnitude of effect, SMDs of 0.2, 0.5 and 0.8 were taken to be small, medium, and large, respectively.
Abbreviations: ACEIs, angiotensin-converting enzyme inhibitors; ARBs, angiotensin II receptor blockers; MRAs; mineralocorticoid receptor antagonists; SMDs, standardised mean difference.

Figure S4. Comparison-Adjusted Funnel Plot for Primary Outcomes

(A) Residual Glomerular Filtration Rate (mL/min/1.73 m^2)

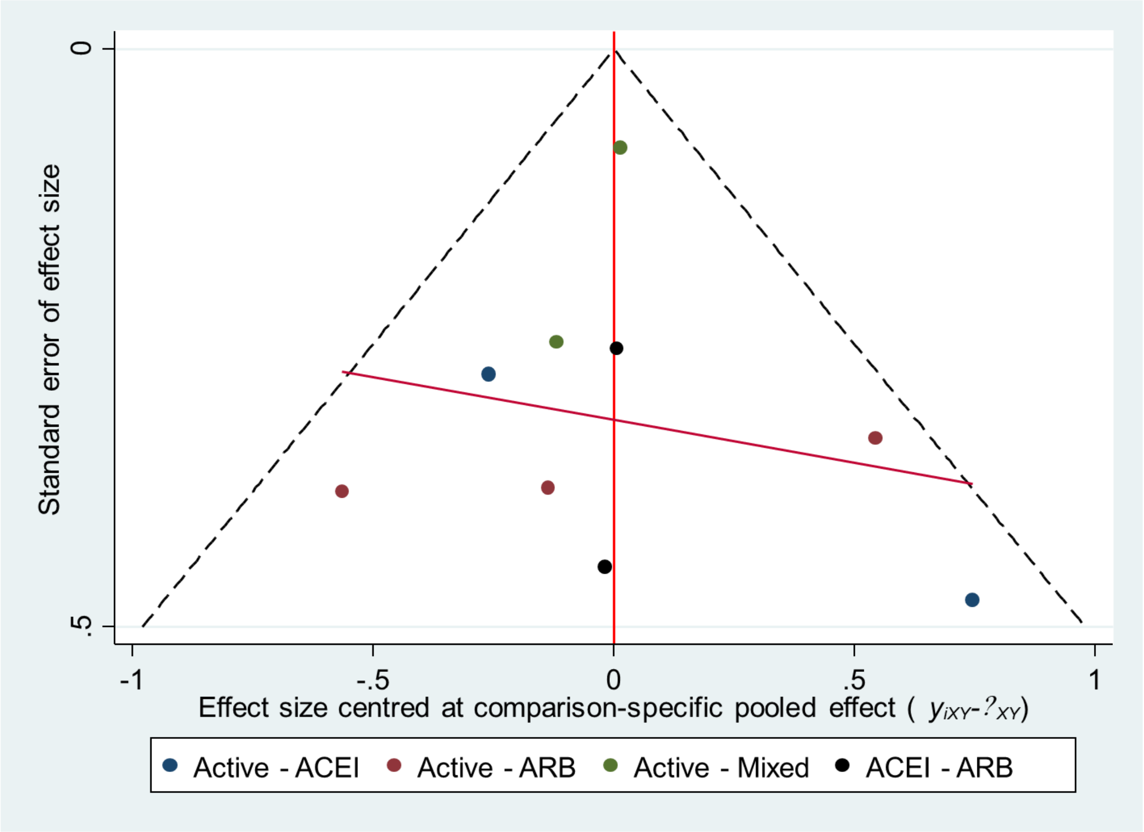

(B) Urine Volume (mL/day)

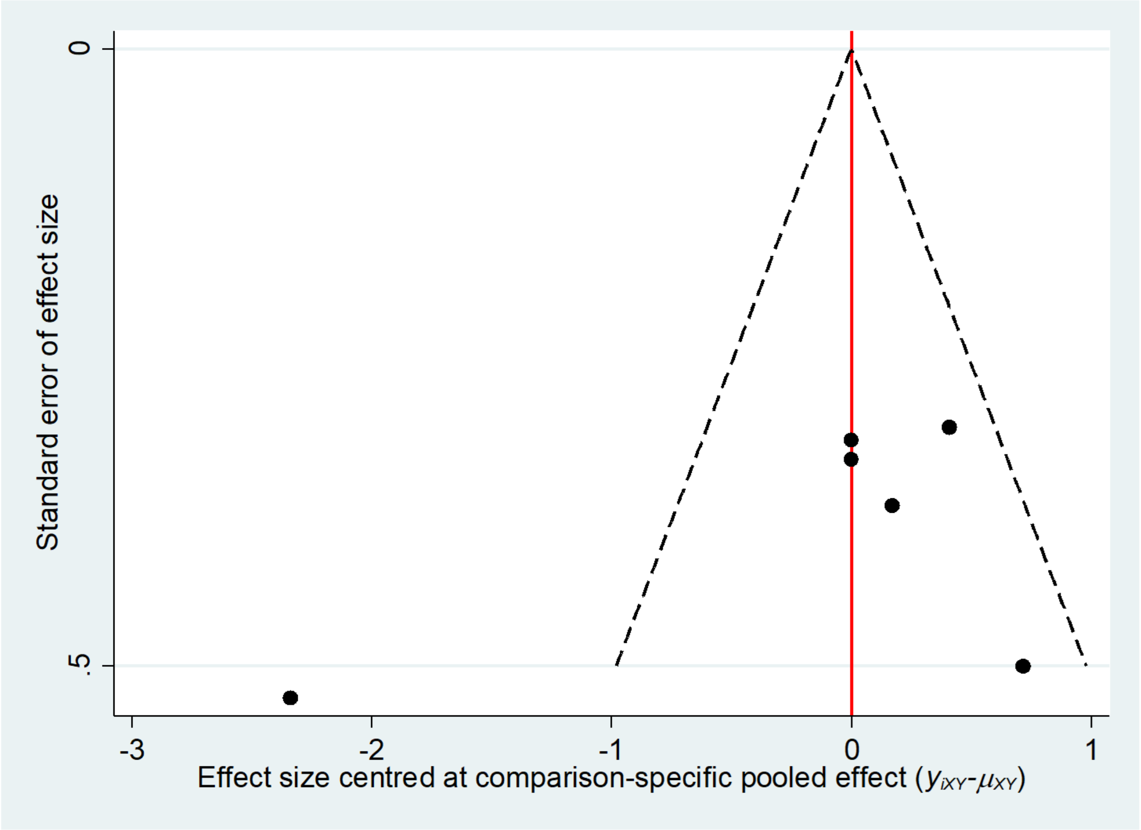

Note: all treatment vs. active control.

Figure S4. Comparison-Adjusted Funnel Plot for Primary Outcomes (Continued)
(C) Incidence of Anuria

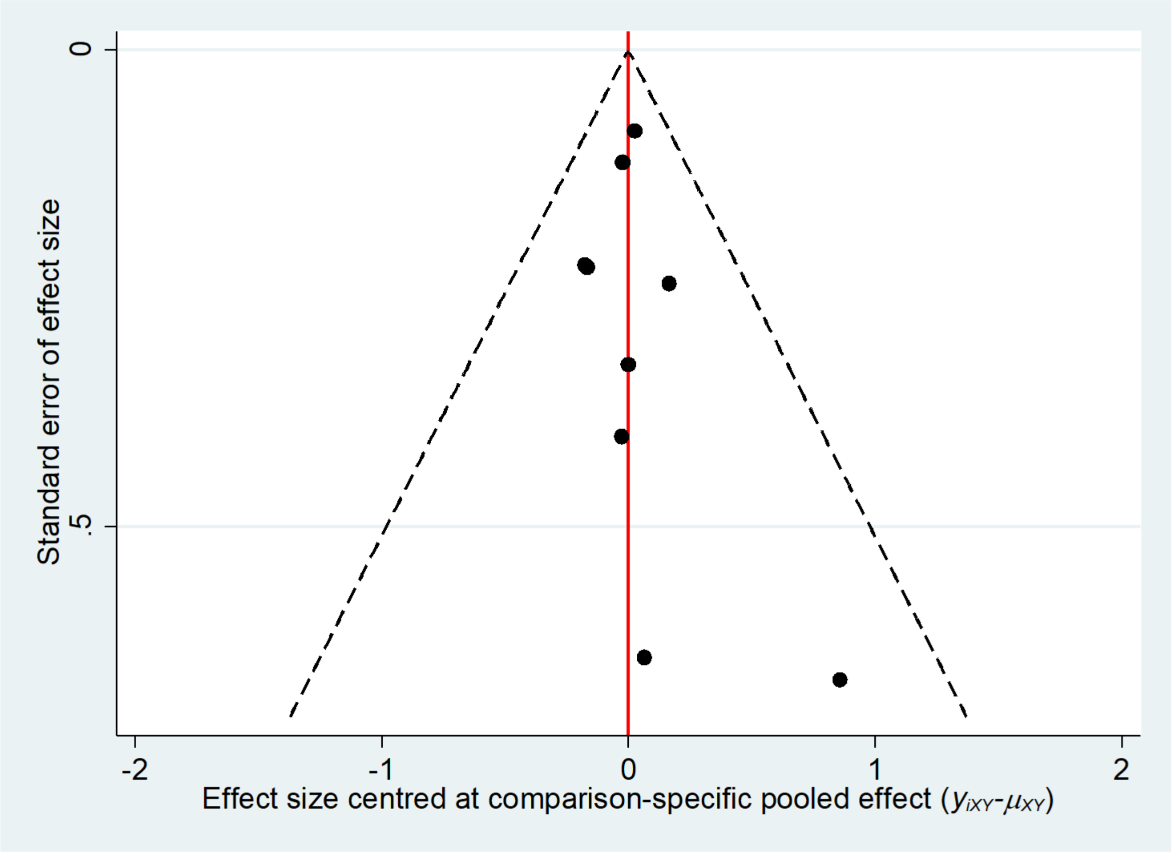

(D) Dialysate-to-Plasma Creatinine Ratio

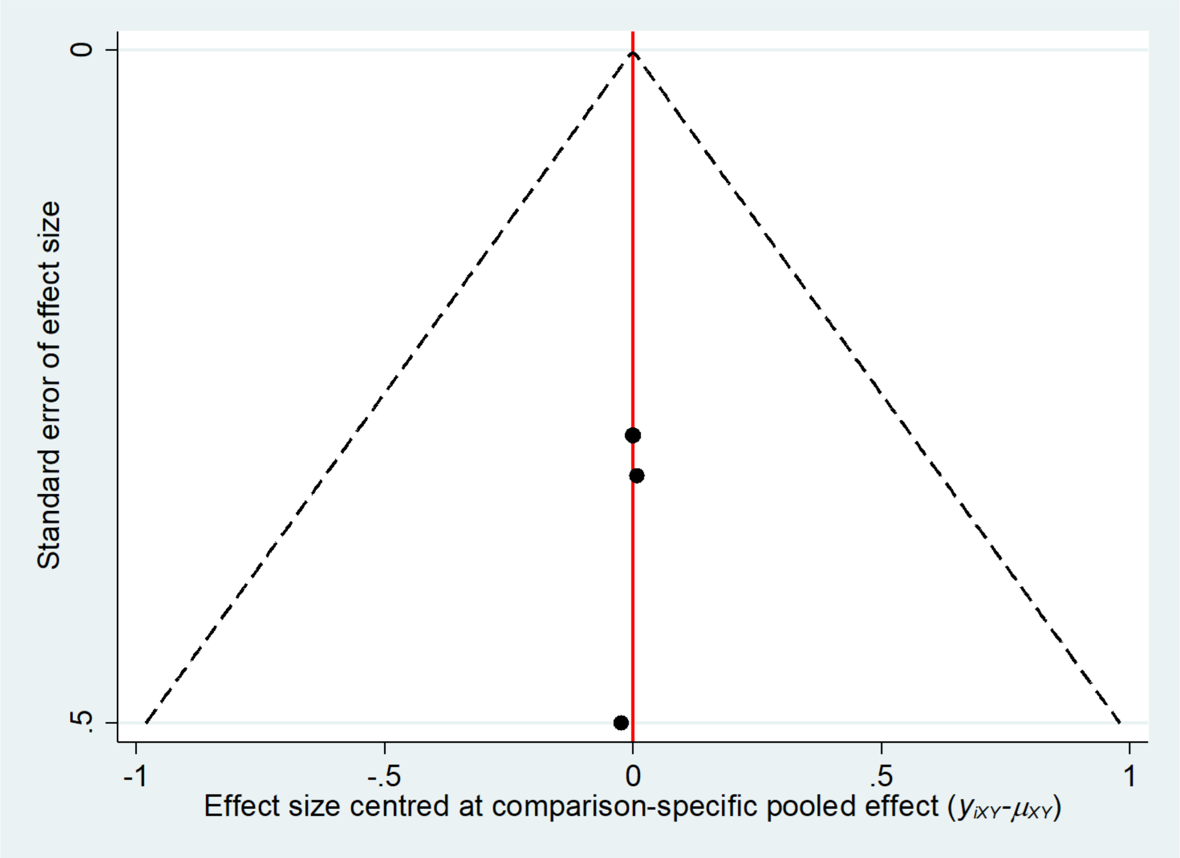

Note: all treatment vs. active control.

Figure S4. Comparison-Adjusted Funnel Plot for Primary Outcomes (Continued)
(E) Acceptability of Treatment

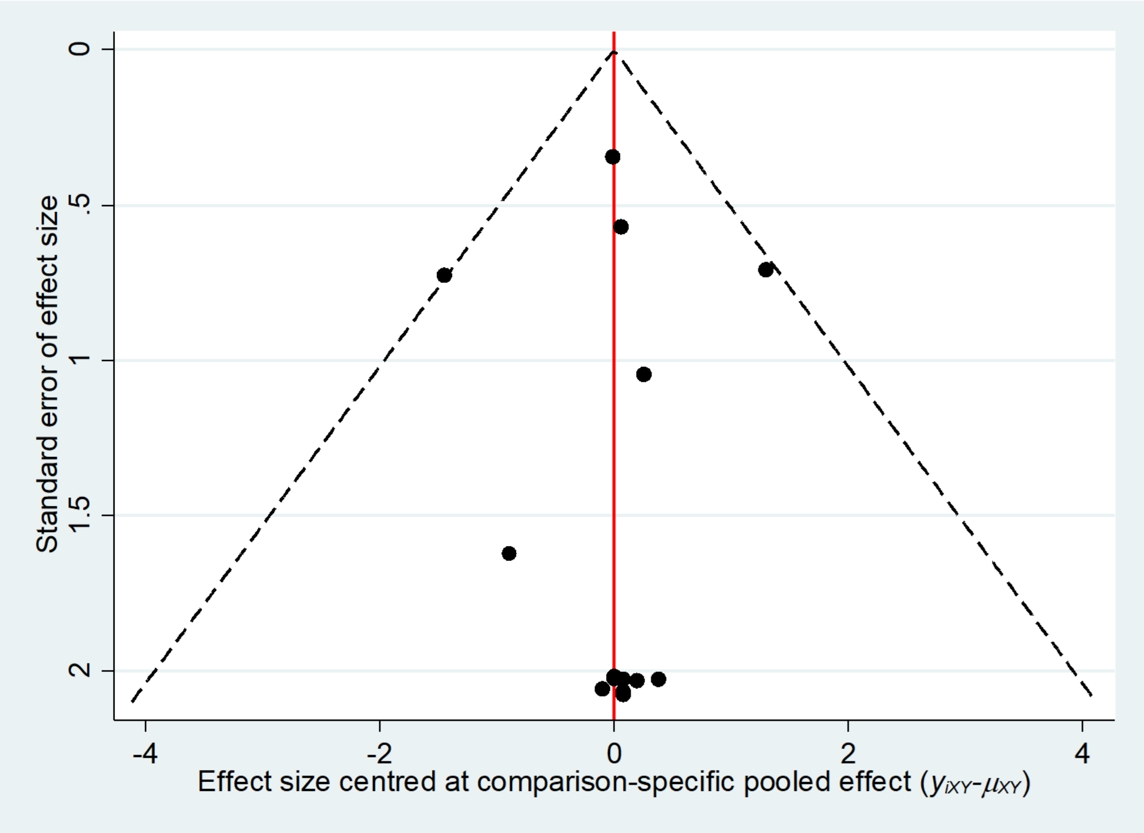

Note: all treatment vs. active control.

Figure S5. Two-Dimension Rank Plot of Effect Estimates

(A) Efficacy on Preservation of rGFR and Acceptability of Treatment

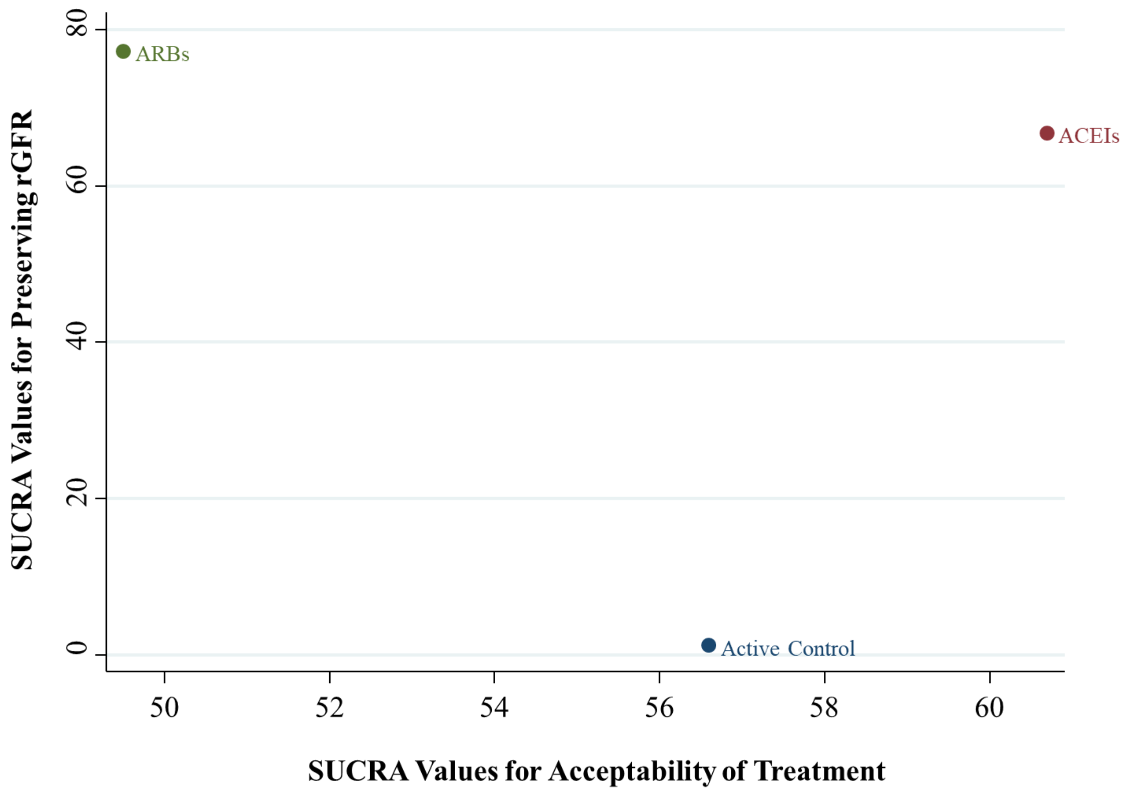

(B) Efficacy on Prevention of Anuria and Acceptability of Treatment

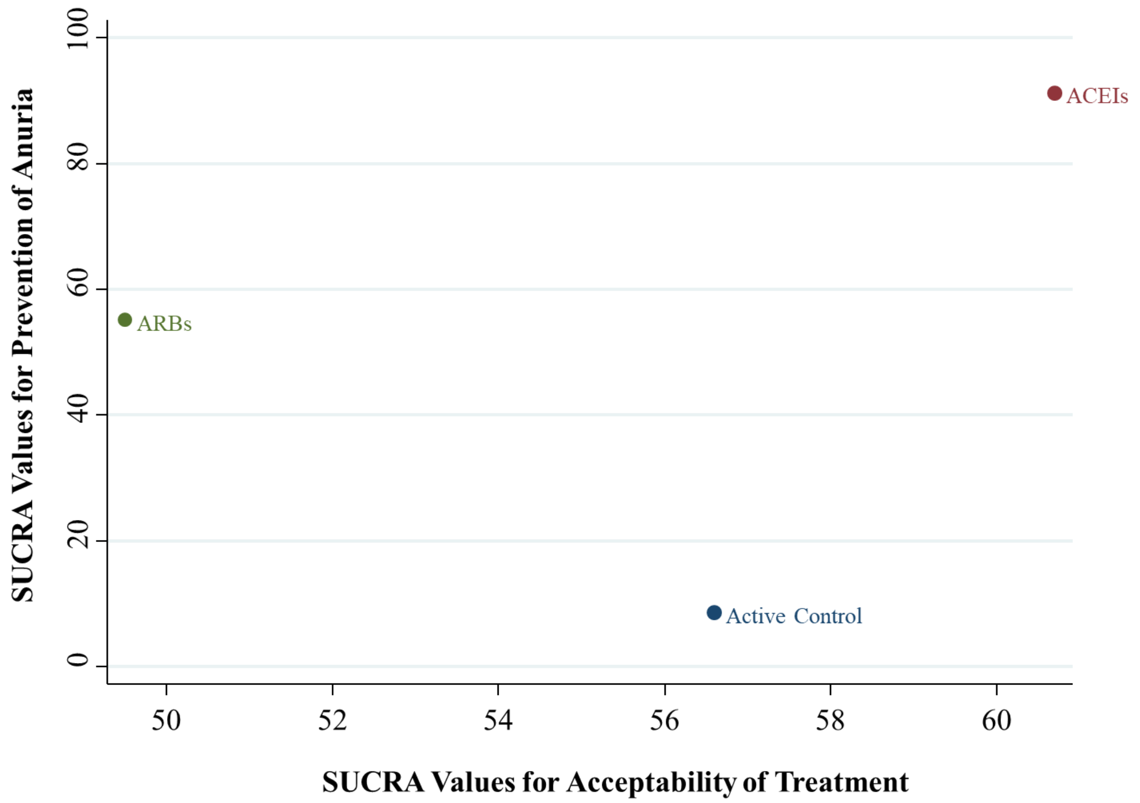

Abbreviations: ACEIs, angiotensin-converting enzyme inhibitors; ARBs, angiotensin II receptor blockers; rGFR, residual glomerular filtration rate; SUCRA, surface under the cumulative ranking curve.

Appendix-I. PRISMA NMA Checklist of Items to Include When Reporting Systematic Review Involving a Network Meta-analysis

Section/Topic	Item #	Checklist Item	
**TITLE**			
Title	1	Identify the report as a systematic review *incorporating a network meta-analysis (or related form of meta-analysis).*	✓
**ABSTRACT**			
Structured summary	2	Provide a structured summary including, as applicable:<br>**Background:** main objectives<br>**Methods:** data sources; study eligibility criteria, participants, and interventions; study appraisal; and *synthesis methods, such as network meta-analysis.*<br>**Results:** number of studies and participants identified; summary estimates with corresponding confidence/credible intervals; *treatment rankings may also be discussed. Authors may choose to summarize pairwise comparisons against a chosen treatment included in their analyses for brevity.*<br>**Discussion/Conclusions:** limitations; conclusions and implications of findings.<br>**Other:** primary source of funding; systematic review registration number with registry name.	✓
**INTRODUCTION**			
Rationale	3	Describe the rationale for the review in the context of what is already known, *including mention of why a network meta-analysis has been conducted.*	✓
Objectives	4	Provide an explicit statement of questions being addressed, with reference to participants, interventions, comparisons, outcomes, and study design (PICOS).	✓
**METHODS**			
Protocol and registration	5	Indicate whether a review protocol exists and if and where it can be accessed (e.g., Web address); and, if available, provide registration information, including registration number.	✓
Eligibility criteria	6	Specify study characteristics (e.g., PICOS, length of follow-up) and report characteristics (e.g., years considered, language, publication status) used as criteria for eligibility, giving rationale. *Clearly describe eligible treatments included in the treatment network, and note whether any have been clustered or merged into the same node (with justification).*	✓
Information sources	7	Describe all information sources (e.g., databases with dates of coverage, contact with study authors to identify additional studies) in the search and date last searched.	✓
Search	8	Present full electronic search strategy for at least one database, including any limits used, such that it could be repeated.	✓
Study selection	9	State the process for selecting studies (i.e., screening, eligibility, included in systematic review, and, if applicable, included in the meta-analysis).	✓

PICOS = population, intervention, comparators, outcomes, study design.
*Text in italics indicates wording specific to reporting of network meta-analyses that has been added to guidance from the PRISMA statement.
†Authors may wish to plan for use of appendices to present all relevant information in full detail for items in this section.

Appendix-I. PRISMA NMA Checklist of Items to Include When Reporting Systematic Review Involving a Network Meta-analysis (Continued)

Section/Topic	Item #	Checklist Item	
**METHODS**			
Data collection process	10	Describe method of data extraction from reports (e.g., piloted forms, independently, in duplicate) and any processes for obtaining and confirming data from investigators.	✓
Data items	11	List and define all variables for which data were sought (e.g., PICOS, funding sources) and any assumptions and simplifications made.	✓
**Geometry of the network**	**S1**	Describe methods used to explore the geometry of the treatment network under study and potential biases related to it. This should include how the evidence base has been graphically summarized for presentation, and what characteristics were compiled and used to describe the evidence base to readers.	✓
Risk of bias within individual studies	12	Describe methods used for assessing risk of bias of individual studies (including specification of whether this was done at the study or outcome level), and how this information is to be used in any data synthesis.	✓
Summary measures	13	State the principal summary measures (e.g., risk ratio, difference in means). *Also describe the use of additional summary measures assessed, such as treatment rankings and surface under the cumulative ranking curve (SUCRA) values, as well as modified approaches used to present summary findings from meta-analyses.*	✓
Planned methods of analysis	14	Describe the methods of handling data and combining results of studies for each network meta-analysis. This should include, but not be limited to:<br>• *Handling of multi-arm trials;*<br>• *Selection of variance structure;*<br>• *Selection of prior distributions in Bayesian analyses; and*<br>• *Assessment of model fit.*	✓
**Assessment of Inconsistency**	**S2**	Describe the statistical methods used to evaluate the agreement of direct and indirect evidence in the treatment network(s) studied. Describe efforts taken to address its presence when found.	✓
Risk of bias across studies	15	Specify any assessment of risk of bias that may affect the cumulative evidence (e.g., publication bias, selective reporting within studies).	✓
Additional analyses	16	Describe methods of additional analyses if done, indicating which were pre-specified. This may include, but not be limited to, the following:<br>• Sensitivity or subgroup analyses;<br>• Meta-regression analyses;<br>• *Alternative formulations of the treatment network; and*<br>• *Use of alternative prior distributions for Bayesian analyses (if applicable).*	✓

PICOS = population, intervention, comparators, outcomes, study design.
*Text in italics indicates wording specific to reporting of network meta-analyses that has been added to guidance from the PRISMA statement.
†Authors may wish to plan for use of appendices to present all relevant information in full detail for items in this section.

Appendix-I. PRISMA NMA Checklist of Items to Include When Reporting Systematic Review Involving a Network Meta-analysis (Continued)

Section/Topic	Item #	Checklist Item	
**RESULTS**†			
Study selection	17	Give numbers of studies screened, assessed for eligibility, and included in the review, with reasons for exclusions at each stage, ideally with a flow diagram.	✓
**Presentation of network structure**	**S3**	Provide a network graph of the included studies to enable visualization of the geometry of the treatment network.	✓
**Summary of network geometry**	**S4**	Provide a brief overview of characteristics of the treatment network. This may include commentary on the abundance of trials and randomised patients for the different interventions and pairwise comparisons in the network, gaps of evidence in the treatment network, and potential biases reflected by the network structure.	✓
Study characteristics	18	For each study, present characteristics for which data were extracted (e.g., study size, PICOS, follow-up period) and provide the citations.	✓
Risk of bias within studies	19	Present data on risk of bias of each study and, if available, any outcome level assessment.	✓
Results of individual studies	20	For all outcomes considered (benefits or harms), present, for each study: 1) simple summary data for each intervention group, and 2) effect estimates and confidence intervals. *Modified approaches may be needed to deal with information from larger networks.*	✓
Synthesis of results	21	Present results of each meta-analysis done, including confidence/credible intervals. *In larger networks, authors may focus on comparisons versus a particular comparator (e.g. placebo or standard care), with full findings presented in an appendix. League tables and forest plots may be considered to summarize pairwise comparisons.* If additional summary measures were explored (such as treatment rankings), these should also be presented.	✓
**Exploration for inconsistency**	**S5**	Describe results from investigations of inconsistency. This may include such information as measures of model fit to compare consistency and inconsistency models, *P* values from statistical tests, or summary of inconsistency estimates from different parts of the treatment network.	✓
Risk of bias across studies	22	Present results of any assessment of risk of bias across studies for the evidence base being studied.	✓
Results of additional analyses	23	Give results of additional analyses, if done (e.g., sensitivity or subgroup analyses, meta-regression analyses, *alternative network geometries studied, alternative choice of prior distributions for Bayesian analyses,* and so forth).	✓

PICOS = population, intervention, comparators, outcomes, study design.
*Text in italics indicates wording specific to reporting of network meta-analyses that has been added to guidance from the PRISMA statement.
†Authors may wish to plan for use of appendices to present all relevant information in full detail for items in this section.

Appendix-I. PRISMA NMA Checklist of Items to Include When Reporting Systematic Review Involving a Network Meta-analysis (Continued)

Section/Topic	Item #	Checklist Item	
**DISCUSSION**			
Summary of evidence	24	Summarize the main findings, including the strength of evidence for each main outcome; consider their relevance to key groups (e.g., healthcare providers, users, and policy-makers).	✓
Limitations	25	Discuss limitations at study and outcome level (e.g., risk of bias), and at review level (e.g., incomplete retrieval of identified research, reporting bias). *Comment on the validity of the assumptions, such as transitivity and consistency. Comment on any concerns regarding network geometry (e.g., avoidance of certain comparisons).*	✓
Conclusions	26	Provide a general interpretation of the results in the context of other evidence, and implications for future research.	✓
**FUNDING**			
Funding	27	Describe sources of funding for the systematic review and other support (e.g., supply of data); role of funders for the systematic review. This should also include information regarding whether funding has been received from manufacturers of treatments in the network and/or whether some of the authors are content experts with professional conflicts of interest that could affect use of treatments in the network.	✓

PICOS = population, intervention, comparators, outcomes, study design.

*Text in italics indicates wording specific to reporting of network meta-analyses that has been added to guidance from the PRISMA statement.

†Authors may wish to plan for use of appendices to present all relevant information in full detail for items in this section.

Appendix-II. Systematic Review Search Strategy: PubMed, From Inception to 31 May, 2019

Search Number	Input Terms	Items Found
#1	((((((Angiotensin Converting Enzyme Inhibitors) OR Angiotensin I Converting Enzyme Inhibitors) OR Angiotensin Converting Enzyme Antagonists) OR ACE Inhibitors) OR Kininase II Antagonists) OR Kininase II Inhibitors)	54,939
#2	(((((Angiotensin Receptor Blockers) OR Angiotensin Receptor Antagonists) OR Angiotensin II Receptor Antagonists) OR Angiotensin II Receptor Blockers) OR ARBs)	33,446
#3	((((Aldosterone Receptor Antagonists) OR Aldosterone Antagonists) OR Mineralocorticoid Receptor Antagonists) OR Mineralocorticoid Antagonists)	13,731
#4	((Renin Inhibitor) OR Direct Renin Inhibitors)	8,243
#5	#1 OR #2 OR #3 OR #4	86,221
#6	(((((((((Benazepril) OR Captopril) OR Enalapril) OR Fosinopril) OR Lisinopril) OR Moexipril) OR Perindopril) OR Quinapril) OR Ramipril) OR Trandolapril)	286,700
#7	((((((((Eprosartan) OR Olmesartan) OR Valsartan) OR Losartan) OR Telmisartan) OR Candesartan) OR Azilsartan) OR Irbesartan)	19,853
#8	((Spironolactone) OR Eplerenone)	9,013
#9	Aliskiren	1,206
#10	#6 OR #7 OR #8 OR #9	54,520
#11	#5 OR #10	94,716
#12	((((((((Renal Replacement Therapy) OR Dialysis) OR Renal Dialysis) OR Peritoneal Dialysis) OR Continuous Ambulatory Peritoneal Dialysis) OR Automated Peritoneal Dialysis) OR CAPD) OR APD)	270,323
#13	((((((((((((((Renal function) OR Residual renal function) OR Glomerular filtration rate) OR Urine volume) OR Proteinuria) OR Membrane function) OR Ultrafiltration rate) OR Protein loss) OR Peritoneal equilibration test) OR Peritoneal membrane function) OR Peritoneal protein loss) OR Peritoneal dialysis adequacy) OR Adverse drug event) OR Adverse drug reaction) OR Drug Side Effect)	2,050,366
#14	#12 AND #13	114,694
#15	#11 AND #14	1,997
#16	((((((((Case reports[Publication Type]) OR Comment[Publication Type]) OR Editorial[Publication Type]) OR Guideline[Publication Type]) OR Letter[Publication Type]) OR Meta-Analysis[Publication Type]) OR News[Publication Type]) OR Newspaper article[Publication Type]) OR Practice guideline[Publication Type]	3,871,760
#17	#15 NOT #16	1,724
#18	#17 Filters: Humans	1,541

Appendix-II. Systematic Review Search Strategy: Embase (via OVID), From Inception to 31 May, 2019 (Continued)

Search Number	Input Terms	Items Found
#1	(Angiotensin Converting Enzyme Inhibitors or Angiotensin I Converting Enzyme Inhibitors or Angiotensin Converting Enzyme Antagonists or ACE Inhibitors or Kininase II Antagonists or Kininase II Inhibitors).mp.	32,535
#2	(Angiotensin Receptor Blockers or Angiotensin Receptor Antagonists or Angiotensin II Receptor Antagonists or Angiotensin II Receptor Blockers or ARBs).mp.	13,866
#3	(Aldosterone Receptor Antagonists or Aldosterone Antagonists or Mineralocorticoid Receptor Antagonists or Mineralocorticoid Antagonists).mp.	2,693
#4	(Renin Inhibitor or Direct Renin Inhibitors).mp.	3,973
#5	1 OR 2 OR 3 OR 4	42,731
#6	(Benazepril or Captopril or Enalapril or Fosinopril or Lisinopril or Moexipril or Perindopril or Quinapril or Ramipril or Trandolapril).mp.	83,556
#7	(Eprosartan or Olmesartan or Valsartan or Losartan or Telmisartan or Candesartan or Azilsartan or Irbesartan).mp.	50,796
#8	(Spironolactone or Eplerenone).mp.	28,613
#9	aliskiren plus amlodipine/ or aliskiren plus valsartan/ or aliskiren/ or aliskiren plus hydrochlorothiazide/ or aliskiren plus amlodipine plus hydrochlorothiazide/ or Aliskiren.mp	3,347
#10	6 OR 7 OR 8 OR 9	141,467
#11	5 OR 10	163,474
#12	(Renal Replacement Therapy or Dialysis or Renal Dialysis or Peritoneal Dialysis or Continuous Ambulatory Peritoneal Dialysis or Automated Peritoneal Dialysis or CAPD or APD).mp.	190,689
#13	(Renal function or Residual renal function or Glomerular filtration rate or Urine volume or Proteinuria or Membrane function or Ultrafiltration rate or Protein loss or Peritoneal equilibration test or Peritoneal membrane function or Peritoneal protein loss or Peritoneal dialysis adequacy or Adverse drug event or Adverse drug reaction or Drug Side Effect).mp.	1,549,039
#14	12 AND 13	39,969
#15	11 AND 14	1,860
#16	exp Animals/ not (exp Animals/ and Humans/)	12,385,120
#17	15 NOT 16	1,043

Appendix-II. Systematic Review Search Strategy: Scopus, From Inception to 31 May, 2019 (Continued)

Search Number	Input Terms	Items Found
#1	(TITLE-ABS-KEY (Angiotensin Converting Enzyme Inhibitors) OR TITLE-ABS-KEY (Angiotensin I Converting Enzyme Inhibitors) OR TITLE-ABS-KEY (Angiotensin Converting Enzyme Antagonists) OR TITLE-ABS-KEY (ACE Inhibitors) OR TITLE-ABS-KEY (Kininase II Antagonists) OR TITLE-ABS-KEY (Kininase II Inhibitors))	58,361
#2	(TITLE-ABS-KEY (Angiotensin Receptor Blockers) OR TITLE-ABS-KEY (Angiotensin Receptor Antagonists) OR TITLE-ABS-KEY (Angiotensin II Receptor Antagonists) OR TITLE-ABS-KEY (Angiotensin II Receptor Blockers) OR TITLE-ABS-KEY (ARBs))	74,259
#3	(TITLE-ABS-KEY (Aldosterone Receptor Antagonists) OR TITLE-ABS-KEY (Aldosterone Antagonists) OR TITLE-ABS-KEY (Mineralocorticoid Receptor Antagonists) OR TITLE-ABS-KEY (Mineralocorticoid Antagonists))	25,273
#4	(TITLE-ABS-KEY (Renin Inhibitor) OR TITLE-ABS-KEY (Direct Renin Inhibitors))	26,481
#5	#1 OR #2 OR #3 OR #4	124,580
#6	(TITLE-ABS-KEY (Benazepril) OR TITLE-ABS-KEY (Captopril) OR TITLE-ABS-KEY (Enalapril) OR TITLE-ABS-KEY (Fosinopril) OR TITLE-ABS-KEY(Lisinopril) OR TITLE-ABS-KEY(Moexipril) OR TITLE-ABS-KEY (Perindopril) OR TITLE-ABS-KEY (Quinapril) OR TITLE-ABS-KEY (Ramipril) OR TITLE-ABS-KEY (Trandolapril))	79,009
#7	(TITLE-ABS-KEY (Eprosartan) OR TITLE-ABS-KEY(Olmesartan) OR TITLE-ABS-KEY (Valsartan) OR TITLE-ABS-KEY (Losartan) OR TITLE-ABS-KEY (Telmisartan) OR TITLE-ABS-KEY (Candesartan) OR TITLE-ABS-KEY (Azilsartan) OR TITLE-ABS-KEY (Irbesartan))	45,017
#8	(TITLE-ABS-KEY (Spironolactone) OR TITLE-ABS-KEY (Eplerenone))	29,976
#9	TITLE-ABS-KEY (Aliskiren)	2,730
#10	#6 OR #7 OR #8 OR #9	133,632
#11	#5 OR #10	201,108
#12	TITLE-ABS-KEY (renal AND replacement AND therapy) OR TITLE-ABS-KEY (dialysis) OR TITLE-ABS-KEY (renal AND dialysis) OR TITLE-ABS-KEY (peritoneal AND dialysis) OR TITLE-ABS-KEY (continuous AND ambulatory AND peritoneal AND dialysis) OR TITLE-ABS-KEY (automated AND peritoneal AND dialysis) OR TITLE-ABS-KEY (capd) OR TITLE-ABS-KEY (apd)	227,993
#13	(TITLE-ABS-KEY (renal AND function) OR TITLE-ABS-KEY (residual AND renal AND function) OR TITLE-ABS-KEY (glomerular AND filtration AND rate) OR TITLE-ABS-KEY (urine AND volume) OR TITLE-ABS-KEY (proteinuria) OR TITLE-ABS-KEY (membrane AND function) OR TITLE-ABS-KEY (ultrafiltration AND rate) OR TITLE-ABS-KEY (protein AND loss) OR TITLE-ABS-KEY (peritoneal AND equilibration AND test) OR TITLE-ABS-KEY (peritoneal AND membrane AND function) OR TITLE-ABS-KEY (peritoneal AND protein AND loss) OR TITLE-ABS-KEY (peritoneal AND dialysis AND adequacy) OR TITLE-ABS-KEY (adverse AND drug AND event) OR TITLE-ABS-KEY (adverse AND drug AND reaction) OR TITLE-ABS-KEY (drug AND side AND effect))	1,598,153
#14	#12 AND #13	46,035
#15	#11 AND #14	2,970
#16	(ALL (case reports) OR ALL (case series) OR ALL (comment) OR ALL (editorial) OR ALL (guideline) OR ALL (letter) OR ALL (meta-analysis) OR ALL (news) OR ALL (newspaper article) OR ALL (practice guideline))	25,067,658
#17	#15 AND NOT #16	838

Appendix-II. Systematic Review Search Strategy: Web of Science, From Inception to 31 May, 2019 (Continued)

Search Number	Input Terms	Items Found
#1	TS=((((((Angiotensin Converting Enzyme Inhibitors) OR Angiotensin I Converting Enzyme Inhibitors) OR Angiotensin Converting Enzyme Antagonists) OR ACE Inhibitors) OR Kininase II Antagonists) OR Kininase II Inhibitors)	26,197
#2	TS=(((((Angiotensin Receptor Blockers) OR Angiotensin Receptor Antagonists) OR Angiotensin II Receptor Antagonists) OR Angiotensin II Receptor Blockers) OR ARBs)	21,774
#3	TS=((((Aldosterone Receptor Antagonists) OR Aldosterone Antagonists) OR Mineralocorticoid Receptor Antagonists) OR Mineralocorticoid Antagonists)	4,270
#4	TS=((Renin Inhibitor) OR Direct Renin Inhibitors)	9,399
#5	#1 OR #2 OR #3 OR #4	42,587
#6	TS=((((((((((Benazepril) OR Captopril) OR Enalapril) OR Fosinopril) OR Lisinopril) OR Moexipril) OR Perindopril) OR Quinapril) OR Ramipril) OR Trandolapril)	15,510
#7	TS=((((((((Eprosartan) OR Olmesartan) OR Valsartan) OR Losartan) OR Telmisartan) OR Candesartan) OR Azilsartan) OR Irbesartan)	20,670
#8	TS=((Spironolactone) OR Eplerenone)	5,818
#9	TS=Aliskiren	1,679
#10	#6 OR #7 OR #8 OR #9	39,134
#11	#5 OR #10	64,288
#12	TS=((((((((Renal Replacement Therapy) OR Dialysis) OR Renal Dialysis) OR Peritoneal Dialysis) OR Continuous Ambulatory Peritoneal Dialysis) OR Automated Peritoneal Dialysis) OR CAPD) OR APD)	91,071
#13	TS=((((((((((((((Renal function) OR Residual renal function) OR Glomerular filtration rate) OR Urine volume) OR Proteinuria) OR Membrane function) OR Ultrafiltration rate) OR Protein loss) OR Peritoneal equilibration test) OR Peritoneal membrane function) OR Peritoneal protein loss) OR Peritoneal dialysis adequacy) OR Adverse drug event) OR Adverse drug reaction) OR Drug Side Effect)	550,836
#14	#12 AND #13	19,296
#15	#11 AND #14	642
#16	TS=((((((((((case reports) OR case series) OR comment) OR editorial) OR guideline) OR letter) meta-analysis) OR news) OR newspaper article) OR practical guideline)	86,937
#17	#15 NOT #16	639

Appendix-II. Systematic Review Search Strategy: CINAHL, From Inception to 31 May, 2019 (Continued)

Search Number	Input Terms	Items Found
#1	AB ((((((Angiotensin Converting Enzyme Inhibitors) OR Angiotensin I Converting Enzyme Inhibitors) OR Angiotensin Converting Enzyme Antagonists) OR ACE Inhibitors) OR Kininase II Antagonists) OR Kininase II Inhibitors)	16,700
#2	AB (((((Angiotensin Receptor Blockers) OR Angiotensin Receptor Antagonists) OR Angiotensin II Receptor Antagonists) OR Angiotensin II Receptor Blockers) OR ARBs)	15,728
#3	AB ((((Aldosterone Receptor Antagonists) OR Aldosterone Antagonists) OR Mineralocorticoid Receptor Antagonists) OR Mineralocorticoid Antagonists)	2,252
#4	AB ((Renin Inhibitor) OR Direct Renin Inhibitors)	4,411
#5	S1 OR S2 OR S3 OR S4	29,284
#6	AB ((((((((((Benazepril) OR Captopril) OR Enalapril) OR Fosinopril) OR Lisinopril) OR Moexipril) OR Perindopril) OR Quinapril) OR Ramipril) OR Trandolapril)	9,808
#7	AB ((((((((Eprosartan) OR Olmesartan) OR Valsartan) OR Losartan) OR Telmisartan) OR Candesartan) OR Azilsartan) OR Irbesartan)	12,023
#8	AB ((Spironolactone) OR Eplerenone)	3,057
#9	AB (Aliskiren)	886
#10	S6 OR S7 OR S8 OR S9	23,528
#11	S5 OR S10	9,666
#12	AB ((((((((Renal Replacement Therapy) OR Dialysis) OR Renal Dialysis) OR Peritoneal Dialysis) OR Continuous Ambulatory Peritoneal Dialysis) OR Automated Peritoneal Dialysis) OR CAPD) OR APD)	63,287
#13	AB ((((((((((((((Renal function) OR Residual renal function) OR Glomerular filtration rate) OR Urine volume) OR Proteinuria) OR Membrane function) OR Ultrafiltration rate) OR Protein loss) OR Peritoneal equilibration test) OR Peritoneal membrane function) OR Peritoneal protein loss) OR Peritoneal dialysis adequacy) OR Adverse drug event) OR Adverse drug reaction) OR Drug Side Effect)	355,134
#14	S12 AND S13	9,377
#15	S11 AND S14	56

Appendix-II. Systematic Review Search Strategy: Cochrane Library, From Inception to 31 May, 2019 (Continued)

Search Number	Input Terms	Items Found
#1	((((((Angiotensin Converting Enzyme Inhibitors) OR Angiotensin I Converting Enzyme Inhibitors) OR Angiotensin Converting Enzyme Antagonists) OR ACE Inhibitors) OR Kininase II Antagonists) OR Kininase II Inhibitors)	6,976
#2	(((((Angiotensin Receptor Blockers) OR Angiotensin Receptor Antagonists) OR Angiotensin II Receptor Antagonists) OR Angiotensin II Receptor Blockers) OR ARBs)	4,147
#3	((((Aldosterone Receptor Antagonists) OR Aldosterone Antagonists) OR Mineralocorticoid Receptor Antagonists) OR Mineralocorticoid Antagonists)	1,302
#4	((Renin Inhibitor) OR Direct Renin Inhibitors)	1,738
#5	#1 OR #2 OR #3 OR #4	10,621
#6	((((((((((Benazepril) OR Captopril) OR Enalapril) OR Fosinopril) OR Lisinopril) OR Moexipril) OR Perindopril) OR Quinapril) OR Ramipril) OR Trandolapril)	9,470
#7	((((((((Eprosartan) OR Olmesartan) OR Valsartan) OR Losartan) OR Telmisartan) OR Candesartan) OR Azilsartan) OR Irbesartan)	7,669
#8	((Spironolactone) OR Eplerenone)	2,071
#9	Aliskiren	675
#10	#6 OR #7 OR #8 OR #9	17,746
#11	#5 OR #10	21,573
#12	((((((((Renal Replacement Therapy) OR Dialysis) OR Renal Dialysis) OR Peritoneal Dialysis) OR Continuous Ambulatory Peritoneal Dialysis) OR Automated Peritoneal Dialysis) OR CAPD) OR APD)	17,498
#13	((((((((((((((Renal function) OR Residual renal function) OR Glomerular filtration rate) OR Urine volume) OR Proteinuria) OR Membrane function) OR Ultrafiltration rate) OR Protein loss) OR Peritoneal equilibration test) OR Peritoneal membrane function) OR Peritoneal protein loss) OR Peritoneal dialysis adequacy) OR Adverse drug event) OR Adverse drug reaction) OR Drug Side Effect)	171,840
#14	#12 AND #13	6,190
#15	#11 AND #14	504

eReferences

1. Higgins J, Sterne J, Savović J*, et al.* A revised tool for assessing risk of bias in randomized trials In: Chandler J, McKenzie J, Boutron I, et al., (eds). *Cochrane Methods.* : Cochrane Database of Systematic Reviews 2016, Issue 10 (Suppl 1). dx.doi.org/10.1002/14651858.CD201601.)
2. Wells G, Shea B, O'Connell D*, et al. The Newcastle-Ottawa Scale (NOS) for assessing the quality of nonrandomised studies in meta-analyses*. http://www.ohri.ca/programs/clinical_epidemiology/oxford.asp.
3. Puhan MA, Schunemann HJ, Murad MH*, et al.* A GRADE Working Group approach for rating the quality of treatment effect estimates from network meta-analysis. BMJ 2014;349:g5630
4. Berkman ND, Lohr KN, Ansari MT*, et al.* Grading the strength of a body of evidence when assessing health care interventions: an EPC update. J Clin Epidemiol 2015;68(11):1312-1324
5. Wan X, Wang W, Liu J*, et al.* Estimating the sample mean and standard deviation from the sample size, median, range and/or interquartile range. BMC Med Res Methodol 2014;14:135
6. Szeto CC, Kwan BC, Chow KM*, et al.* Predictors of residual renal function decline in patients undergoing continuous ambulatory peritoneal dialysis. Perit Dial Int 2015;35(2):180-188
7. Cohen J. *Statistical power analysis for the behavioral sciences*. 2nd ed. Lawrence Erlbaum Associates, Placed Published: 1988.
8. Friedrich JO, Adhikari NK, Beyene J. Inclusion of zero total event trials in meta-analyses maintains analytic consistency and incorporates all available data. BMC Med Res Methodol 2007;7:5
9. DerSimonian R, Laird N. Meta-analysis in clinical trials. Control Clin Trials 1986;7(3):177-188
10. Barth J, Munder T, Gerger H*, et al.* Comparative efficacy of seven psychotherapeutic interventions for patients with depression: a network meta-analysis. PLoS Med 2013;10(5):e1001454
11. Higgins JP, Thompson SG, Deeks JJ*, et al.* Measuring inconsistency in meta-analyses. BMJ 2003;327(7414):557-560
